# Genomic Evidence on Antihypertensive Drug Targets and Oral Disease Outcomes

**DOI:** 10.1111/jre.70143

**Published:** 2026-06-28

**Authors:** Aiswarya Puzhakkara Chennas, Ignacio Leiva‐Escobar, Stefan Lars Reckelkamm, Birte Holtfreter, Thomas Kocher, Sebastian‐Edgar Baumeister, Michael Nolde, Zoheir Alayash

**Affiliations:** ^1^ Institute of Health Services Research in Dentistry University of Münster Münster Germany; ^2^ Clinic for Periodontology and Conservative Dentistry University of Münster Münster Germany; ^3^ Department of Restorative Dentistry, Periodontology and Endodontology University Medicine Greifswald Greifswald Germany

**Keywords:** antihypertensive agents, dental caries, Mendelian randomization analysis, periodontitis

## Abstract

**Aim:**

Antihypertensive medications are widely prescribed for hypertension, yet evidence regarding their oral health safety remains limited. We aimed to investigate whether genetically proxied pharmacological actions of antihypertensive drug classes influence the risk of periodontitis and dental caries using a two‐sample drug‐target Mendelian randomization framework.

**Methods:**

Genetic instruments were obtained for 12 antihypertensive drug classes using variants within or near target genes associated with systolic blood pressure (SBP) in two genome‐wide association study (GWAS) meta‐analyses: one combining data from the International Consortium of Blood Pressure and UK Biobank, and another additionally including the Million Veteran Program (MVP) and Vanderbilt University's biorepository. Summary statistics for chronic periodontitis were obtained from the MVP GWAS, and for dental caries from the Gene‐Lifestyle Interactions in Dental Endpoints consortium using the decayed, missing, and filled surfaces index. Causal effects were estimated using the inverse‐variance weighted method with Benjamini–Hochberg correction for multiple testing. Colocalization analyses assessed whether drug targets and outcomes shared causal variants.

**Results:**

Genetically proxied diazoxide acting through the KCNJ11 locus was associated with chronic periodontitis after multiple‐testing correction (odds ratio [OR] per 1 mmHg lower SBP: 1.06, 95% confidence interval [CI]: 1.02–1.09), with consistent estimates after excluding variants associated with type 2 diabetes. No other associations were observed. Colocalization analyses provided no evidence of shared causal variants.

**Conclusion:**

We found little evidence that genetically proxied on‐target pharmacological actions of antihypertensive drug classes were associated with periodontitis or dental caries. The diazoxide–periodontitis association should be interpreted with caution, given possible residual pleiotropy at the KCNJ11 locus and the absence of colocalization.

## Clinical Relevance Box

**Background:**

Observational studies have reported associations between antihypertensive drug classes and periodontitis or dental caries; however, these findings remain difficult to interpret as conventional observational designs are vulnerable to confounding and reverse causation.

**Added Value of this Study:**

This study used a drug‐target Mendelian randomization framework to evaluate 12 antihypertensive drug classes in relation to chronic periodontitis and dental caries. It incorporated a large genome‐wide association study of chronic periodontitis, a quantitative dental caries phenotype, and complementary colocalization analyses.

**Clinical Implications:**

The findings provide little evidence that the on‐target pharmacological actions of most antihypertensive drug classes are associated with chronic periodontitis or dental caries. These findings should be interpreted in the context of a drug‐target Mendelian randomization design, which reflects lifelong genetically proxied perturbation of antihypertensive drug targets rather than direct evaluation of medication use. The association observed for diazoxide and chronic periodontitis should be interpreted with caution, as colocalization did not support a shared causal variant. In addition, diazoxide use is limited in current hypertension management.

## Key Findings

Drug‐target Mendelian randomization showed little evidence that genetically proxied on‐target pharmacological actions of most antihypertensive drug classes were associated with chronic periodontitis or dental caries. The diazoxide–periodontitis association requires cautious interpretation, given possible residual pleiotropy at KCNJ11 and lack of colocalization support. Graphical abstract created with BioRender.com.

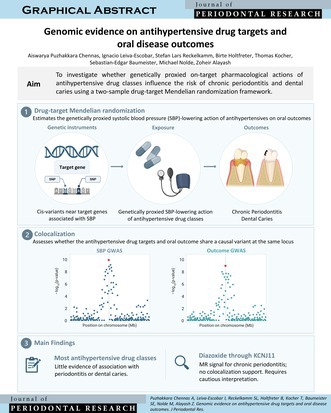

## 1 Introduction

Periodontitis is a chronic inflammatory disease driven by a dysbiotic polymicrobial biofilm, characterized by breakdown of the periodontal ligament and resorption of alveolar bone [1]. Dental caries is a multifactorial infectious disease characterized by the metabolism of fermentable carbohydrates by biofilm bacteria, resulting in the production of organic acids that demineralize the hard tissues of the teeth [2]. Periodontitis and dental caries are the most prevalent oral diseases, affecting approximately one and two billion individuals, respectively [3]. If untreated, both conditions may lead to tooth loss, compromising masticatory function, esthetics, and quality of life [4, 5].

Periodontitis is associated with several systemic disorders, including cardiovascular diseases, type 2 diabetes mellitus (T2D), rheumatoid arthritis, and Alzheimer's disease [6]; emerging evidence also suggests an association with hypertension [7]. Hypertension is a prevalent global health condition and a major risk factor for cardiovascular morbidity and mortality [8]. Beyond any direct relationship between periodontitis and hypertension, the pharmacological management of hypertension itself may influence oral health.

Several antihypertensive drug classes have been associated with xerostomia and/or reduced salivary flow rate. These include angiotensin‐converting enzyme inhibitors (ACEi), beta‐adrenoceptor antagonists (BBs), calcium channel blockers (CCBs), centrally acting antihypertensives, and diuretics, though the strength of evidence varies across classes [9]. Reduced salivary flow can compromise saliva's protective functions, including mechanical clearance of bacteria, buffering capacity, and antimicrobial activity, potentially increasing susceptibility to periodontal inflammation and dental caries in patients receiving antihypertensive medications [10].

Observational studies suggest that certain antihypertensive medications may be associated with periodontitis. ACEi have been positively associated with periodontitis [11, 12]. Similarly, angiotensin‐II receptor blockers (ARBs) [13] and CCBs [14] have each been linked to periodontitis. Diuretic and BB use have also been associated with increased dental caries [15, 16]. However, conventional observational designs are susceptible to confounding, reverse causation, and lack a temporal structure, limiting causal inference [17]. Moreover, available longitudinal data offer limited support for causal relationships [15].

Mendelian randomization (MR) employs an instrumental variable (IV) approach to assess the causal association between exposures and outcomes. It leverages single‐nucleotide polymorphisms (SNPs) as IVs to proxy exposures and assess their causal effects on disease outcomes [18]. MR is less susceptible to bias due to confounding that may affect conventional observational studies, as genetic variants segregate independently and assort randomly at conception. MR also minimizes the risk of reverse causation, given that genetic variants are fixed at birth and thus precede the outcome [19].

Recently, drug‐target MR, which restricts instruments to cis‐acting variants within or near druggable gene regions, has emerged as an important tool in drug development. It supports drug target discovery and validation, the evaluation of on‐target drug safety, and the assessment of repurposing potential [20]. Additionally, colocalization analyses strengthen causal inference in drug‐target MR by assessing whether genetic signals for the drug target and outcome share causal variants [21].

Prior drug‐target MR studies have examined the association between antihypertensive drug classes and periodontitis [22] and dental caries [23]. We extended previous work by leveraging a larger genome‐wide association study (GWAS) of periodontitis, incorporating quantitative dental caries phenotypes, and performing a comprehensive evaluation across antihypertensive classes. We employed drug‐target MR to assess whether genetically proxied on‐target pharmacological actions of antihypertensive drug targets are associated with oral disease outcomes, with colocalization analyses to strengthen causal inference.

## 2 Methods

### 2.1 Study Design

**FIGURE 1:**
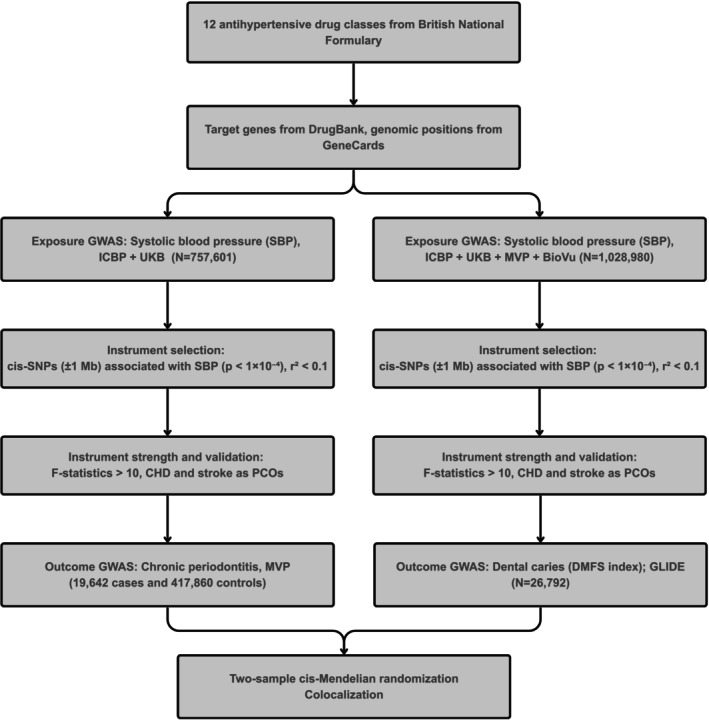
Overview of the study design. Twelve antihypertensive drug classes were identified from the British National Formulary, and corresponding target genes were obtained from DrugBank, with genomic positions retrieved from GeneCards. Summary statistics for systolic blood pressure (SBP) were obtained from two large meta‐analyses of genome‐wide association studies (GWAS) to limit sample overlap. The SBP dataset from the UK Biobank (UKB) and International Consortium for Blood Pressure (ICBP) was used for the chronic periodontitis analyses, whereas an extended meta‐analysis including UKB, ICBP, the Million Veteran Program (MVP), and Vanderbilt University's BioVU was used for the dental caries analyses. Cis‐acting single‐nucleotide polymorphisms (cis‐SNPs) located within ±1 Mb of each target gene, associated with SBP at *p* < 1 × 10^−4^ and linkage disequilibrium *r* ^2^ < 0.1 were selected as genetic instruments. Major coronary heart disease (CHD) events and stroke were included as positive control outcomes (PCOs). Two sample cis‐Mendelian randomization analyses were conducted to assess whether genetically proxied pharmacological action of antihypertensive drug classes was associated with chronic periodontitis (MVP; 19 642 cases and 417 860 controls) and dental caries (decayed, missing, and filled surfaces [DMFS]; gene‐lifestyle interactions in dental endpoints [GLIDE]; *N* = 26 792). Colocalization analyses were performed to evaluate whether these associations were driven by shared causal variants.

We conducted a two‐sample drug‐target MR study using publicly available GWAS summary statistics from individuals of European ancestry. As no individual‐level data was accessed, additional ethical approval was not required. We evaluated 12 antihypertensive drug classes for associations with chronic periodontitis and dental caries. Coronary heart disease (CHD) and stroke were included as positive control outcomes to validate instrument selection. Colocalization analyses complemented the MR framework. An overview of the study design is provided in Figure 1.

### 2.2 GWAS Data Sources

#### 2.2.1 Exposure: Systolic Blood Pressure

Summary statistics for systolic blood pressure (SBP) were obtained from two separate GWAS meta‐analyses to avoid sample overlap with outcome datasets. The first included 757 601 individuals from the International Consortium of Blood Pressure (ICBP) and the UK Biobank and was used for analyses of chronic periodontitis [24]. The second, an extended GWAS meta‐analysis, comprised 1 028 980 individuals, additionally including data from the Million Veteran Program (MVP) and the Vanderbilt University's biorepository, and was used for analyses of dental caries [25]. SBP was generally derived from repeated blood pressure measurements, although measurement protocols varied across contributing cohorts. Further information on SBP measurements, genotyping methods, and covariate adjustments is described in the original publications.

#### 2.2.2 Outcome: Chronic Periodontitis

Summary data for chronic periodontitis were retrieved from the MVP, with 19 642 cases and 417 860 controls. Cases were defined using Phecodes, which map ICD‐9 or ICD‐10‐Clinical Modification (CM) codes from electronic health records and require ≥ 2 instances of the corresponding codes for classification [26].

Chronic periodontitis was identified using Phecode 523.32, which maps to ICD‐9 codes 522.6 and 523.4 and ICD‐10‐CM codes K04.5, K05.3, K05.30, K05.31, K05.311, K05.312, K05.313, K05.319, K05.32, K05.321, K05.322, K05.323, and K05.329 [27, 28].

#### 2.2.3 Outcome: Dental Caries

Summary data for dental caries were drawn from the Gene‐Lifestyle Interactions in Dental Endpoints (GLIDE) consortium. Dental caries was measured as the decayed, missing, and filled surfaces (DMFS) index in 26 792 individuals using clinical dental records [29].

#### 2.2.4 Positive Control Outcomes: Coronary Heart Disease and Stroke

Summary statistics for major CHD events and stroke were obtained from the FinnGen R12 release, comprising 56 650 CHD cases and 443 698 controls, and 34 110 stroke cases and 450 023 controls, respectively [30].

### 2.3 Instrument Selection

A list of antihypertensive drug classes was compiled using the British National Formulary. We identified 12 antihypertensive drug classes: adrenergic neuron blockers, alpha‐adrenoceptor antagonists, ACEi, ARBs, BBs, CCBs, centrally acting antihypertensives, loop diuretics, potassium‐sparing diuretics and aldosterone antagonists, renin inhibitors, thiazides and related diuretics, and vasodilator antihypertensives. Vasodilator antihypertensives, including hydralazine, minoxidil, fenoldopam, diazoxide, and nitroprusside, were evaluated independently rather than as a unified antihypertensive class, as they do not share common drug targets and act through distinct mechanisms [31, 32]. Details of target genes for each drug class are provided in Table S1.

DrugBank was used to determine target genes shared across drugs within each antihypertensive class, and their genomic coordinates were sourced from GeneCards [32]. Cis‐acting variants associated with SBP were selected to proxy each antihypertensive class. SNP–SBP associations were obtained from two SBP GWASs described in Section 2.2.1 to limit sample overlap. The SNP‐outcome associations were extracted from GWASs of chronic periodontitis and dental caries.

We selected SNPs within a ±1 Mb window of the corresponding target genes that were associated with SBP at *p* < 1 × 10^−4^. Since our study examined SNPs at defined loci, we did not apply the conventional genome‐wide significance threshold [33]. SNPs were clumped at *r* ^2^ < 0.1 using the 1000 Genomes European reference panel to ensure low linkage disequilibrium (LD) and thereby limit correlated instruments. Such cis‐acting SNPs are more likely to influence the function of their corresponding gene and are less prone to pre‐translational horizontal pleiotropy [34].

Across antihypertensive drug classes, between 1 and 81 SNPs were identified as genetic proxies for analyses of chronic periodontitis (Tables S2–S17), and between 4 and 104 SNPs were identified for analyses of dental caries (Tables S18–S33).

### 2.4 Instrument Strength

The validity of MR depends on three core IV assumptions: (1) relevance (robust association with the exposure), (2) exchangeability (independence from confounders of the exposure‐outcome pathway), and (3) exclusion restriction (effect on the outcome only through the exposure, i.e., no horizontal pleiotropy) [19].

We calculated the proportion of variance (*R* ^2^) explained by the genetic instruments for each antihypertensive drug class and the corresponding *F*‐statistics. The *F*‐statistic was used to evaluate instrument strength; values greater than 10 were interpreted as indicating lower risk of weak instrument bias [35].

All genetic instruments had *F*‐statistics greater than 10 (Tables S2–S33).

### 2.5 Instrument Validation

Genetic variants in KCNJ11 (potassium inwardly rectifying channel subfamily J member 11), the target gene for diazoxide, have been associated with type 2 diabetes mellitus (T2D), a known risk factor for periodontitis [36]. To assess potential pleiotropic effects via glycaemic pathways, a sensitivity analysis was conducted for diazoxide and periodontitis, excluding KCNJ11 variants previously associated with T2D.

Major CHD events and stroke were used as positive control outcomes to assess whether SNPs selected as proxies for antihypertensive drug classes produced directionally consistent protective associations of SBP lowering on cardiovascular disease.

### 2.6 Statistical Analyses

#### 2.6.1 Mendelian Randomization Analyses

Exposure and outcome data were harmonized by aligning SNP‐exposure and SNP‐outcome associations to the same effect allele. Palindromic and ambiguous SNPs were removed at this stage. If a SNP was missing in the outcome data, a proxy SNP in strong pairwise LD (*r* ^2^ > 0.8) was identified; if no suitable proxy was available, the SNP was excluded from the analysis.

The Wald ratio for each SNP was calculated by dividing the SNP‐outcome association by the SNP‐exposure association. For antihypertensive classes represented by a single SNP, the causal estimate was obtained using the Wald ratio. When multiple SNPs were available, individual Wald ratios were pooled using the inverse‐variance weighted (IVW) fixed‐effects method. Heterogeneity within each antihypertensive class was assessed using Cochran's Q test; when significant heterogeneity was observed, the IVW multiplicative random‐effects method was applied [37].

Results for periodontitis are presented as odds ratios (ORs) per 1 mmHg reduction in SBP for each antihypertensive class. Estimates for dental caries are reported as beta coefficients representing the change in the transformed DMFS score (one unit change in the transformed DMFS score corresponds to an increase of 19.87 affected surfaces) per 1 mmHg reduction in SBP [38]. The Benjamini–Hochberg procedure was applied to account for multiple testing, and false discovery rate (FDR) adjusted *p*‐values were reported [39]. These values were calculated across 32 tests (11 antihypertensive drug classes and 5 vasodilator antihypertensives, each tested against 2 outcomes). Results with an FDR‐adjusted *p* < 0.05 were considered statistically significant. Additionally, leave‐one‐out analyses assessed whether MR estimates were driven by a single instrument.

Consistent with recent cis‐MR guidance, pleiotropy‐robust MR methods commonly used in genome‐wide MR were not employed because they may provide limited additional information in cis‐MR analyses. Weighted median, weighted mode, and MR‐PRESSO (MR pleiotropy residual sum and outlier) are more informative in genome‐wide MR settings where instruments are independent, and pleiotropic effects may vary across variants. Modeling‐based approaches, such as MR‐Egger, may also be constrained by the number of available instruments in a cis‐MR setting [40].

#### 2.6.2 Colocalization Analysis

We investigated whether instruments for antihypertensive drug classes shared causal variants with chronic periodontitis or dental caries through colocalization analyses. These analyses distinguish true shared causal variants within a locus from associations that may arise due to LD‐linked pleiotropy. We first applied the traditional Bayesian colocalization method, which assumes a single causal variant within each genomic locus [21]. As multiple causal variants may be shared within a locus, we also applied the sum of single effects (SuSiE) regression framework, which allows for multiple causal variants within a locus [41].

Colocalization analyses were performed by including variants with a ±1 Mb window around the target genes for each antihypertensive class. We used default priors for the analyses: p1 = 1 × 10^−4^, the prior probability that a SNP is associated with SBP within a drug target locus, p2 = 1 × 10^−4^, the prior probability that a SNP is associated with periodontitis or dental caries within a drug target locus, and p12 = 1 × 10^−5^, the prior probability that a SNP is associated with both traits. These priors were used to calculate the posterior probability that the two traits shared causal variants. A posterior probability of shared causal variants (PP.H4) ≥ 0.9, determined by at least one method, was considered strong evidence for colocalization [21].

All statistical analyses were performed in R version 4.4.3 (R Foundation for Statistical Computing) using the TwoSampleMR and coloc packages. The study adhered to STROBE‐MR guidelines [42] and the Principled Framework for Mendelian Randomization in Oral Health Research [43].

## 3 Results

### 3.1 Mendelian Randomization Analyses

#### 3.1.1 Chronic Periodontitis

Results of the IVW MR analyses for chronic periodontitis are presented in Figure 2. Leave‐one‐out analyses for chronic periodontitis are presented in Figures S1–S15. Diazoxide, instrumented through variants in KCNJ11, was associated with chronic periodontitis after correction for multiple testing (OR per 1 mmHg lower SBP: 1.06; 95% confidence interval [CI]: 1.02–1.09; FDR‐adjusted *p* = 0.03). This association remained consistent in leave‐one‐out analyses (Figure S12), and after excluding KCNJ11 variants that are associated with T2D (OR per 1 mmHg lower SBP: 1.05; 95% CI: 1.01–1.10; *p* = 0.01) (Table S34).

**FIGURE 2:**
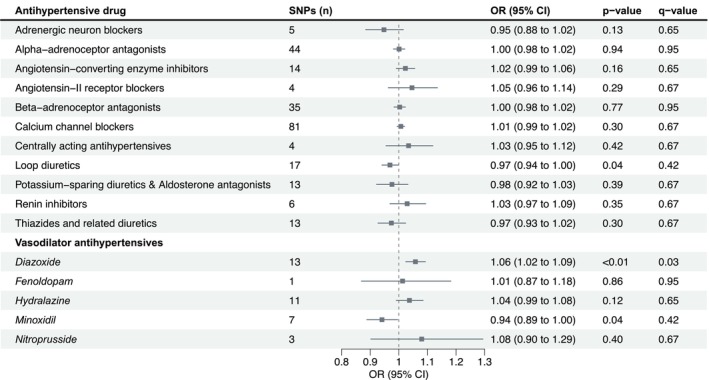
Forest plot of inverse‐variance weighted Mendelian randomization estimates for the associations between antihypertensive drug classes and chronic periodontitis. Odds ratios (ORs) represent the effect of a genetically proxied 1 mmHg reduction in systolic blood pressure (SBP) through different antihypertensive drug classes on the risk of periodontitis. SNPs (*n*) indicate the number of single‐nucleotide polymorphisms used as instruments for each antihypertensive class. OR, odds ratio; CI, confidence interval; *q*‐value, false discovery rate‐adjusted *p*‐value.

No other antihypertensive drug classes were associated with chronic periodontitis after correction for multiple testing (Figure 2). Heterogeneity was observed in effect estimates for potassium‐sparing diuretics and aldosterone antagonists, thiazide and related diuretics, and nitroprusside; therefore, results from the IVW multiplicative random‐effects model are reported (Table S35).

#### 3.1.2 Dental Caries

Results of the IVW MR analyses for dental caries are presented in Figure 3. Leave‐one‐out analyses for dental caries are presented in Figures S16–S31. No antihypertensive drug classes were associated with dental caries. Heterogeneity in effect estimates was observed for BBs and minoxidil; results from the IVW multiplicative random‐effects model are therefore reported (Table S36).

**FIGURE 3:**
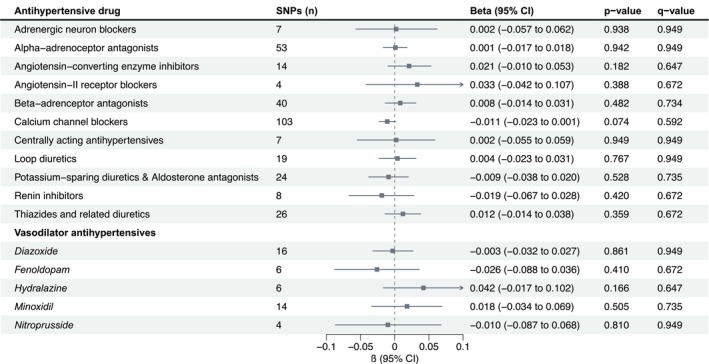
Forest plot of inverse‐variance weighted Mendelian randomization estimates for the associations between antihypertensive drug classes and dental caries. Betas represent the change in the transformed DMFS (Decayed, Missing, and Filled Surfaces) score per genetically proxied 1 mmHg reduction in systolic blood pressure (SBP) through different antihypertensive drug classes. SNPs (*n*) indicate the number of single‐nucleotide polymorphisms used as instruments for each antihypertensive class. Beta, beta‐coefficients; CI, confidence interval; *q*‐value, false discovery rate–adjusted *p*‐value.

### 3.2 Colocalization

Bayesian colocalization analysis suggested that the genetically proxied action of diazoxide was unlikely to share a causal variant with chronic periodontitis within the KCNJ11 locus (posterior probability of shared causal variants [PP.H4]: 3.18%, posterior probability of distinct causal variants [PP.H3]: 10.10%). Similarly, no other antihypertensive drug targets showed evidence of colocalization with either outcome (Tables S37 and S38). SuSiE‐based colocalization did not identify credible sets for chronic periodontitis or dental caries.

### 3.3 Positive Control Analyses

Most antihypertensive classes showed directionally consistent protective associations (Figures S32 and S33). For diazoxide, estimates were directionally consistent with SBP‐lowering effects for both CHD and stroke but were not statistically significant. Renin inhibitors, adrenergic neuron blockers, hydralazine, and minoxidil showed inconsistent associations in one or both outcomes; results for these drug targets were interpreted cautiously.

## 4 Discussion

In this two‐sample drug‐target MR study, we evaluated whether genetically proxied pharmacological action of antihypertensive drug targets is causally associated with chronic periodontitis and dental caries. Overall, no associations were observed between antihypertensive drug classes and either outcome. The exception was diazoxide, proxied through variants in KCNJ11, which was associated with chronic periodontitis. This association was robust to a sensitivity analysis excluding KCNJ11 variants associated with T2D and to leave‐one‐out analyses. However, diazoxide was not significantly associated with either CHD or stroke in the positive control analyses, although the estimates were directionally consistent with the expected protective effect of SBP lowering. Colocalization analyses also did not provide strong evidence for shared causal variants for the diazoxide–periodontitis association at the KCNJ11 locus or between any other antihypertensive drug targets and either outcome.

Our findings are inconsistent with prior MR studies examining antihypertensive medications and oral health. A previous study reported an association between loop diuretics and chronic periodontitis (OR per 1 mmHg lower SBP: 0.94, 95% CI: 0.90–0.98) [22], whereas we did not replicate this finding after multiple testing correction. Several methodological differences may account for these discrepancies. We used different instrument selection criteria and a larger periodontitis GWAS. Additionally, associations of CCBs (OR per 1 mmHg lower SBP: 0.97, 95% CI: 0.95–0.99) and loop diuretics (OR per 1 mmHg lower SBP: 0.93, 95% CI: 0.90–0.97) with dental caries defined as a binary phenotype were previously reported [23]. In contrast, we analyzed dental caries using a quantitative DMFS measure and used an SBP GWAS that further included individuals from the MVP and Vanderbilt University's biorepository. The inclusion of root caries in binary phenotypes may partly explain the variation in associations. Our null findings for renin inhibitors, adrenergic neuron blockers, hydralazine, and minoxidil should be interpreted with particular caution because, despite meeting predefined selection criteria, these drug targets showed inconsistent associations in positive control analyses for the corresponding oral disease outcomes.

Contrary to our predominantly null findings, observational studies have linked ACEi use to periodontitis, including higher odds of sites with probing depth ≥ 5 mm (OR: 3.21, 95% CI: 1.33–7.71) and clinical attachment loss ≥ 5 mm (OR: 2.98, 95% CI: 1.60–5.51) in a case–control study [12] and higher odds of periodontitis in a cross‐sectional analysis (OR: 4.85, 95% CI: 2.79–8.43) [11]. Additionally, ARBs were associated with greater periodontitis prevalence (OR: 3.57, 95% CI: 1.06–11.99) [13], and CCB use was associated with periodontal stage (OR: 0.47, 95% CI: 0.28–0.76) [14], in cross‐sectional studies. Diuretic use was associated with higher decayed, missing, and filled teeth (DMFT) scores in a cross‐sectional study (mean difference 2.16; *p* < 0.01) [16], and BB use with a greater coronal caries increment (beta‐coefficient: 2.35; standard error: 0.66; *p* < 0.01) [15] in a longitudinal study.

Methodological constraints inherent to observational studies may explain the contrast in findings. Many studies were cross‐sectional, and temporal precedence between medication use and outcome could not be established. Moreover, pharmacoepidemiological designs that mitigate time‐related biases were not implemented [44]. Studies were often modest in size and hospital‐based, with limited adjustment for confounders. Medication exposure and oral health outcomes were sometimes derived from self‐reported data or electronic health records without detailed information on dose, duration, polypharmacy, or standardized periodontal examinations. Although antihypertensive medications are considered xerogenic, only limited studies have incorporated objective measurements of salivary flow rate [9].

The diazoxide–periodontitis association requires careful interpretation given the pleiotropic nature of the KCNJ11 locus. KCNJ11 encodes Kir6.2, a subunit of the ATP‐sensitive potassium channel involved in insulin secretion in pancreatic beta‐cells [45]; genetic variation in this region has been associated with T2D [36], an established risk factor for periodontitis [6]. Results were consistent after excluding instruments associated with T2D; however, residual metabolic pleiotropy cannot be excluded, particularly given the lack of colocalization at the KCNJ11 locus. The clinical relevance of this finding is limited, as diazoxide is rarely prescribed for hypertension in contemporary practice.

A key strength of this study is the drug‐target MR framework, integrating biologically informed instrument selection, validation through positive control outcomes, colocalization analyses, and leveraging quantitative phenotypes of dental caries. Several limitations should be acknowledged. Analyses were conducted using GWAS summary statistics from individuals of European ancestry, and the MVP cohort was predominantly male, constraining the generalizability of these findings. Although repeated SBP measurements were used where available, variation in SBP assessment across contributing GWAS studies may have influenced SNP‐SBP association estimates and the interpretation of null findings; more uniform SBP measurement protocols may strengthen future evaluations. Chronic periodontitis in the MVP GWAS was based on electronic health record‐derived Phecodes and lacked information on staging and grading according to the 2018 World Workshop classification [46]; controls were not clinically confirmed to be free of periodontitis. Standardized clinical periodontal examinations with information on severity, extent and distribution would improve phenotype definition in future GWASs of periodontitis. Larger GWASs incorporating clinically assessed, quantitative caries phenotypes would provide greater power to detect modest target‐mediated associations. The heterogeneity observed in some cis‐MR analyses does not necessarily indicate that the instruments are invalid [40]. Rather, it may reflect within‐region variation among instruments, including local LD structure, effects of neighboring genes, or differences in how variants influence SBP through the target gene. Genetic instruments for some antihypertensives included multiple target genes; unequal functional contributions and potential interactions among these genes were not accounted for. Drug‐target MR estimates reflect lifelong genetic perturbation of targets and may not capture off‐target pharmacological effects [33] such as xerostomia and reduced salivary flow, which are plausible pathways linking antihypertensive medication use to oral disease outcomes.

## 5 Conclusions

Our findings provide limited support for associations between the genetically proxied on‐target pharmacological actions of antihypertensive classes and chronic periodontitis or dental caries. An association was observed for genetically proxied pharmacological action of diazoxide through the KCNJ11 locus and periodontitis; however, the interpretation of this finding is constrained by possible residual pleiotropy at the locus, lack of colocalization support, and limited contemporary use of diazoxide as an antihypertensive.

## Author Contributions

A. Puzhakkara Chennas contributed to conception and design, data acquisition, data analysis and interpretation, and drafted and critically revised the manuscript. S.‐E. Baumeister, M. Nolde, and Z. Alayash contributed to conception and design, data acquisition and interpretation, and drafted and critically revised the manuscript. I. Leiva‐Escobar, S.L. Reckelkamm, B. Holtfreter, and T. Kocher contributed to conception and design and critically revised the manuscript. All authors approved the final manuscript and agreed to be accountable for all aspects of the work.

## Funding

This study was supported by the Deutsche Forschungsgemeinschaft (DFG, grant number 552087827).

## Disclosure

AI Statement: This manuscript used natural language processing for proofreading an entirely human‐generated text, but no other use of artificial intelligence was made.

## Conflicts of Interest

The authors declare no conflicts of interest.

## Supporting information


**Table S1:** Antihypertensive drug classes and their corresponding target genes.
**Table S2:** Associations of cis single‐nucleotide polymorphisms (SNPs) used as instruments for adrenergic neuron blockers from the systolic blood pressure GWAS by Evangelou et al. [1].
**Table S3:** Associations of cis single‐nucleotide polymorphisms (SNPs) used as instruments for alpha‐adrenoceptor antagonists from the systolic blood pressure GWAS by Evangelou et al. [1].
**Table S4:** Associations of cis single‐nucleotide polymorphisms (SNPs) used as instruments for angiotensin‐converting enzyme inhibitors from the systolic blood pressure GWAS by Evangelou et al. [1].
**Table S5:** Associations of cis single‐nucleotide polymorphisms (SNPs) used as instruments for angiotensin‐II receptor blockers from the systolic blood pressure GWAS by Evangelou et al. [1].
**Table S6:** Associations of cis single‐nucleotide polymorphisms (SNPs) used as instruments for beta‐adrenoceptor antagonists from the systolic blood pressure GWAS by Evangelou et al. [1].
**Table S7:** Associations of cis single‐nucleotide polymorphisms (SNPs) used as instruments for calcium channel blockers from the systolic blood pressure GWAS by Evangelou et al. [1].
**Table S8:** Associations of cis single‐nucleotide polymorphisms (SNPs) used as instruments for centrally acting antihypertensives from the systolic blood pressure GWAS by Evangelou et al. [1].
**Table S9:** Associations of cis single‐nucleotide polymorphisms (SNPs) used as instruments for loop diuretics from the systolic blood pressure GWAS by Evangelou et al. [1].
**Table S10:** Associations of cis single‐nucleotide polymorphisms (SNPs) used as instruments for potassium‐sparing diuretics and aldosterone antagonists from the systolic blood pressure GWAS by Evangelou et al. [1].
**Table S11:** Associations of cis single‐nucleotide polymorphisms (SNPs) used as instruments for renin inhibitors from the systolic blood pressure GWAS by Evangelou et al. [1].
**Table S12:** Associations of cis single‐nucleotide polymorphisms (SNPs) used as instruments for thiazide and related diuretics from the systolic blood pressure GWAS by Evangelou et al. [1].
**Table S13:** Associations of cis single‐nucleotide polymorphisms (SNPs) used as instruments for diazoxide (a vasodilator antihypertensive) from the systolic blood pressure GWAS by Evangelou et al. [1].
**Table S14:** Associations of cis single‐nucleotide polymorphisms (SNPs) used as instruments for fenoldopam (a vasodilator antihypertensive) from the systolic blood pressure GWAS by Evangelou et al. [1].
**Table S15:** Associations of cis single‐nucleotide polymorphisms (SNPs) used as instruments for hydralazine (a vasodilator antihypertensive) from the systolic blood pressure GWAS by Evangelou et al. [1].
**Table S16:** Associations of cis single‐nucleotide polymorphisms (SNPs) used as instruments for minoxidil (a vasodilator antihypertensive) from the systolic blood pressure GWAS by Evangelou et al. [1].
**Table S17:** Associations of cis single‐nucleotide polymorphisms (SNPs) used as instruments for nitroprusside (a vasodilator antihypertensive) from the systolic blood pressure GWAS by Evangelou et al. [1].
**Table S18:** Associations of cis single‐nucleotide polymorphisms (SNPs) used as instruments for adrenergic neuron blockers from the systolic blood pressure GWAS by Keaton et al. [2].
**Table S19:** Associations of cis single‐nucleotide polymorphisms (SNPs) used as instruments for alpha‐adrenoceptor antagonists from the systolic blood pressure GWAS by Keaton et al. [2].
**Table S20:** Associations of cis single‐nucleotide polymorphisms (SNPs) used as instruments for angiotensin‐converting enzyme inhibitors from the systolic blood pressure GWAS by Keaton et al. [2].
**Table S21:** Associations of cis single‐nucleotide polymorphisms (SNPs) used as instruments for angiotensin‐II receptor blockers from the systolic blood pressure GWAS by Keaton et al. [2].
**Table S22:** Associations of cis single‐nucleotide polymorphisms (SNPs) used as instruments for beta‐adrenoceptor antagonists from the systolic blood pressure GWAS by Keaton et al. [2].
**Table S23:** Associations of cis single‐nucleotide polymorphisms (SNPs) used as instruments for calcium channel blockers from the systolic blood pressure GWAS by Keaton et al. [2].
**Table S24:** Associations of cis single‐nucleotide polymorphisms (SNPs) used as instruments for centrally acting antihypertensives from the systolic blood pressure GWAS by Keaton et al. [2].
**Table S25:** Associations of cis single‐nucleotide polymorphisms (SNPs) used as instruments for loop diuretics from the systolic blood pressure GWAS by Keaton et al. [2].
**Table S26:** Associations of cis single‐nucleotide polymorphisms (SNPs) used as instruments for potassium‐sparing diuretics and aldosterone antagonists from the systolic blood pressure GWAS by Keaton et al. [2].
**Table S27:** Associations of cis single‐nucleotide polymorphisms (SNPs) used as instruments for renin inhibitors from the systolic blood pressure GWAS by Keaton et al. [2].
**Table S28:** Associations of cis single‐nucleotide polymorphisms (SNPs) used as instruments for thiazides and related diuretics from the systolic blood pressure GWAS by Keaton et al. [2].
**Table S29:** Associations of cis single‐nucleotide polymorphisms (SNPs) used as instruments for diazoxide (a vasodilator antihypertensive) from the systolic blood pressure GWAS by Keaton et al. [2].
**Table S30:** Associations of cis single‐nucleotide polymorphisms (SNPs) used as instruments for fenoldopam (a vasodilator antihypertensive) from the systolic blood pressure GWAS by Keaton et al. [2].
**Table S31:** Associations of cis single‐nucleotide polymorphisms (SNPs) used as instruments for hydralazine (a vasodilator antihypertensive) from the systolic blood pressure GWAS by Keaton et al. [2].
**Table S32:** Associations of cis single‐nucleotide polymorphisms (SNPs) used as instruments for minoxidil (a vasodilator antihypertensive) from the systolic blood pressure GWAS by Keaton et al. [2].
**Table S33:** Associations of cis single‐nucleotide polymorphisms (SNPs) used as instruments for nitroprusside (a vasodilator antihypertensive) from the systolic blood pressure GWAS by Keaton et al. [2].
**Table S34:** KCNJ11 variants associated with type 2 diabetes excluded from the sensitivity analysis of diazoxide and chronic periodontitis.
**Table S35:** Heterogeneity statistics for inverse‐variance weighted Mendelian randomization analyses of antihypertensive drug classes and chronic periodontitis.
**Table S36:** Heterogeneity statistics for inverse‐variance weighted Mendelian randomization analyses of antihypertensive drug classes and dental caries.
**Table S37:** Bayesian colocalization results for 12 antihypertensive drug classes and chronic periodontitis.
**Table S38:** Bayesian colocalization results for 12 antihypertensive drug classes and dental caries.
**Figure S1:** Leave‐one‐out inverse‐variance weighted analyses plot for adrenergic neuron blockers and chronic periodontitis.
**Figure S2:** Leave‐one‐out inverse‐variance weighted analyses plot for angiotensin‐converting enzyme inhibitors and chronic periodontitis.
**Figure S3:** Leave‐one‐out inverse‐variance weighted analyses plot for alpha‐adrenoceptor antagonists and chronic periodontitis.
**Figure S4:** Leave‐one‐out inverse‐variance weighted analyses plot for angiotensin‐II receptor blockers and chronic periodontitis.
**Figure S5:** Leave‐one‐out inverse‐variance weighted analyses plot for beta‐adrenoceptor antagonists and chronic periodontitis.
**Figure S6:** Leave‐one‐out inverse‐variance weighted analyses plot for calcium channel blockers and chronic periodontitis.
**Figure S7:** Leave‐one‐out inverse‐variance weighted analyses plot for centrally acting antihypertensives and chronic periodontitis.
**Figure S8:** Leave‐one‐out inverse‐variance weighted analyses plot for loop diuretics and chronic periodontitis.
**Figure S9:** Leave‐one‐out inverse‐variance weighted analyses plot for potassium‐sparing diuretics and aldosterone antagonists and chronic periodontitis.
**Figure S10:** Leave‐one‐out inverse‐variance weighted analyses plot for renin inhibitors and chronic periodontitis.
**Figure S11:** Leave‐one‐out inverse‐variance weighted analyses plot for thiazides and related diuretics and chronic periodontitis.
**Figure S12:** Leave‐one‐out inverse‐variance weighted analyses plot for diazoxide and chronic periodontitis.
**Figure S13:** Leave‐one‐out inverse‐variance weighted analyses plot for hydralazine and chronic periodontitis.
**Figure S14:** Leave‐one‐out inverse‐variance weighted analyses plot for minoxidil and chronic periodontitis.
**Figure S15:** Leave‐one‐out inverse‐variance weighted analyses plot for nitroprusside and chronic periodontitis.
**Figure S16:** Leave‐one‐out inverse‐variance weighted analyses plot for adrenergic neuron blockers and dental caries.
**Figure S17:** Leave‐one‐out inverse‐variance weighted analyses plot for angiotensin‐converting enzyme inhibitors and dental caries.
**Figure S18:** Leave‐one‐out inverse‐variance weighted analyses plot for alpha‐adrenoceptor antagonists and dental caries.
**Figure S19:** Leave‐one‐out inverse‐variance weighted analyses plot for angiotensin‐II receptor blockers and dental caries.
**Figure S20:** Leave‐one‐out inverse‐variance weighted analyses plot for beta‐adrenoceptor antagonists and dental caries.
**Figure S21:** Leave‐one‐out inverse‐variance weighted analyses plot for calcium channel blockers and dental caries.
**Figure S22:** Leave‐one‐out inverse‐variance weighted analyses plot for centrally acting antihypertensives and dental caries.
**Figure S23:** Leave‐one‐out inverse‐variance weighted analyses plot for loop diuretics and dental caries.
**Figure S24:** Leave‐one‐out inverse‐variance weighted analyses plot for potassium‐sparing diuretics and aldosterone antagonists and dental caries.
**Figure S25:** Leave‐one‐out inverse‐variance weighted analyses plot for renin inhibitors and dental caries.
**Figure S26:** Leave‐one‐out inverse‐variance weighted analyses plot for thiazides and related diuretics and dental caries.
**Figure S27:** Leave‐one‐out inverse‐variance weighted analyses plot for diazoxide and dental caries.
**Figure S28:** Leave‐one‐out inverse‐variance weighted analyses plot for fenoldopam and dental caries.
**Figure S29:** Leave‐one‐out inverse‐variance weighted analyses plot for hydralazine and dental caries.
**Figure S30:** Leave‐one‐out inverse‐variance weighted analyses plot for minoxidil and dental caries.
**Figure S31:** Leave‐one‐out inverse‐variance weighted analyses plot for nitroprusside and dental caries.
**Figure S32:** Positive control analyses for the antihypertensive drug classes and chronic periodontitis: Associations between genetically proxied antihypertensive drug classes and major coronary heart disease events and stroke. Odds ratios represent the effect of a genetically proxied 1 mmHg reduction in systolic blood pressure (SBP) through different antihypertensive drug classes on the risk of coronary heart disease (CHD) and stroke. SNPs (n) indicate the number of single‐nucleotide polymorphisms used as instruments for each antihypertensive class. CI, confidence interval; OR, odds ratio.
**Figure S33:** Positive control analyses for the antihypertensive drug classes and dental caries: Associations between genetically proxied antihypertensive drug classes and major coronary heart disease events and stroke. Odds ratios represent the effect of a genetically proxied 1 mmHg reduction in systolic blood pressure (SBP) through different antihypertensive drug classes on the risk of coronary heart disease (CHD) and stroke. SNPs (n) indicate the number of single‐nucleotide polymorphisms used as instruments for each antihypertensive class. CI, confidence interval; OR, odds ratio.

## Data Availability

Systolic blood pressure GWAS summary statistics are available from the GWAS Catalog (https://www.ebi.ac.uk/gwas/) under the accession IDs GCST006624 and GCST90310294. FinnGen R12 release summary statistics for major coronary heart disease (CHD) events and stroke are available from the FinnGen consortium (https://www.finngen.fi/). Chronic periodontitis GWAS summary statistics from the Million Veteran Program (MVP) are available through dbGaP (accession number phs002453). Dental caries GWAS summary statistics from the GLIDE consortium are available at https://data.bris.ac.uk/data/dataset/2j2rqgzedxlq02oqbb4vmycnc2.
