# Supplementary material for: Genomic Evidence on Antihypertensive Drug Targets and Oral Disease Outcomes
Source: J Periodontal Res. 2026 Jun 28;61(6):695–704. doi: 10.1111/jre.70143 (PMC13471875; doi:10.1111/jre.70143)
Supplement: Supplementary file 1 [file JRE-61-695-s001.docx]

**Appendix**

**Genomic evidence on the oral health safety of antihypertensive medications**

Aiswarya Puzhakkara Chennas^1^, Ignacio Leiva-Escobar^1^, Stefan Lars Reckelkamm^1,2^, Birte Holtfreter^3^, Thomas Kocher^3^, Sebastian-Edgar Baumeister^1^, Michael Nolde^1^, Zoheir Alayash^1^

^1^ Institute of Health Services Research in Dentistry, University of Münster, Münster, Germany

^2^ Clinic for Periodontology and Conservative Dentistry, University of Münster, Münster, Germany.

^3^ Department of Restorative Dentistry, Periodontology and Endodontology, University Medicine Greifswald, Greifswald, Germany.

**Appendix Tables**

**Appendix Table 1	Pages 4-8**

Antihypertensive drug classes and their corresponding target genes

**Appendix Tables 2-17	Pages 9-28**

Associations of cis single-nucleotide polymorphisms (SNPs) used as instruments for antihypertensive drug classes from the systolic blood pressure GWAS by Evangelou et al. 2018 for analyses of chronic periodontitis.

SNPs listed represent instruments identified prior to harmonization.

**Appendix Tables 18-33	Pages 29-49**

Associations of cis single-nucleotide polymorphisms (SNPs) used as instruments for antihypertensive drug classes from the systolic blood pressure GWAS by Keaton et al. 2024 for analyses of dental caries.

SNPs listed represent instruments identified prior to harmonization.

**Appendix Table 34	Page 50**

KCNJ11 variants associated with type 2 diabetes excluded from the sensitivity analysis of diazoxide and chronic periodontitis

**Appendix Table 35	Page 51**

Heterogeneity statistics for inverse-variance weighted Mendelian randomization analyses of antihypertensive drug classes and chronic periodontitis

**Appendix Table 36	Page 52**

Heterogeneity statistics for inverse-variance weighted Mendelian randomization analyses of antihypertensive drug classes and dental caries

**Appendix Table 37	Page 53-55**

Bayesian colocalization results for 12 antihypertensive drug classes and chronic periodontitis

**Appendix Table 38	Pages 56-58**

Bayesian colocalization results for 12 antihypertensive drug classes and dental caries

**Appendix Figures**

**Appendix Figures 1-15	Pages 59-67**

Leave-one-out inverse-variance weighted (IVW) analyses plots for antihypertensive drug classes and chronic periodontitis

**Appendix Figures 16-31	Pages 68-76**

Leave-one-out inverse-variance weighted (IVW) analyses plots for antihypertensive drug classes and dental caries

**Appendix Figure 32	Page 77**

Positive control analyses for the antihypertensive drug classes and chronic periodontitis: Associations between genetically proxied antihypertensive drug classes and major coronary heart disease (CHD) and stroke

**Appendix Figure 33	Page 78**

Positive control analyses for the antihypertensive drug classes and dental caries: Associations between genetically proxied antihypertensive drug classes and major coronary heart disease (CHD) and stroke

**STROBE-MR checklist	Pages 79-84**

**References	Page 85**

Appendix Table 1. Antihypertensive drug classes and their corresponding target genes

| Antihypertensive drug classes | Gene | Chromosome | Position (GRCh37/hg19) | Drugs |
| --- | --- | --- | --- | --- |
| Adrenergic neuron blockers | SLC18A2 | 10 | 119,000,625-119,038,941 | Reserpine |
|  | SLC6A2 | 16 | 55,689,900-55,740,104 | Guanethidine |
| Alpha-adrenoceptor antagonists | ADRA1A | 8 | 26,605,667-26,724,795 | Doxazosin, Phenoxybenzamine, Prazosin, Terazosin |
|  | ADRA1D | 20 | 4,201,277-4,229,934 |  |
|  | ADRA1B | 5 | 159,343,489-159,400,019 |  |
|  | ADRA2A | 10 | 112,836,787-112,840,665 | Phenoxybenzamine, Prazosin |
| Angiotensin-converting enzyme inhibitors | ACE | 17 | 61,554,422-61,575,734 | Captopril, Enalapril, Ramipril, Fosinopril, Lisinopril, Moexipril, Perindopril, Quinapril, Trandolapril, Benazepril |
| Angiotensin-II receptor blockers | AGTR1 | 3 | 148,415,690-148,460,790 | Losartan, Irbesartan, Valsartan, Olmesartan, Telmisartan, Eprosartan, Azilsartan medoxomil |
| Beta-adrenoceptor antagonists | ADRB1 | 10 | 115,803,625-115,806,663 | Carteolol, Nadolol, Penbutolol, Pindolol, Propanolol, Sotalol, Timolol, Atenolol, Acebutalol, Betaxolol, Bisoprolol, Carvedilol, Esmolol, Labetolol, Landiolol, Levobunolol, Metaprolol, Nebivlol |
|  | ADRB2 | 5 | 148,206,174-148,208,186 | Carteolol, Nadolol, Penbutolol, Pindolol, Propanolol, Sotalol, Timolol, Atenolol, Acebutalol, Betaxolol, Bisoprolol, Carvedilol, Labetolol, Landiolol, Levobunolol, Metaprolol, Nebivlol |
| Calcium channel blockers | CACNA1C | 12 | 2,162,153-2,807,116 | Amlodipine, Clevidipine, Diltiazem, Felodipine, Isradipine, Levamlodipine, Nicardipine, Nifedipine, Nimodipine, Nislodipine, Verapamil |
|  | CACNA2D1 | 7 | 81,575,760-82,073,272 | Amlodipine, Diltiazem, Felodipine, Isradipine, Nicardipine, Nislodipine |
|  | CACNA1S | 1 | 201,008,640-201,081,554 | Clevidipine, Felodipine, Isradipine, Nifedipine, Nimodipine, Nislodipine |
|  | CACNA1D | 3 | 53,528,638-53,847,760 | Clevidipine, Felodipine, Isradipine, Levamlodipine, Nicardipine, Nifedipine, Nimodipine, Nislodipine |
|  | CACNB2 | 10 | 18,429,353-18,832,486 | Felodipine, Isradipine, Nicardipine, Nifedipine, Nimodipine, Nislodipine |
|  | CACNA1H | 16 | 1,203,106-1,271,768 | Felodipine, Isradipine, Nicardipine, Verapamil |
| Centrally acting anthypertensives | ADARA2A | 10 | 112,836,787-112,840,665 | Clonidine, Methyldopa |
| Loop diuretics | SLC12A1 | 15 | 48,498,499-48,596,275 | Furosemide, Torasemide, Bumetanide, Etacrynic acid |
|  | SLC12A2 | 5 | 127,419,458-127,525,369 | Torasemide, Bumetanide |
| Potassium-sparing diuretics and Aldosterone antagonists | SCNN1A | 12 | 6,456,013-6,486,525 | Amiloride, Triamterene |
|  | SCNN1B | 16 | 23,313,623-23,392,615 |  |
|  | SCNN1G | 16 | 23,194,066-23,228,204 |  |
|  | SCNN1D | 1 | 1,215,816-1,227,405 |  |
|  | NR3C2 | 4 | 148,999,915-149,365,850 | Spironolactone, Eplerenone |
| Renin inhibitors | REN | 1 | 204,123,947-204,135,465 | Aliskiren |
| Thiazide and related diuretics | SLC12A3 | 16 | 56,899,119-56,949,762 | Bendroflumethiazide, Chlorothiazide, Hydrochlorothiazide, Polythiazide, Metolazone, Indapamide |
|  | SLC12A1 | 15 | 48,498,499-48,596,275 | Bendroflumethiazide, Hydroflumethiazide, Methylclothiazide, Chlorthalidone |
|  | KCNMA1 | 10 | 78,629,360-79,397,566 | Bendroflumethiazide, Hydrochlorothiazide, Hydroflumethiazide |
|  | CA1 | 8 | 86,239,837-86,290,342 | Bendroflumethiazide, Chlorothiazide, Hydroflumethiazide, Methylclothiazide, Chlorthalidone |
|  | CA2 | 8 | 86,376,236-86,393,722 | Bendroflumethiazide, Chlorothiazide, Hydroflumethiazide, Methylclothiazide |
|  | CA4 | 17 | 58,227,334-58,256,382 | Bendroflumethiazide, Hydroflumethiazide, Methylclothiazide |
| **Vasodilator antihypertensives** | KCNJ11 | 11 | 17,406,795-17,410,893 | Diazoxide |
|  | DRD5 | 4 | 9,783,258-9,785,633 | Fenoldopam |
|  | DRD1 | 5 | 174,867,039-174,871,185 |  |
|  | AOC3 | 17 | 41,003,216-41,010,141 | Hydralazine |
|  | KCNJ1 | 11 | 128,707,915-128,737,191 | Minoxidil |
|  | PTGS1 | 9 | 125,132,812-125,157,982 |  |
|  | NPR1 | 1 | 153,651,164-153,666,468 | Nitroprusside |

Appendix Table 2. Associations of cis single-nucleotide polymorphisms (SNPs) used as instruments for adrenergic neuron blockers from the systolic blood pressure GWAS by Evangelou et al. ^1^

| Gene | SNP | Chr | Pos | EA | OA | EAF | Beta | SE | P-value | R2 | F |
| --- | --- | --- | --- | --- | --- | --- | --- | --- | --- | --- | --- |
| SLC18A2 | rs2921965 | 10 | 118445251 | T | C | 0.713 | 0.173 | 0.034 | 2.763e-07 | 3.592e-05 | 26.480 |
|  | rs11197813 | 10 | 118523933 | A | G | 0.699 | -0.182 | 0.033 | 3.528e-08 | 4.120e-05 | 30.417 |
| SLC6A2 | rs31079 | 16 | 55359141 | T | C | 0.251 | -0.139 | 0.035 | 6.619e-05 | 2.136e-05 | 15.931 |
|  | rs2397772 | 16 | 55724477 | C | G | 0.617 | -0.121 | 0.031 | 8.688e-05 | 2.063e-05 | 15.385 |
|  | rs12928598 | 16 | 56735782 | A | G | 0.250 | -0.138 | 0.035 | 9.510e-05 | 2.041e-05 | 15.199 |

Abbreviations: SNP, Single Nucleotide Polymorphism; Chr, Chromosome; Pos, Position; EA, Effect Allele; OA, Other Allele; EAF, Effect Allele Frequency; Beta, Effect estimate; SE, Standard Error; R2, proportion of variance explained; F, F-statistic

Appendix Table 3. Associations of cis single-nucleotide polymorphisms (SNPs) used as instruments for alpha-adrenoceptor antagonists from the systolic blood pressure GWAS by Evangelou et al. ^1^

| Gene | SNP | Chr | Pos | EA | OA | EAF | Beta | SE | P-value | R2 | F |
| --- | --- | --- | --- | --- | --- | --- | --- | --- | --- | --- | --- |
| ADRA1A | rs2278143 | 8 | 25634577 | A | G | 0.869 | 0.205 | 0.045 | 5.663e-06 | 2.798e-05 | 20.621 |
|  | rs901177 | 8 | 25867383 | A | T | 0.705 | -0.151 | 0.033 | 6.010e-06 | 2.787e-05 | 20.547 |
|  | rs75523934 | 8 | 25885441 | T | C | 0.975 | 0.402 | 0.099 | 4.949e-05 | 2.234e-05 | 16.480 |
|  | rs7821832 | 8 | 25889446 | T | G | 0.745 | 0.422 | 0.035 | 6.674e-34 | 2.016e-04 | 147.19 |
|  | rs118138958 | 8 | 25899093 | T | G | 0.033 | -0.350 | 0.088 | 6.7100e-05 | 2.157e-05 | 15.900 |
|  | rs74682804 | 8 | 25914683 | C | G | 0.022 | -0.514 | 0.107 | 1.433e-06 | 3.149e-05 | 23.242 |
|  | rs62501779 | 8 | 25932777 | A | G | 0.773 | -0.230 | 0.036 | 2.330e-10 | 5.445e-05 | 40.193 |
|  | rs13439370 | 8 | 25935564 | C | G | 0.723 | -0.184 | 0.036 | 2.586e-07 | 3.596e-05 | 26.507 |
|  | rs7003131 | 8 | 26013917 | A | G | 0.262 | -0.213 | 0.034 | 6.032e-10 | 5.184e-05 | 38.267 |
|  | rs9650417 | 8 | 26029798 | A | G | 0.228 | -0.203 | 0.036 | 2.157e-08 | 4.241e-05 | 31.304 |
|  | rs139890238 | 8 | 26033741 | A | T | 0.891 | 0.197 | 0.050 | 7.866e-05 | 2.110e-05 | 15.555 |
|  | rs77375686 | 8 | 26043622 | A | G | 0.888 | -0.347 | 0.048 | 8.378e-13 | 6.922e-05 | 51.100 |
|  | rs73215676 | 8 | 26115803 | A | G | 0.964 | -0.328 | 0.084 | 9.887e-05 | 2.052e-05 | 15.149 |
|  | rs13261327 | 8 | 26129021 | T | G | 0.986 | -0.528 | 0.135 | 9.215e-05 | 2.082e-05 | 15.291 |
|  | rs9314323 | 8 | 26243136 | A | G | 0.303 | 0.131 | 0.033 | 6.848e-05 | 2.145e-05 | 15.831 |
|  | rs141808794 | 8 | 26266252 | A | G | 0.022 | -0.505 | 0.108 | 3.056e-06 | 2.955e-05 | 21.792 |
|  | rs4629876 | 8 | 26383679 | T | C | 0.309 | -0.144 | 0.033 | 1.028e-05 | 2.638e-05 | 19.473 |
|  | rs45471201 | 8 | 26515138 | T | C | 0.101 | 0.259 | 0.051 | 4.143e-07 | 3.480e-05 | 25.650 |
|  | rs7835853 | 8 | 26662246 | T | C | 0.208 | 0.186 | 0.037 | 6.090e-07 | 3.369e-05 | 24.866 |
|  | rs17296809 | 8 | 26706343 | T | C | 0.944 | -0.332 | 0.066 | 4.426e-07 | 3.463e-05 | 25.489 |
|  | rs2046187 | 8 | 26812434 | A | G | 0.407 | -0.143 | 0.031 | 3.277e-06 | 2.966e-05 | 21.647 |
|  | rs11782789 | 8 | 27266287 | A | T | 0.836 | -0.178 | 0.041 | 1.299e-05 | 2.577e-05 | 19.026 |
|  | rs7465373 | 8 | 27586427 | A | G | 0.554 | 0.118 | 0.030 | 9.454e-05 | 2.062e-05 | 15.218 |
| ADRA1D | rs4815660 | 20 | 4095716 | A | G | 0.662 | -0.175 | 0.032 | 3.478e-08 | 4.072e-05 | 30.371 |
|  | rs1764975 | 20 | 4101290 | A | T | 0.795 | 0.282 | 0.038 | 1.082e-13 | 7.427e-05 | 55.324 |
|  | rs6139317 | 20 | 4104510 | A | T | 0.362 | 0.139 | 0.032 | 1.497e-05 | 2.517e-05 | 18.751 |
|  | rs297738 | 20 | 4413512 | A | G | 0.275 | -0.141 | 0.034 | 3.059e-05 | 2.330e-05 | 17.378 |
| ADRA1B | rs7736883 | 5 | 158343969 | A | G | 0.617 | -0.285 | 0.031 | 3.419e-20 | 1.150e-04 | 84.580 |
|  | rs80066419 | 5 | 158347020 | A | C | 0.075 | -0.337 | 0.058 | 4.446e-09 | 4.678e-05 | 34.39 |
|  | rs62385381 | 5 | 158350706 | T | C | 0.035 | -0.400 | 0.087 | 3.837e-06 | 2.901e-05 | 21.328 |
|  | rs113255182 | 5 | 158355949 | A | G | 0.946 | -0.372 | 0.070 | 1.136e-07 | 3.833e-05 | 28.101 |
|  | rs10077673 | 5 | 158366954 | T | C | 0.963 | 0.353 | 0.081 | 1.378e-05 | 2.600e-05 | 18.888 |
|  | rs147405800 | 5 | 158412406 | T | G | 0.029 | -0.561 | 0.093 | 1.661e-09 | 4.947e-05 | 36.336 |
|  | rs34296591 | 5 | 158444231 | C | G | 0.643 | 0.230 | 0.032 | 3.860e-13 | 7.165e-05 | 52.597 |
|  | rs76688984 | 5 | 158474697 | T | C | 0.104 | 0.223 | 0.050 | 8.043e-06 | 2.713e-05 | 19.945 |
|  | rs7733049 | 5 | 158489476 | C | G | 0.319 | -0.144 | 0.033 | 1.504e-05 | 2.558e-05 | 18.778 |
|  | rs12659540 | 5 | 158522577 | A | C | 0.498 | 0.143 | 0.031 | 3.745e-06 | 2.905e-05 | 21.327 |
|  | rs6895454 | 5 | 158523167 | T | C | 0.077 | -0.331 | 0.058 | 9.935e-09 | 4.471e-05 | 32.868 |
|  | rs2546963 | 5 | 159522488 | T | C | 0.577 | 0.124 | 0.031 | 5.410e-05 | 2.237e-05 | 16.261 |
|  | rs116286399 | 5 | 159576612 | A | G | 0.063 | -0.308 | 0.065 | 2.246e-06 | 3.058e-05 | 22.399 |
| ADRA2A | rs111777102 | 10 | 111965826 | T | C | 0.066 | 0.291 | 0.062 | 2.394e-06 | 3.009e-05 | 22.214 |
|  | rs73356958 | 10 | 112485081 | T | C | 0.178 | 0.174 | 0.040 | 1.105e-05 | 2.611e-05 | 19.276 |
|  | rs2419580 | 10 | 112500340 | T | G | 0.184 | -0.184 | 0.039 | 2.734e-06 | 2.985e-05 | 22.032 |
|  | rs9943402 | 10 | 112610872 | A | G | 0.156 | 0.170 | 0.043 | 6.299e-05 | 2.168e-05 | 15.981 |

Abbreviations: SNP, Single Nucleotide Polymorphism; Chr, Chromosome; Pos, Position; EA, Effect Allele; OA, Other Allele; EAF, Effect Allele Frequency; Beta, Effect estimate; SE, Standard Error; R2, proportion of variance explained; F, F-statistic

Appendix Table 4. Associations of cis single-nucleotide polymorphisms (SNPs) used as instruments for angiotensin-converting enzyme inhibitors from the systolic blood pressure GWAS by Evangelou et al. ^1^

| Gene | SNP | Chr | Pos | EA | OA | EAF | Beta | SE | P-value | R2 | F |
| --- | --- | --- | --- | --- | --- | --- | --- | --- | --- | --- | --- |
| ACE | rs113301871 | 17 | 60555375 | T | C | 0.358 | 0.168 | 0.033 | 2.916e-07 | 3.573e-05 | 26.231 |
|  | rs56288724 | 17 | 60767135 | A | G | 0.583 | -0.218 | 0.031 | 2.011e-12 | 6.628e-05 | 49.362 |
|  | rs2460288 | 17 | 60792800 | T | C | 0.11 | -0.198 | 0.050 | 6.728e-05 | 2.163e-05 | 15.872 |
|  | rs62076622 | 17 | 61090958 | A | G | 0.801 | 0.236 | 0.038 | 3.785e-10 | 5.267e-05 | 39.287 |
|  | rs9899012 | 17 | 61545486 | A | G | 0.061 | -0.304 | 0.068 | 8.480e-06 | 2.757e-05 | 19.850 |
|  | rs8077276 | 17 | 61547562 | A | G | 0.62 | -0.295 | 0.031 | 5.145e-21 | 1.186e-04 | 88.204 |
|  | rs8066276 | 17 | 61589265 | T | C | 0.408 | -0.128 | 0.031 | 3.649e-05 | 2.305e-05 | 16.992 |
|  | rs79480822 | 17 | 61654225 | T | C | 0.053 | 0.323 | 0.075 | 1.654e-05 | 2.579e-05 | 18.559 |
|  | rs11655833 | 17 | 62083212 | A | T | 0.097 | -0.312 | 0.056 | 2.863e-08 | 4.200e-05 | 30.750 |
|  | rs8075518 | 17 | 62212836 | T | C | 0.601 | 0.177 | 0.032 | 3.657e-08 | 4.068e-05 | 30.301 |
|  | rs6504213 | 17 | 62381714 | T | C | 0.418 | -0.298 | 0.031 | 1.248e-21 | 1.226e-04 | 91.349 |
|  | rs11655644 | 17 | 62393621 | T | C | 0.063 | -0.310 | 0.069 | 7.432e-06 | 2.730e-05 | 20.081 |
|  | rs41548920 | 17 | 62493390 | A | G | 0.979 | 0.465 | 0.110 | 2.408e-05 | 2.448e-05 | 17.853 |
|  | rs1991401 | 17 | 62502435 | A | G | 0.678 | 0.251 | 0.033 | 2.921e-14 | 7.913e-05 | 57.686 |

Abbreviations: SNP, Single Nucleotide Polymorphism; Chr, Chromosome; Pos, Position; EA, Effect Allele; OA, Other Allele; EAF, Effect Allele Frequency; Beta, Effect estimate; SE, Standard Error; R2, proportion of variance explained; F, F-statistic

Appendix Table 5. Associations of cis single-nucleotide polymorphisms (SNPs) used as instruments for angiotensin-II receptor blockers from the systolic blood pressure GWAS by Evangelou et al. ^1^

| Gene | SNP | Chr | Pos | EA | OA | EAF | Beta | SE | P-value | R2 | F |
| --- | --- | --- | --- | --- | --- | --- | --- | --- | --- | --- | --- |
| AGTR1 | rs11707568 | 3 | 148511662 | A | C | 0.744 | -0.147 | 0.036 | 5.580e-05 | 2.206e-05 | 16.264 |
|  | rs893982 | 3 | 148616274 | C | G | 0.400 | -0.159 | 0.031 | 2.210e-07 | 3.644e-05 | 26.824 |
|  | rs6780836 | 3 | 148672237 | T | C | 0.462 | 0.122 | 0.030 | 5.565e-05 | 2.196e-05 | 16.211 |
|  | rs17783804 | 3 | 148673850 | T | C | 0.790 | -0.148 | 0.038 | 7.970e-05 | 2.116e-05 | 15.576 |

Abbreviations: SNP, Single Nucleotide Polymorphism; Chr, Chromosome; Pos, Position; EA, Effect Allele; OA, Other Allele; EAF, Effect Allele Frequency; Beta, Effect estimate; SE, Standard Error; R2, proportion of variance explained; F, F-statistic

Appendix Table 6. Associations of cis single-nucleotide polymorphisms (SNPs) used as instruments for beta-adrenoceptor antagonists from the systolic blood pressure GWAS by Evangelou et al. ^1^

| Gene | SNP | Chr | Pos | EA | OA | EAF | Beta | SE | P-value | R2 | F |
| --- | --- | --- | --- | --- | --- | --- | --- | --- | --- | --- | --- |
| ADRB1 | rs35519679 | 10 | 114818754 | A | G | 0.252 | 0.254 | 0.036 | 1.150e-12 | 6.864e-05 | 50.457 |
|  | rs61872795 | 10 | 114835674 | T | C | 0.277 | 0.228 | 0.034 | 2.676e-11 | 6.005e-05 | 44.327 |
|  | rs749596 | 10 | 114847905 | A | T | 0.210 | -0.209 | 0.038 | 5.092e-08 | 4.025e-05 | 29.623 |
|  | rs112802057 | 10 | 114892604 | A | G | 0.945 | 0.272 | 0.069 | 8.532e-05 | 2.109e-05 | 15.438 |
|  | rs10885421 | 10 | 114916586 | T | G | 0.599 | 0.155 | 0.032 | 8.650e-07 | 3.284e-05 | 24.213 |
|  | rs117992316 | 10 | 115493027 | T | G | 0.942 | 0.263 | 0.066 | 7.765e-05 | 2.119e-05 | 15.617 |
|  | rs75228369 | 10 | 115684914 | T | C | 0.049 | 0.290 | 0.074 | 9.184e-05 | 2.073e-05 | 15.305 |
|  | rs11817866 | 10 | 115690539 | A | T | 0.568 | 0.173 | 0.031 | 1.878e-08 | 4.287e-05 | 31.645 |
|  | rs7894582 | 10 | 115704458 | A | C | 0.047 | -0.302 | 0.073 | 3.565e-05 | 2.313e-05 | 17.078 |
|  | rs11196549 | 10 | 115707298 | A | G | 0.043 | 0.688 | 0.078 | 1.580e-18 | 1.044e-04 | 77.099 |
|  | rs460718 | 10 | 115721364 | A | G | 0.327 | -0.276 | 0.032 | 1.358e-17 | 9.858e-05 | 72.775 |
|  | rs12767891 | 10 | 115748333 | T | G | 0.494 | -0.16 | 0.031 | 2.155e-07 | 3.637e-05 | 26.851 |
|  | rs678669 | 10 | 115764032 | T | C | 0.609 | -0.138 | 0.031 | 1.017e-05 | 2.670e-05 | 19.479 |
|  | rs11196597 | 10 | 115788094 | A | G | 0.133 | 0.286 | 0.046 | 4.227e-10 | 5.282e-05 | 38.940 |
|  | rs17875473 | 10 | 115800294 | T | C | 0.087 | 0.328 | 0.055 | 2.658e-09 | 4.792e-05 | 35.372 |
|  | rs1801253 | 10 | 115805056 | C | G | 0.734 | 0.463 | 0.034 | 2.841e-41 | 2.449e-04 | 180.84 |
|  | rs75098615 | 10 | 115805333 | A | C | 0.022 | -0.579 | 0.109 | 1.119e-07 | 3.844e-05 | 28.145 |
|  | rs68122733 | 10 | 115831533 | A | G | 0.828 | 0.334 | 0.041 | 1.688e-16 | 9.184e-05 | 67.798 |
|  | rs143854972 | 10 | 115843445 | A | G | 0.058 | 0.431 | 0.066 | 8.894e-11 | 5.697e-05 | 42.054 |
|  | rs11196627 | 10 | 115845762 | T | C | 0.608 | -0.156 | 0.032 | 9.387e-07 | 3.269e-05 | 24.065 |
|  | rs12219192 | 10 | 116000453 | T | C | 0.160 | 0.183 | 0.045 | 4.734e-05 | 2.264e-05 | 16.519 |
|  | rs4751648 | 10 | 116132246 | T | C | 0.560 | 0.152 | 0.030 | 6.674e-07 | 3.365e-05 | 24.771 |
|  | rs35221086 | 10 | 116647316 | A | T | 0.531 | -0.146 | 0.030 | 1.372e-06 | 3.158e-05 | 23.313 |
| ADRB2 | rs12717974 | 5 | 147829177 | C | G | 0.724 | 0.180 | 0.034 | 1.315e-07 | 3.787e-05 | 27.802 |
|  | rs9716164 | 5 | 147884668 | T | C | 0.418 | 0.180 | 0.030 | 3.401e-09 | 4.759e-05 | 34.984 |
|  | rs56373125 | 5 | 147889702 | A | G | 0.940 | 0.283 | 0.065 | 1.262e-05 | 2.595e-05 | 19.078 |
|  | rs2400643 | 5 | 148114897 | A | G | 0.770 | 0.144 | 0.036 | 6.968e-05 | 2.177e-05 | 15.823 |
|  | rs888956 | 5 | 148120217 | A | C | 0.771 | -0.148 | 0.037 | 5.976e-05 | 2.188e-05 | 16.087 |
|  | rs11959615 | 5 | 148201316 | A | T | 0.554 | 0.130 | 0.030 | 1.808e-05 | 2.499e-05 | 18.371 |
|  | rs12055246 | 5 | 148335178 | A | C | 0.194 | -0.158 | 0.039 | 5.189e-05 | 2.230e-05 | 16.371 |
|  | rs78909293 | 5 | 148335250 | T | C | 0.956 | 0.377 | 0.075 | 5.146e-07 | 3.433e-05 | 25.240 |
|  | rs17640858 | 5 | 148346260 | A | C | 0.369 | 0.201 | 0.032 | 3.295e-10 | 5.362e-05 | 39.365 |
|  | rs994446 | 5 | 148348395 | A | G | 0.209 | -0.347 | 0.037 | 1.874e-20 | 1.168e-04 | 85.884 |
|  | rs111345025 | 5 | 148465229 | T | C | 0.027 | -0.474 | 0.100 | 2.048e-06 | 3.130e-05 | 22.548 |
|  | rs4705316 | 5 | 148498799 | A | G | 0.76 | -0.223 | 0.035 | 3.227e-10 | 5.362e-05 | 39.424 |
|  | rs114464628 | 5 | 148533724 | A | G | 0.039 | 0.363 | 0.081 | 7.123e-06 | 2.767e-05 | 20.144 |
|  | rs10051277 | 5 | 148547201 | C | G | 0.067 | -0.378 | 0.063 | 2.028e-09 | 4.894e-05 | 35.981 |
|  | rs11749868 | 5 | 148600693 | A | G | 0.069 | -0.294 | 0.066 | 7.072e-06 | 2.820e-05 | 20.202 |
|  | rs12152968 | 5 | 148622345 | T | C | 0.488 | 0.135 | 0.030 | 7.878e-06 | 2.722e-05 | 20.012 |

Abbreviations: SNP, Single Nucleotide Polymorphism; Chr, Chromosome; Pos, Position; EA, Effect Allele; OA, Other Allele; EAF, Effect Allele Frequency; Beta, Effect estimate; SE, Standard Error; R2, proportion of variance explained; F, F-statistic

Appendix Table 7. Associations of cis single-nucleotide polymorphisms (SNPs) used as instruments for calcium channel blockers from the systolic blood pressure GWAS by Evangelou et al. ^1^

| Gene | SNP | Chr | Pos | EA | OA | EAF | Beta | SE | P-value | R2 | F |
| --- | --- | --- | --- | --- | --- | --- | --- | --- | --- | --- | --- |
| CACNA1C | rs143459027 | 12 | 2212867 | T | C | 0.030 | 0.437 | 0.095 | 4.363e-06 | 2.832e-05 | 21.110 |
|  | rs2239046 | 12 | 2434419 | A | G | 0.682 | 0.208 | 0.032 | 9.577e-11 | 5.605e-05 | 41.807 |
|  | rs714277 | 12 | 2514270 | T | C | 0.283 | 0.199 | 0.033 | 2.378e-09 | 4.769e-05 | 35.569 |
|  | rs4765961 | 12 | 2668472 | T | C | 0.832 | 0.205 | 0.040 | 3.740e-07 | 3.459e-05 | 25.798 |
|  | rs11612237 | 12 | 3031161 | A | C | 0.980 | -0.482 | 0.114 | 2.545e-05 | 2.389e-05 | 17.730 |
| CACNA1D | rs41292372 | 3 | 52541937 | T | C | 0.034 | -0.360 | 0.092 | 8.913e-05 | 2.114e-05 | 15.337 |
|  | rs10433615 | 3 | 52638482 | T | C | 0.394 | 0.169 | 0.031 | 5.092e-08 | 4.017e-05 | 29.615 |
|  | rs114796738 | 3 | 52757046 | T | C | 0.051 | -0.320 | 0.074 | 1.552e-05 | 2.567e-05 | 18.653 |
|  | rs74380125 | 3 | 53077621 | T | C | 0.976 | -0.412 | 0.105 | 8.301e-05 | 2.099e-05 | 15.477 |
|  | rs7615099 | 3 | 53143901 | A | G | 0.667 | 0.189 | 0.032 | 3.903e-09 | 4.707e-05 | 34.703 |
|  | rs79270291 | 3 | 53173018 | A | G | 0.987 | -0.657 | 0.147 | 7.395e-06 | 2.728e-05 | 20.078 |
|  | rs62254274 | 3 | 53180805 | A | G | 0.868 | 0.208 | 0.045 | 3.833e-06 | 2.894e-05 | 21.365 |
|  | rs147254549 | 3 | 53290198 | C | G | 0.064 | -0.247 | 0.062 | 7.857e-05 | 2.112e-05 | 15.593 |
|  | rs114718455 | 3 | 53464055 | A | G | 0.966 | -0.510 | 0.090 | 1.718e-08 | 4.349e-05 | 31.782 |
|  | rs312476 | 3 | 53514416 | T | G | 0.248 | 0.141 | 0.035 | 6.681e-05 | 2.158e-05 | 15.910 |
|  | rs3821843 | 3 | 53558012 | A | G | 0.681 | 0.337 | 0.034 | 6.559e-24 | 1.377e-04 | 101.38 |
|  | rs114987861 | 3 | 53605712 | A | G | 0.028 | 0.529 | 0.096 | 3.360e-08 | 4.135e-05 | 30.480 |
|  | rs113210396 | 3 | 53612327 | T | G | 0.045 | -0.434 | 0.077 | 1.760e-08 | 4.305e-05 | 31.739 |
|  | rs116535016 | 3 | 53645516 | A | G | 0.022 | 0.501 | 0.107 | 3.013e-06 | 2.956e-05 | 21.804 |
|  | rs7340705 | 3 | 53734443 | T | C | 0.673 | -0.242 | 0.032 | 4.873e-14 | 7.683e-05 | 56.717 |
|  | rs3774531 | 3 | 53737575 | T | C | 0.490 | 0.132 | 0.030 | 1.196e-05 | 2.589e-05 | 19.115 |
|  | rs2612015 | 3 | 53742331 | T | G | 0.631 | 0.125 | 0.032 | 8.029e-05 | 2.109e-05 | 15.550 |
|  | rs12485510 | 3 | 53850209 | T | C | 0.540 | 0.152 | 0.030 | 5.948e-07 | 3.387e-05 | 24.967 |
|  | rs62250937 | 3 | 53870318 | T | C | 0.911 | 0.324 | 0.056 | 7.279e-09 | 4.542e-05 | 33.433 |
|  | rs60978131 | 3 | 53982904 | T | C | 0.015 | 0.577 | 0.144 | 5.709e-05 | 2.213e-05 | 16.190 |
|  | rs10865981 | 3 | 54003534 | A | C | 0.909 | 0.256 | 0.053 | 1.448e-06 | 3.149e-05 | 23.243 |
| CACNA1H | rs4984906 | 16 | 709001 | A | C | 0.366 | 0.160 | 0.032 | 4.489e-07 | 3.457e-05 | 25.410 |
|  | rs62013846 | 16 | 1063999 | C | G | 0.042 | -0.357 | 0.081 | 9.448e-06 | 2.718e-05 | 19.640 |
|  | rs11641374 | 16 | 1347717 | A | C | 0.600 | -0.194 | 0.031 | 3.260e-10 | 5.331e-05 | 39.539 |
|  | rs4984836 | 16 | 1481797 | A | G | 0.119 | 0.274 | 0.048 | 7.782e-09 | 4.480e-05 | 33.323 |
|  | rs2092387 | 16 | 1534320 | A | G | 0.068 | 0.265 | 0.063 | 2.399e-05 | 2.400e-05 | 17.850 |
|  | rs2235641 | 16 | 1608177 | A | G | 0.744 | -0.137 | 0.034 | 7.035e-05 | 2.147e-05 | 15.838 |
|  | rs344367 | 16 | 1976170 | T | C | 0.688 | -0.172 | 0.034 | 5.049e-07 | 3.430e-05 | 25.176 |
|  | rs1860236 | 16 | 2023372 | A | C | 0.847 | -0.2 | 0.044 | 6.104e-06 | 2.837e-05 | 20.485 |
|  | rs113097793 | 16 | 2037831 | A | G | 0.192 | -0.218 | 0.039 | 1.688e-08 | 4.283e-05 | 31.808 |
|  | rs55662859 | 16 | 2052806 | A | G | 0.097 | 0.309 | 0.054 | 1.150e-08 | 4.444e-05 | 32.559 |
|  | rs28590346 | 16 | 2080653 | A | T | 0.660 | -0.271 | 0.033 | 3.365e-16 | 9.188e-05 | 66.580 |
|  | rs34165865 | 16 | 2087785 | T | C | 0.800 | 0.227 | 0.039 | 6.123e-09 | 4.607e-05 | 33.848 |
|  | rs139982960 | 16 | 2115033 | T | C | 0.930 | -0.373 | 0.065 | 1.083e-08 | 4.561e-05 | 32.711 |
|  | rs149048921 | 16 | 2116313 | A | G | 0.022 | 0.711 | 0.113 | 3.533e-10 | 5.522e-05 | 39.369 |
|  | rs67633992 | 16 | 2219751 | A | T | 0.937 | -0.346 | 0.065 | 9.884e-08 | 3.920e-05 | 28.401 |
| CACNA1S | rs903676 | 1 | 201754720 | A | G | 0.213 | -0.156 | 0.037 | 2.553e-05 | 2.405e-05 | 17.754 |
| CACNB2 | rs508196 | 10 | 18263873 | T | C | 0.431 | -0.163 | 0.031 | 1.148e-07 | 3.819e-05 | 28.111 |
|  | rs2497755 | 10 | 18282667 | A | T | 0.105 | 0.303 | 0.049 | 7.718e-10 | 5.117e-05 | 37.774 |
|  | rs7893618 | 10 | 18299520 | A | G | 0.432 | 0.142 | 0.033 | 1.744e-05 | 2.504e-05 | 18.456 |
|  | rs1891392 | 10 | 18336421 | T | C | 0.287 | 0.228 | 0.033 | 9.020e-12 | 6.290e-05 | 46.436 |
|  | rs2488152 | 10 | 18355456 | A | G | 0.895 | -0.377 | 0.050 | 2.543e-14 | 7.853e-05 | 57.975 |
|  | rs76304369 | 10 | 18359213 | T | C | 0.013 | 0.729 | 0.138 | 1.186e-07 | 3.817e-05 | 28.053 |
|  | rs75699707 | 10 | 18359294 | A | G | 0.026 | -0.576 | 0.103 | 2.103e-08 | 4.301e-05 | 31.406 |
|  | rs78351253 | 10 | 18361384 | A | G | 0.960 | 0.352 | 0.080 | 1.213e-05 | 2.595e-05 | 19.153 |
|  | rs7079408 | 10 | 18377343 | A | G | 0.693 | 0.177 | 0.033 | 7.577e-08 | 3.912e-05 | 28.878 |
|  | rs78607408 | 10 | 18386117 | T | C | 0.954 | 0.332 | 0.075 | 1.031e-05 | 2.632e-05 | 19.433 |
|  | rs74688261 | 10 | 18412294 | A | G | 0.925 | -0.269 | 0.058 | 3.108e-06 | 2.947e-05 | 21.751 |
|  | rs1888693 | 10 | 18440444 | A | G | 0.345 | 0.386 | 0.032 | 4.691e-34 | 2.012e-04 | 148.12 |
|  | rs16916914 | 10 | 18457722 | T | C | 0.963 | -0.564 | 0.081 | 2.719e-12 | 6.630e-05 | 48.896 |
|  | rs7076319 | 10 | 18459450 | A | G | 0.734 | -0.321 | 0.034 | 5.073e-21 | 1.202e-04 | 88.614 |
|  | rs17605594 | 10 | 18471064 | T | C | 0.055 | -0.290 | 0.074 | 9.385e-05 | 2.071e-05 | 15.264 |
|  | rs61278674 | 10 | 18481737 | A | G | 0.906 | -0.330 | 0.054 | 1.032e-09 | 5.060e-05 | 37.300 |
|  | rs11012983 | 10 | 18490601 | A | T | 0.892 | -0.221 | 0.049 | 6.802e-06 | 2.753e-05 | 20.213 |
|  | rs1779209 | 10 | 18514561 | T | C | 0.288 | 0.274 | 0.034 | 4.228e-16 | 9.089e-05 | 66.306 |
|  | rs10828399 | 10 | 18553968 | A | G | 0.522 | -0.195 | 0.030 | 1.102e-10 | 5.63e-05 | 41.564 |
|  | rs9633646 | 10 | 18564922 | A | G | 0.595 | -0.134 | 0.031 | 1.460e-05 | 2.545e-05 | 18.787 |
|  | rs79666207 | 10 | 18583840 | T | C | 0.982 | 0.628 | 0.122 | 2.613e-07 | 3.596e-05 | 26.515 |
|  | rs10828452 | 10 | 18592450 | A | T | 0.793 | 0.305 | 0.039 | 4.198e-15 | 8.360e-05 | 61.630 |
|  | rs74494383 | 10 | 18626340 | A | C | 0.019 | 0.539 | 0.118 | 5.27e-06 | 2.844e-05 | 20.724 |
|  | rs10828542 | 10 | 18627285 | A | G | 0.614 | 0.182 | 0.031 | 5.178e-09 | 4.624e-05 | 34.134 |
|  | rs12780039 | 10 | 18678987 | C | G | 0.121 | 0.285 | 0.047 | 1.263e-09 | 4.988e-05 | 36.822 |
|  | rs2148184 | 10 | 18680963 | T | C | 0.868 | -0.196 | 0.045 | 1.552e-05 | 2.534e-05 | 18.707 |
|  | rs61839258 | 10 | 18689638 | T | G | 0.173 | 0.160 | 0.040 | 6.426e-05 | 2.165e-05 | 15.960 |
|  | rs112133583 | 10 | 18695681 | T | C | 0.030 | -0.555 | 0.097 | 1.181e-08 | 4.407e-05 | 32.489 |
|  | rs79615672 | 10 | 18700784 | T | C | 0.024 | -0.480 | 0.106 | 5.832e-06 | 2.815e-05 | 20.531 |
|  | rs11014170 | 10 | 18710991 | A | G | 0.021 | -0.670 | 0.115 | 5.613e-09 | 4.637e-05 | 33.953 |
|  | rs7923191 | 10 | 18727901 | A | G | 0.792 | -0.369 | 0.038 | 1.095e-22 | 1.307e-04 | 96.311 |
|  | rs12258967 | 10 | 18727959 | C | G | 0.705 | 0.633 | 0.034 | 1.083e-78 | 4.779e-04 | 352.48 |
|  | rs72786098 | 10 | 18729855 | A | G | 0.032 | -0.503 | 0.088 | 1.180e-08 | 4.408e-05 | 32.489 |
|  | rs71497251 | 10 | 18781265 | A | C | 0.188 | 0.203 | 0.040 | 2.894e-07 | 3.572e-05 | 26.330 |
|  | rs7080574 | 10 | 18788342 | C | G | 0.414 | -0.160 | 0.034 | 1.879e-06 | 3.079e-05 | 22.697 |
|  | rs4748474 | 10 | 18790727 | A | G | 0.521 | 0.195 | 0.030 | 1.614e-10 | 5.614e-05 | 40.977 |
|  | rs11015633 | 10 | 18979469 | A | G | 0.575 | 0.143 | 0.031 | 3.342e-06 | 2.931e-05 | 21.636 |
|  | rs11015818 | 10 | 19000055 | T | C | 0.607 | 0.133 | 0.032 | 3.858e-05 | 2.300e-05 | 16.952 |
|  | rs180849797 | 10 | 19340761 | A | G | 0.016 | 0.543 | 0.137 | 7.647e-05 | 2.134e-05 | 15.646 |

Abbreviations: SNP, Single Nucleotide Polymorphism; Chr, Chromosome; Pos, Position; EA, Effect Allele; OA, Other Allele; EAF, Effect Allele Frequency; Beta, Effect estimate; SE, Standard Error; R2, proportion of variance explained; F, F-statistic

Appendix Table 8. Associations of cis single-nucleotide polymorphisms (SNPs) used as instruments for centrally acting antihypertensives from the systolic blood pressure GWAS by Evangelou et al. ^1^

| Gene | SNP | Chr | Pos | EA | OA | EAF | Beta | SE | P-value | R2 | F |
| --- | --- | --- | --- | --- | --- | --- | --- | --- | --- | --- | --- |
| ADRA2A | rs111777102 | 10 | 111965826 | T | C | 0.066 | 0.291 | 0.062 | 2.394e-06 | 3.009e-05 | 22.214 |
|  | rs73356958 | 10 | 112485081 | T | C | 0.178 | 0.174 | 0.040 | 1.105e-05 | 2.611e-05 | 19.276 |
|  | rs2419580 | 10 | 112500340 | T | G | 0.184 | -0.184 | 0.039 | 2.734e-06 | 2.985e-05 | 22.032 |
|  | rs9943402 | 10 | 112610872 | A | G | 0.156 | 0.170 | 0.043 | 6.299e-05 | 2.168e-05 | 15.981 |

Abbreviations: SNP, Single Nucleotide Polymorphism; Chr, Chromosome; Pos, Position; EA, Effect Allele; OA, Other Allele; EAF, Effect Allele Frequency; Beta, Effect estimate; SE, Standard Error; R2, proportion of variance explained; F, F-statistic

Appendix Table 9. Associations of cis single-nucleotide polymorphisms (SNPs) used as instruments for loop diuretics from the systolic blood pressure GWAS by Evangelou et al. ^1^

| Gene | SNP | Chr | Pos | EA | OA | EAF | Beta | SE | P-value | R2 | F |
| --- | --- | --- | --- | --- | --- | --- | --- | --- | --- | --- | --- |
| SLC12A2 | rs61368741 | 5 | 126621087 | T | C | 0.499 | -0.122 | 0.030 | 5.265e-05 | 2.222e-05 | 16.401 |
|  | rs711364 | 5 | 127000694 | C | G | 0.248 | 0.142 | 0.035 | 4.604e-05 | 2.246e-05 | 16.578 |
|  | rs13356360 | 5 | 127247719 | T | C | 0.961 | -0.348 | 0.087 | 5.963e-05 | 2.212e-05 | 16.102 |
|  | rs6860245 | 5 | 127367998 | C | G | 0.244 | -0.211 | 0.035 | 2.098e-09 | 4.849e-05 | 35.796 |
|  | rs17676242 | 5 | 127589648 | A | G | 0.092 | 0.416 | 0.053 | 6.622e-15 | 8.259e-05 | 60.799 |
|  | rs78736765 | 5 | 127693997 | T | C | 0.151 | -0.212 | 0.043 | 7.085e-07 | 3.344e-05 | 24.650 |
|  | rs80097588 | 5 | 127729949 | A | G | 0.939 | -0.364 | 0.065 | 1.978e-08 | 4.273e-05 | 31.502 |
|  | rs331067 | 5 | 127792031 | A | G | 0.114 | -0.221 | 0.048 | 4.315e-06 | 2.858e-05 | 21.099 |
|  | rs10478813 | 5 | 127831265 | C | G | 0.060 | 0.347 | 0.064 | 5.197e-08 | 4.016e-05 | 29.649 |
|  | rs6892983 | 5 | 127845030 | A | C | 0.402 | 0.343 | 0.031 | 7.113e-29 | 1.690e-04 | 124.61 |
|  | rs62390671 | 5 | 127873094 | A | G | 0.047 | 0.295 | 0.071 | 3.604e-05 | 2.314e-05 | 17.084 |
|  | rs116561167 | 5 | 127899008 | C | G | 0.969 | -0.362 | 0.091 | 6.787e-05 | 2.152e-05 | 15.885 |
|  | rs247095 | 5 | 128093756 | A | G | 0.740 | 0.196 | 0.035 | 1.676e-08 | 4.332e-05 | 31.839 |
|  | rs192299 | 5 | 128413093 | T | C | 0.749 | -0.155 | 0.035 | 9.998e-06 | 2.650e-05 | 19.562 |
|  | rs3798119 | 5 | 128446836 | A | G | 0.198 | -0.152 | 0.038 | 5.783e-05 | 2.188e-05 | 16.148 |
| SLC12A1 | rs4774498 | 15 | 47873605 | A | T | 0.666 | -0.131 | 0.032 | 3.955e-05 | 2.276e-05 | 16.890 |
|  | rs4775769 | 15 | 48939888 | T | G | 0.094 | -0.416 | 0.052 | 7.757e-16 | 8.689e-05 | 64.807 |

Abbreviations: SNP, Single Nucleotide Polymorphism; Chr, Chromosome; Pos, Position; EA, Effect Allele; OA, Other Allele; EAF, Effect Allele Frequency; Beta, Effect estimate; SE, Standard Error; R2, proportion of variance explained; F, F-statistic

Appendix Table 10. Associations of cis single-nucleotide polymorphisms (SNPs) used as instruments for potassium-sparing diuretics and aldosterone antagonists from the systolic blood pressure GWAS by Evangelou et al. ^1^

| Gene | SNP | Chr | Pos | EA | OA | EAF | Beta | SE | P-value | R2 | F |
| --- | --- | --- | --- | --- | --- | --- | --- | --- | --- | --- | --- |
| SCNN1A | rs12369621 | 12 | 5457315 | T | C | 0.089 | -0.251 | 0.053 | 2.299e-06 | 3.000e-05 | 22.344 |
|  | rs4764621 | 12 | 6879279 | A | G | 0.585 | -0.127 | 0.031 | 4.146e-05 | 2.246e-05 | 16.755 |
| SCNN1BandG | rs75964872 | 16 | 23847575 | A | C | 0.195 | -0.162 | 0.038 | 1.901e-05 | 2.452e-05 | 18.287 |
|  | rs8047317 | 16 | 23882044 | A | G | 0.426 | -0.120 | 0.030 | 7.791e-05 | 2.095e-05 | 15.582 |
| SCNN1D | rs78548753 | 1 | 1163964 | A | G | 0.030 | -0.370 | 0.095 | 9.478e-05 | 2.146e-05 | 15.232 |
|  | rs307359 | 1 | 1280014 | A | G | 0.069 | -0.379 | 0.065 | 5.175e-09 | 5.074e-05 | 34.136 |
|  | rs7796 | 1 | 1684169 | C | G | 0.511 | 0.338 | 0.031 | 4.997e-27 | 1.599e-04 | 116.21 |
|  | rs3128334 | 1 | 2038662 | A | G | 0.633 | 0.207 | 0.032 | 1.408e-10 | 5.779e-05 | 41.071 |
|  | rs6656309 | 1 | 2149138 | C | G | 0.761 | -0.215 | 0.036 | 2.966e-09 | 4.800e-05 | 35.211 |
|  | rs6673129 | 1 | 2202774 | T | C | 0.162 | 0.193 | 0.043 | 8.095e-06 | 2.772e-05 | 19.908 |
|  | rs12743493 | 1 | 2224836 | A | G | 0.375 | -0.158 | 0.032 | 9.889e-07 | 3.332e-05 | 23.931 |
| NR3C2 | rs13143677 | 4 | 148419040 | A | G | 0.293 | 0.224 | 0.033 | 1.821e-11 | 6.113e-05 | 45.128 |
|  | rs12501094 | 4 | 148520824 | T | C | 0.294 | -0.183 | 0.036 | 5.190e-07 | 3.459e-05 | 25.220 |
|  | rs4835570 | 4 | 150202672 | T | C | 0.201 | 0.153 | 0.038 | 5.399e-05 | 2.211e-05 | 16.318 |

Abbreviations: SNP, Single Nucleotide Polymorphism; Chr, Chromosome; Pos, Position; EA, Effect Allele; OA, Other Allele; EAF, Effect Allele Frequency; Beta, Effect estimate; SE, Standard Error; R2, proportion of variance explained; F, F-statistic

Appendix Table 11. Associations of cis single-nucleotide polymorphisms (SNPs) used as instruments for renin inhibitors from the systolic blood pressure GWAS by Evangelou et al. ^1^

| Gene | SNP | Chr | Pos | EA | OA | EAF | Beta | SE | P-value | R2 | F |
| --- | --- | --- | --- | --- | --- | --- | --- | --- | --- | --- | --- |
| REN | rs3753473 | 1 | 203131285 | A | G | 0.06 | -0.307 | 0.064 | 1.651e-06 | 3.149e-05 | 22.983 |
|  | rs6664234 | 1 | 203693374 | A | G | 0.815 | 0.157 | 0.039 | 5.167e-05 | 2.221e-05 | 16.394 |
|  | rs16852778 | 1 | 204051441 | T | C | 0.157 | 0.229 | 0.043 | 7.699e-08 | 3.910e-05 | 28.821 |
|  | rs61825748 | 1 | 204092502 | T | G | 0.050 | -0.284 | 0.072 | 7.333e-05 | 2.144e-05 | 15.722 |
|  | rs141660869 | 1 | 204113987 | A | C | 0.970 | 0.487 | 0.092 | 1.210e-07 | 3.798e-05 | 28.036 |
|  | rs11806216 | 1 | 204833446 | A | G | 0.067 | -0.241 | 0.062 | 9.122e-05 | 2.074e-05 | 15.307 |

Abbreviations: SNP, Single Nucleotide Polymorphism; Chr, Chromosome; Pos, Position; EA, Effect Allele; OA, Other Allele; EAF, Effect Allele Frequency; Beta, Effect estimate; SE, Standard Error; R2, proportion of variance explained; F, F-statistic

Appendix Table 12. Associations of cis single-nucleotide polymorphisms (SNPs) used as instruments for thiazide and related diuretics from the systolic blood pressure GWAS by Evangelou et al. ^1^

| Gene | SNP | Chr | Pos | EA | OA | EAF | Beta | SE | P-value | R2 | F |
| --- | --- | --- | --- | --- | --- | --- | --- | --- | --- | --- | --- |
| KCNMA1 | rs2579763 | 10 | 78317419 | T | C | 0.961 | 0.334 | 0.083 | 5.208e-05 | 2.243e-05 | 16.360 |
| SLC12A1 | rs4774498 | 15 | 47873605 | A | T | 0.666 | -0.131 | 0.032 | 3.955e-05 | 2.276e-05 | 16.890 |
|  | rs4775769 | 15 | 48939888 | T | G | 0.094 | -0.416 | 0.052 | 7.757e-16 | 8.689e-05 | 64.807 |
| SLC12A3 | rs4783956 | 16 | 56766831 | T | C | 0.287 | -0.177 | 0.033 | 8.983e-08 | 3.830e-05 | 28.563 |
|  | rs55901950 | 16 | 56920146 | A | G | 0.085 | -0.243 | 0.055 | 1.038e-05 | 2.601e-05 | 19.401 |
|  | rs9925265 | 16 | 56950210 | A | G | 0.428 | 0.121 | 0.030 | 7.169e-05 | 2.120e-05 | 15.764 |
| CA4 | rs76676891 | 17 | 57253639 | T | G | 0.986 | 0.553 | 0.137 | 5.762e-05 | 2.227e-05 | 16.175 |
|  | rs59549190 | 17 | 57384444 | A | G | 0.336 | -0.217 | 0.033 | 6.018e-11 | 5.735e-05 | 42.721 |
|  | rs1451506 | 17 | 57407019 | A | G | 0.116 | 0.207 | 0.049 | 2.247e-05 | 2.412e-05 | 17.937 |
|  | rs1292041 | 17 | 57938041 | A | G | 0.483 | -0.163 | 0.031 | 1.717e-07 | 3.664e-05 | 27.294 |
|  | rs78638044 | 17 | 57974476 | A | G | 0.122 | 0.280 | 0.049 | 1.161e-08 | 4.394e-05 | 32.504 |
|  | rs1197070 | 17 | 58634810 | A | T | 0.178 | 0.172 | 0.042 | 3.690e-05 | 2.356e-05 | 16.993 |
|  | rs9652858 | 17 | 59087808 | A | G | 0.181 | 0.255 | 0.040 | 1.527e-10 | 5.485e-05 | 40.909 |

Abbreviations: SNP, Single Nucleotide Polymorphism; Chr, Chromosome; Pos, Position; EA, Effect Allele; OA, Other Allele; EAF, Effect Allele Frequency; Beta, Effect estimate; SE, Standard Error; R2, proportion of variance explained; F, F-statistic

Appendix Table 13. Associations of cis single-nucleotide polymorphisms (SNPs) used as instruments for diazoxide (a vasodilator antihypertensive) from the systolic blood pressure GWAS by Evangelou et al. ^1^

| Gene | SNP | Chr | Pos | EA | OA | EAF | Beta | SE | P-value | R2 | F |
| --- | --- | --- | --- | --- | --- | --- | --- | --- | --- | --- | --- |
| KCNJ11 | rs17470652 | 11 | 16451712 | A | G | 0.601 | 0.216 | 0.031 | 2.716e-12 | 6.694e-05 | 48.864 |
|  | rs4412728 | 11 | 16534954 | A | C | 0.329 | -0.139 | 0.033 | 2.401e-05 | 2.422e-05 | 17.856 |
|  | rs117947332 | 11 | 16685154 | T | C | 0.946 | -0.292 | 0.072 | 5.543e-05 | 2.216e-05 | 16.277 |
|  | rs217754 | 11 | 16844274 | A | T | 0.789 | -0.216 | 0.039 | 2.045e-08 | 4.283e-05 | 31.459 |
|  | rs17770757 | 11 | 16855360 | A | T | 0.029 | -0.454 | 0.093 | 9.627e-07 | 3.248e-05 | 23.975 |
|  | rs7926335 | 11 | 16917869 | T | C | 0.269 | 0.314 | 0.034 | 2.515e-20 | 1.162e-04 | 85.521 |
|  | rs61882040 | 11 | 16925553 | A | C | 0.978 | -0.443 | 0.113 | 8.418e-05 | 2.106e-05 | 15.451 |
|  | rs11820544 | 11 | 16930967 | T | C | 0.922 | 0.233 | 0.057 | 4.081e-05 | 2.284e-05 | 16.811 |
|  | rs4756874 | 11 | 16947185 | T | C | 0.023 | 0.493 | 0.109 | 5.665e-06 | 2.815e-05 | 20.595 |
|  | rs118138471 | 11 | 16956815 | A | C | 0.031 | 0.467 | 0.089 | 1.611e-07 | 3.716e-05 | 27.433 |
|  | rs113343528 | 11 | 17211475 | T | C | 0.934 | -0.298 | 0.067 | 8.101e-06 | 2.718e-05 | 19.908 |
|  | rs12146652 | 11 | 17384542 | A | C | 0.615 | -0.358 | 0.031 | 9.000e-31 | 1.803e-04 | 133.14 |
|  | rs77889556 | 11 | 17498057 | A | G | 0.164 | -0.185 | 0.045 | 3.512e-05 | 2.318e-05 | 17.089 |

Abbreviations: SNP, Single Nucleotide Polymorphism; Chr, Chromosome; Pos, Position; EA, Effect Allele; OA, Other Allele; EAF, Effect Allele Frequency; Beta, Effect estimate; SE, Standard Error; R2, proportion of variance explained; F, F-statistic

Appendix Table 14. Associations of cis single-nucleotide polymorphisms (SNPs) used as instruments for fenoldopam (a vasodilator antihypertensive) from the systolic blood pressure GWAS by Evangelou et al. ^1^

| Gene | SNP | Chr | Pos | EA | OA | EAF | Beta | SE | P-value | R2 | F |
| --- | --- | --- | --- | --- | --- | --- | --- | --- | --- | --- | --- |
| DRD5 | rs11732818 | 4 | 10109123 | A | G | 0.698 | 0.15 | 0.033 | 5.082e-06 | 2.826e-05 | 20.858 |
| DRD1 | rs7378584 | 5 | 175592404 | C | G | 0.529 | -0.14 | 0.030 | 3.888e-06 | 2.905e-05 | 21.269 |

Abbreviations: SNP, Single Nucleotide Polymorphism; Chr, Chromosome; Pos, Position; EA, Effect Allele; OA, Other Allele; EAF, Effect Allele Frequency; Beta, Effect estimate; SE, Standard Error; R2, proportion of variance explained; F, F-statistic

Appendix Table 15. Associations of cis single-nucleotide polymorphisms (SNPs) used as instruments for hydralazine (a vasodilator antihypertensive) from the systolic blood pressure GWAS by Evangelou et al. ^1^

| Gene | SNP | Chr | Pos | EA | OA | EAF | Beta | SE | P-value | R2 | F |
| --- | --- | --- | --- | --- | --- | --- | --- | --- | --- | --- | --- |
| AOC3 | rs73311606 | 17 | 40226750 | A | G | 0.266 | -0.181 | 0.035 | 2.904e-07 | 3.620e-05 | 26.233 |
|  | rs17659391 | 17 | 40266939 | A | G | 0.078 | 0.304 | 0.061 | 6.136e-07 | 3.464e-05 | 24.869 |
|  | rs5016637 | 17 | 40363296 | T | C | 0.822 | 0.17 | 0.043 | 8.744e-05 | 2.083e-05 | 15.396 |
|  | rs12150495 | 17 | 40388430 | A | G | 0.078 | 0.321 | 0.060 | 8.930e-08 | 3.962e-05 | 28.605 |
|  | rs1053004 | 17 | 40466092 | A | G | 0.630 | 0.131 | 0.032 | 3.784e-05 | 2.282e-05 | 16.999 |
|  | rs72823057 | 17 | 40567260 | C | G | 0.073 | 0.259 | 0.059 | 1.223e-05 | 2.569e-05 | 19.135 |
|  | rs11651671 | 17 | 40646803 | A | G | 0.279 | 0.139 | 0.034 | 3.686e-05 | 2.281e-05 | 17.016 |
|  | rs4793069 | 17 | 40801187 | A | G | 0.938 | 0.374 | 0.066 | 1.674e-08 | 4.277e-05 | 31.855 |
|  | rs74754758 | 17 | 40902035 | T | C | 0.982 | 0.489 | 0.123 | 7.111e-05 | 2.131e-05 | 15.793 |
|  | rs12950756 | 17 | 41904372 | T | C | 0.924 | -0.235 | 0.060 | 8.591e-05 | 2.076e-05 | 15.442 |
|  | rs150055076 | 17 | 42009211 | A | G | 0.177 | 0.196 | 0.040 | 7.650e-07 | 3.285e-05 | 24.499 |

Abbreviations: SNP, Single Nucleotide Polymorphism; Chr, Chromosome; Pos, Position; EA, Effect Allele; OA, Other Allele; EAF, Effect Allele Frequency; Beta, Effect estimate; SE, Standard Error; R2, proportion of variance explained; F, F-statistic

Appendix Table 16. Associations of cis single-nucleotide polymorphisms (SNPs) used as instruments for minoxidil (a vasodilator antihypertensive) from the systolic blood pressure GWAS by Evangelou et al. ^1^

| Gene | SNP | Chr | Pos | EA | OA | EAF | Beta | SE | P-value | R2 | F |
| --- | --- | --- | --- | --- | --- | --- | --- | --- | --- | --- | --- |
| KCNJ1 | rs635887 | 11 | 128578047 | T | G | 0.303 | 0.143 | 0.033 | 1.660e-05 | 2.509e-05 | 18.493 |
|  | rs7924416 | 11 | 128764180 | T | C | 0.267 | 0.175 | 0.035 | 7.575e-07 | 3.326e-05 | 24.465 |
|  | rs7126219 | 11 | 129068776 | A | G | 0.093 | -0.212 | 0.052 | 4.969e-05 | 2.230e-05 | 16.463 |
| PTGS1 | rs80257718 | 9 | 124934932 | T | C | 0.022 | -0.517 | 0.107 | 1.440e-06 | 3.116e-05 | 23.217 |
|  | rs2039197 | 9 | 124949046 | A | G | 0.248 | -0.143 | 0.035 | 5.383e-05 | 2.191e-05 | 16.342 |
|  | rs733329 | 9 | 125624164 | T | C | 0.871 | 0.302 | 0.045 | 3.093e-11 | 6.004e-05 | 44.190 |
|  | rs7032325 | 9 | 125917138 | A | G | 0.024 | -0.417 | 0.104 | 6.332e-05 | 2.163e-05 | 16.008 |

Abbreviations: SNP, Single Nucleotide Polymorphism; Chr, Chromosome; Pos, Position; EA, Effect Allele; OA, Other Allele; EAF, Effect Allele Frequency; Beta, Effect estimate; SE, Standard Error; R2, proportion of variance explained; F, F-statistic.

Appendix Table 17. Associations of cis single-nucleotide polymorphisms (SNPs) used as instruments for nitroprusside (a vasodilator antihypertensive) from the systolic blood pressure GWAS by Evangelou et al. ^1^

| Gene | SNP | Chr | Pos | EA | OA | EAF | Beta | SE | P-value | R2 | F |
| --- | --- | --- | --- | --- | --- | --- | --- | --- | --- | --- | --- |
| NPR1 | rs77341498 | 1 | 153728750 | A | G | 0.957 | 0.343 | 0.079 | 1.381e-05 | 2.579e-05 | 18.884 |
|  | rs11264734 | 1 | 153937746 | T | C | 0.708 | 0.141 | 0.033 | 2.187e-05 | 2.436e-05 | 17.986 |
|  | rs6427724 | 1 | 154502499 | A | T | 0.834 | 0.195 | 0.041 | 1.726e-06 | 3.105e-05 | 22.890 |

Abbreviations: SNP, Single Nucleotide Polymorphism; Chr, Chromosome; Pos, Position; EA, Effect Allele; OA, Other Allele; EAF, Effect Allele Frequency; Beta, Effect estimate; SE, Standard Error; R2, proportion of variance explained; F, F-statistic

Appendix Table 18. Associations of cis single-nucleotide polymorphisms (SNPs) used as instruments for adrenergic neuron blockers from the systolic blood pressure GWAS by Keaton et al. ^2^

| Gene | SNP | Chr | Pos | EA | OA | EAF | Beta | SE | P-value | R2 | F |
| --- | --- | --- | --- | --- | --- | --- | --- | --- | --- | --- | --- |
| SLC18A2 | rs72825693 | 10 | 118470758 | T | C | 0.023 | 0.421 | 0.099 | 2.172e-05 | 2.469e-05 | 18.028 |
|  | rs141023517 | 10 | 118493889 | C | T | 0.036 | -0.294 | 0.072 | 4.029e-05 | 1.688e-05 | 16.850 |
|  | rs10787727 | 10 | 118499854 | C | T | 0.316 | -0.162 | 0.026 | 4.439e-10 | 3.872e-05 | 39.014 |
|  | rs61874877 | 10 | 118530973 | A | G | 0.299 | 0.188 | 0.027 | 1.461e-12 | 4.978e-05 | 50.165 |
|  | rs11198102 | 10 | 119552607 | A | G | 0.052 | 0.270 | 0.069 | 9.508e-05 | 2.088e-05 | 15.245 |
| SLC6A2 | rs147750591 | 16 | 55817124 | A | C | 0.015 | 0.530 | 0.135 | 8.747e-05 | 2.109e-05 | 15.396 |
|  | rs13337678 | 16 | 56379937 | T | C | 0.568 | -0.105 | 0.025 | 2.091e-05 | 1.782e-05 | 18.114 |

Abbreviations: SNP, Single Nucleotide Polymorphisms; Chr, Chromosome; Pos, Position; EA, Effect Allele; OA, Other Allele; EAF, Effect Allele Frequency; Beta, Effect estimate; SE, Standard Error; R2, proportion of variance explained; F, F-statistic

Appendix Table 19. Associations of cis single-nucleotide polymorphisms (SNPs) used as instruments for alpha-adrenoceptor antagonists from the systolic blood pressure GWAS by Keaton et al. ^2^

| Gene | SNP | Chr | Pos | EA | OA | EAF | Beta | SE | P-value | R2 | F |
| --- | --- | --- | --- | --- | --- | --- | --- | --- | --- | --- | --- |
| ADRA1A | rs2278143 | 8 | 25634577 | G | A | 0.134 | -0.217 | 0.036 | 1.807e-09 | 3.592e-05 | 36.233 |
|  | rs7843840 | 8 | 25852017 | G | A | 0.272 | -0.164 | 0.027 | 1.838e-09 | 3.569e-05 | 36.000 |
|  | rs62499124 | 8 | 25862499 | A | G | 0.291 | 0.123 | 0.027 | 4.193e-06 | 2.106e-05 | 21.222 |
|  | rs75523934 | 8 | 25885441 | C | T | 0.024 | -0.348 | 0.080 | 1.237e-05 | 1.895e-05 | 19.124 |
|  | rs4871964 | 8 | 25908797 | G | A | 0.251 | -0.312 | 0.028 | 3.634e-29 | 1.252e-04 | 125.38 |
|  | rs74682804 | 8 | 25914683 | C | G | 0.021 | -0.384 | 0.087 | 1.024e-05 | 1.928e-05 | 19.471 |
|  | rs62501779 | 8 | 25932777 | G | A | 0.227 | 0.172 | 0.029 | 4.881e-09 | 3.389e-05 | 34.226 |
|  | rs13439370 | 8 | 25935564 | G | C | 0.283 | 0.145 | 0.029 | 4.386e-07 | 2.523e-05 | 25.455 |
|  | rs7003131 | 8 | 26013917 | A | G | 0.264 | -0.172 | 0.027 | 3.797e-10 | 3.884e-05 | 39.222 |
|  | rs9650417 | 8 | 26029798 | A | G | 0.226 | -0.16 | 0.030 | 6.002e-08 | 2.901e-05 | 29.291 |
|  | rs34788903 | 8 | 26063734 | C | T | 0.106 | 0.324 | 0.041 | 1.496e-15 | 6.307e-05 | 63.528 |
|  | rs73215676 | 8 | 26115803 | G | A | 0.035 | 0.300 | 0.068 | 1.159e-05 | 1.905e-05 | 19.232 |
|  | rs1064807 | 8 | 26236978 | T | C | 0.696 | -0.128 | 0.027 | 1.628e-06 | 2.298e-05 | 23.018 |
|  | rs141808794 | 8 | 26266252 | A | G | 0.021 | -0.421 | 0.088 | 1.797e-06 | 2.259e-05 | 22.792 |
|  | rs77025005 | 8 | 26352264 | A | T | 0.039 | -0.258 | 0.064 | 5.886e-05 | 1.602e-05 | 16.162 |
|  | rs1372882 | 8 | 26376559 | A | G | 0.755 | 0.136 | 0.028 | 1.357e-06 | 2.309e-05 | 23.287 |
|  | rs7829347 | 8 | 26480617 | C | T | 0.098 | 0.240 | 0.041 | 3.339e-09 | 3.466e-05 | 35.002 |
|  | rs7835853 | 8 | 26662246 | T | C | 0.208 | 0.174 | 0.030 | 1.117e-08 | 3.234e-05 | 32.658 |
|  | rs61762862 | 8 | 26698037 | G | T | 0.056 | 0.235 | 0.054 | 1.534e-05 | 1.854e-05 | 18.698 |
|  | rs1842646 | 8 | 26743933 | C | A | 0.393 | -0.099 | 0.025 | 6.220e-05 | 1.591e-05 | 16.065 |
|  | rs141804347 | 8 | 26860769 | A | G | 0.086 | -0.195 | 0.045 | 1.370e-05 | 1.872e-05 | 18.900 |
|  | rs10088622 | 8 | 26962142 | T | C | 0.517 | -0.121 | 0.024 | 7.054e-07 | 2.455e-05 | 24.551 |
|  | rs2004539 | 8 | 27587105 | T | C | 0.442 | -0.121 | 0.024 | 8.443e-07 | 2.408e-05 | 24.311 |
| ADRA1D | rs616749 | 20 | 3642646 | T | C | 0.100 | 0.187 | 0.040 | 3.627e-06 | 2.113e-05 | 21.471 |
|  | rs4815660 | 20 | 4095716 | A | G | 0.662 | -0.130 | 0.025 | 2.697e-07 | 2.608e-05 | 26.531 |
|  | rs1764975 | 20 | 4101290 | A | T | 0.799 | 0.235 | 0.030 | 8.705e-15 | 5.933e-05 | 60.306 |
|  | rs6139317 | 20 | 4104510 | A | T | 0.365 | 0.134 | 0.026 | 2.118e-07 | 2.638e-05 | 26.815 |
|  | rs297738 | 20 | 4413512 | A | G | 0.275 | -0.128 | 0.027 | 2.109e-06 | 2.209e-05 | 22.475 |
|  | rs2203908 | 20 | 4969110 | G | A | 0.437 | -0.101 | 0.025 | 5.117e-05 | 1.613e-05 | 16.355 |
| ADRA1B | rs2072495 | 5 | 158296996 | T | C | 0.393 | 0.271 | 0.025 | 2.986e-28 | 1.203e-04 | 121.000 |
|  | rs17626412 | 5 | 158338972 | A | G | 0.048 | 0.262 | 0.061 | 1.549e-05 | 1.852e-05 | 18.645 |
|  | rs80066419 | 5 | 158347020 | A | C | 0.073 | -0.337 | 0.047 | 1.178e-12 | 5.015e-05 | 50.488 |
|  | rs62385381 | 5 | 158350706 | T | C | 0.033 | -0.372 | 0.072 | 2.109e-07 | 2.674e-05 | 26.918 |
|  | rs11742026 | 5 | 158353863 | G | A | 0.038 | -0.318 | 0.066 | 1.22e-06 | 2.338e-05 | 23.541 |
|  | rs113255182 | 5 | 158355949 | G | A | 0.054 | 0.325 | 0.058 | 1.985e-08 | 3.133e-05 | 31.476 |
|  | rs147405800 | 5 | 158412406 | T | G | 0.028 | -0.462 | 0.075 | 8.890e-10 | 3.737e-05 | 37.595 |
|  | rs34296591 | 5 | 158444231 | G | C | 0.350 | -0.196 | 0.026 | 3.008e-14 | 5.760e-05 | 57.926 |
|  | rs76688984 | 5 | 158474697 | T | C | 0.102 | 0.188 | 0.040 | 2.891e-06 | 2.173e-05 | 21.878 |
|  | rs7733049 | 5 | 158489476 | C | G | 0.314 | -0.116 | 0.026 | 1.241e-05 | 1.899e-05 | 19.095 |
|  | rs12659540 | 5 | 158522577 | A | C | 0.499 | 0.120 | 0.025 | 1.316e-06 | 2.332e-05 | 23.452 |
|  | rs6895454 | 5 | 158523167 | T | C | 0.078 | -0.280 | 0.046 | 1.090e-09 | 3.691e-05 | 37.159 |
|  | rs67867295 | 5 | 158728264 | C | T | 0.105 | -0.177 | 0.042 | 2.290e-05 | 1.781e-05 | 17.910 |
|  | rs4921492 | 5 | 158832277 | A | C | 0.347 | -0.110 | 0.026 | 2.240e-05 | 1.784e-05 | 17.940 |
|  | rs72806298 | 5 | 159135376 | T | A | 0.146 | 0.156 | 0.036 | 1.249e-05 | 1.893e-05 | 19.061 |
|  | rs72808188 | 5 | 159336731 | C | T | 0.060 | -0.218 | 0.052 | 2.876e-05 | 1.744e-05 | 17.495 |
|  | rs2546973 | 5 | 159573778 | A | C | 0.436 | 0.098 | 0.025 | 9.567e-05 | 1.516e-05 | 15.244 |
|  | rs116286399 | 5 | 159576612 | A | G | 0.064 | -0.224 | 0.053 | 2.359e-05 | 1.781e-05 | 17.882 |
| ADRA2A | rs61882777 | 10 | 111962102 | C | T | 0.096 | 0.219 | 0.042 | 2.052e-07 | 2.673e-05 | 26.961 |
|  | rs17127310 | 10 | 112137261 | A | G | 0.267 | -0.126 | 0.028 | 5.734e-06 | 2.033e-05 | 20.525 |
|  | rs11195157 | 10 | 112259493 | C | G | 0.328 | 0.112 | 0.026 | 1.394e-05 | 1.871e-05 | 18.890 |
|  | rs73356958 | 10 | 112485081 | T | C | 0.178 | 0.152 | 0.032 | 1.809e-06 | 2.260e-05 | 22.824 |
|  | rs2419580 | 10 | 112500340 | G | T | 0.814 | 0.152 | 0.031 | 1.083e-06 | 2.351e-05 | 23.738 |
|  | rs7894045 | 10 | 113511441 | G | A | 0.630 | -0.104 | 0.025 | 4.148e-05 | 1.683e-05 | 16.829 |
|  | rs6585121 | 10 | 113612431 | G | A | 0.438 | 0.106 | 0.024 | 1.268e-05 | 1.889e-05 | 19.077 |

Abbreviations: SNP, Single Nucleotide Polymorphisms; Chr, Chromosome; Pos, Position; EA, Effect Allele; OA, Other Allele; EAF, Effect Allele Frequency; Beta, Effect estimate; SE, Standard Error; R2, proportion of variance explained; F, F-statistic

Appendix Table 20. Associations of cis single-nucleotide polymorphisms (SNPs) used as instruments for angiotensin-converting enzyme inhibitors from the systolic blood pressure GWAS by Keaton et al. ^2^

| Gene | SNP | Chr | Pos | EA | OA | EAF | Beta | SE | P-value | R2 | F |
| --- | --- | --- | --- | --- | --- | --- | --- | --- | --- | --- | --- |
| ACE | rs8078112 | 17 | 60720362 | T | C | 0.574 | -0.159 | 0.025 | 9.085e-11 | 4.121e-05 | 41.933 |
|  | rs3826537 | 17 | 60769803 | G | A | 0.419 | 0.199 | 0.024 | 4.598e-16 | 6.458e-05 | 65.709 |
|  | rs62076622 | 17 | 61090958 | G | A | 0.199 | -0.148 | 0.030 | 1.249e-06 | 2.314e-05 | 23.546 |
|  | rs8077276 | 17 | 61547562 | A | G | 0.620 | -0.231 | 0.025 | 2.819e-20 | 8.363e-05 | 84.919 |
|  | rs4312 | 17 | 61560986 | T | A | 0.061 | -0.239 | 0.052 | 3.564e-06 | 2.156e-05 | 21.489 |
|  | rs8066276 | 17 | 61589265 | C | T | 0.588 | 0.097 | 0.025 | 8.583e-05 | 1.529e-05 | 15.425 |
|  | rs79480822 | 17 | 61654225 | T | C | 0.051 | 0.254 | 0.062 | 4.110e-05 | 1.696e-05 | 16.810 |
|  | rs11655833 | 17 | 62083212 | A | T | 0.096 | -0.218 | 0.046 | 1.855e-06 | 2.265e-05 | 22.734 |
|  | rs940744 | 17 | 62293122 | G | A | 0.783 | 0.138 | 0.030 | 4.133e-06 | 2.088e-05 | 21.221 |
|  | rs112300378 | 17 | 62317177 | C | A | 0.077 | 0.357 | 0.071 | 5.239e-07 | 3.453e-05 | 25.211 |
|  | rs6504213 | 17 | 62381714 | C | T | 0.584 | 0.242 | 0.025 | 6.253e-22 | 9.122e-05 | 92.727 |
|  | rs11655644 | 17 | 62393621 | T | C | 0.064 | -0.302 | 0.059 | 3.008e-07 | 2.607e-05 | 26.255 |
|  | rs17650301 | 17 | 62479273 | C | A | 0.302 | -0.188 | 0.027 | 4.633e-12 | 4.780e-05 | 47.823 |
|  | rs41548920 | 17 | 62493390 | G | A | 0.020 | -0.459 | 0.094 | 1.003e-06 | 2.388e-05 | 23.905 |

Abbreviations: SNP, Single Nucleotide Polymorphisms; Chr, Chromosome; Pos, Position; EA, Effect Allele; OA, Other Allele; EAF, Effect Allele Frequency; Beta, Effect estimate; SE, Standard Error; R2, proportion of variance explained; F, F-statistic

Appendix Table 21. Associations of cis single-nucleotide polymorphisms (SNPs) used as instruments for angiotensin-II receptor blockers from the systolic blood pressure GWAS by Keaton et al. ^2^

| Gene | SNP | Chr | Pos | EA | OA | EAF | Beta | SE | P-value | R2 | F |
| --- | --- | --- | --- | --- | --- | --- | --- | --- | --- | --- | --- |
| AGTR1 | rs1870000 | 3 | 147787591 | T | C | 0.657 | 0.108 | 0.026 | 2.403e-05 | 1.780e-05 | 17.823 |
|  | rs11707568 | 3 | 148511662 | C | A | 0.255 | 0.156 | 0.029 | 1.082e-07 | 2.798e-05 | 28.227 |
|  | rs4681161 | 3 | 148609045 | C | T | 0.563 | 0.134 | 0.024 | 3.481e-08 | 3.020e-05 | 30.499 |
|  | rs2320021 | 3 | 148680871 | C | A | 0.597 | 0.105 | 0.025 | 2.256e-05 | 1.781e-05 | 17.960 |

Abbreviations: SNP, Single Nucleotide Polymorphisms; Chr, Chromosome; Pos, Position; EA, Effect Allele; OA, Other Allele; EAF, Effect Allele Frequency; Beta, Effect estimate; SE, Standard Error; R2, proportion of variance explained; F, F-statistic

Appendix Table 22. Associations of cis single-nucleotide polymorphisms (SNPs) used as instruments for beta-adrenoceptor antagonists from the systolic blood pressure GWAS by Keaton et al. ^2^

| Gene | SNP | Chr | Pos | EA | OA | EAF | Beta | SE | P-value | R2 | F |
| --- | --- | --- | --- | --- | --- | --- | --- | --- | --- | --- | --- |
| ADRB1 | rs11196211 | 10 | 114817009 | C | A | 0.285 | 0.231 | 0.027 | 1.709e-17 | 7.176e-05 | 72.312 |
|  | rs3829909 | 10 | 114831434 | T | A | 0.275 | 0.210 | 0.028 | 9.006e-14 | 5.515e-05 | 55.638 |
|  | rs749596 | 10 | 114847905 | A | T | 0.204 | -0.166 | 0.031 | 7.749e-08 | 2.869e-05 | 28.908 |
|  | rs10885421 | 10 | 114916586 | T | G | 0.598 | 0.141 | 0.026 | 3.809e-08 | 2.990e-05 | 30.164 |
|  | rs141111362 | 10 | 115389129 | T | A | 0.021 | 0.354 | 0.088 | 5.174e-05 | 1.623e-05 | 16.386 |
|  | rs75228369 | 10 | 115684914 | T | C | 0.047 | 0.261 | 0.061 | 2.163e-05 | 1.789e-05 | 18.069 |
|  | rs151598 | 10 | 115697178 | G | A | 0.573 | 0.153 | 0.024 | 2.852e-10 | 3.936e-05 | 39.747 |
|  | rs7894582 | 10 | 115704458 | A | C | 0.046 | -0.258 | 0.059 | 1.162e-05 | 1.907e-05 | 19.252 |
|  | rs11196549 | 10 | 115707298 | A | G | 0.044 | 0.535 | 0.061 | 1.499e-18 | 7.654e-05 | 77.290 |
|  | rs460718 | 10 | 115721364 | G | A | 0.672 | 0.212 | 0.026 | 1.309e-16 | 6.770e-05 | 68.368 |
|  | rs1411407 | 10 | 115747786 | C | T | 0.529 | 0.120 | 0.025 | 2.036e-06 | 2.227e-05 | 22.487 |
|  | rs678669 | 10 | 115764032 | T | C | 0.605 | -0.115 | 0.025 | 4.616e-06 | 2.101e-05 | 21.028 |
|  | rs116866627 | 10 | 115774356 | C | G | 0.021 | -0.667 | 0.114 | 4.166e-09 | 4.728e-05 | 34.525 |
|  | rs11196597 | 10 | 115788094 | A | G | 0.135 | 0.264 | 0.038 | 2.027e-12 | 4.898e-05 | 49.411 |
|  | rs1801253 | 10 | 115805056 | C | G | 0.732 | 0.399 | 0.028 | 9.758e-47 | 2.040e-04 | 205.99 |
|  | rs148422600 | 10 | 115829233 | A | C | 0.027 | -0.416 | 0.080 | 1.932e-07 | 2.694e-05 | 27.105 |
|  | rs68122733 | 10 | 115831533 | G | A | 0.171 | -0.248 | 0.032 | 2.405e-14 | 5.762e-05 | 58.182 |
|  | rs143854972 | 10 | 115843445 | A | G | 0.057 | 0.354 | 0.054 | 4.622e-11 | 4.294e-05 | 43.359 |
|  | rs11196625 | 10 | 115843990 | A | G | 0.188 | 0.211 | 0.032 | 3.838e-11 | 4.327e-05 | 43.601 |
|  | rs11196653 | 10 | 115952212 | T | C | 0.015 | -0.448 | 0.111 | 5.658e-05 | 1.609e-05 | 16.224 |
|  | rs4751648 | 10 | 116132246 | T | C | 0.559 | 0.124 | 0.024 | 3.478e-07 | 2.576e-05 | 25.955 |
|  | rs1968122 | 10 | 116463982 | C | T | 0.548 | 0.130 | 0.029 | 6.379e-06 | 2.584e-05 | 20.391 |
| ADRB2 | rs7730971 | 5 | 147790860 | G | C | 0.580 | -0.132 | 0.026 | 2.780e-07 | 2.646e-05 | 26.380 |
|  | rs9716164 | 5 | 147884668 | T | C | 0.417 | 0.182 | 0.024 | 1.007e-13 | 5.514e-05 | 55.515 |
|  | rs56373125 | 5 | 147889702 | G | A | 0.062 | -0.308 | 0.053 | 7.317e-09 | 3.328e-05 | 33.501 |
|  | rs1432919 | 5 | 147911872 | A | G | 0.048 | 0.253 | 0.058 | 1.264e-05 | 1.894e-05 | 19.073 |
|  | rs2400643 | 5 | 148114897 | G | A | 0.229 | -0.119 | 0.029 | 3.708e-05 | 1.701e-05 | 16.983 |
|  | rs917875 | 5 | 148316076 | C | A | 0.044 | -0.391 | 0.061 | 1.589e-10 | 4.063e-05 | 40.868 |
|  | rs10491337 | 5 | 148331046 | T | G | 0.048 | -0.372 | 0.06 | 7.003e-10 | 3.776e-05 | 38.014 |
|  | rs4705300 | 5 | 148336213 | A | G | 0.328 | 0.164 | 0.026 | 3.343e-10 | 3.920e-05 | 39.386 |
|  | rs10477399 | 5 | 148347543 | A | G | 0.191 | -0.331 | 0.031 | 4.587e-26 | 1.104e-04 | 111.19 |
|  | rs113096032 | 5 | 148452317 | C | T | 0.025 | -0.398 | 0.082 | 1.031e-06 | 2.387e-05 | 23.849 |
|  | rs79526330 | 5 | 148493315 | C | T | 0.015 | -0.502 | 0.104 | 1.460e-06 | 2.316e-05 | 23.184 |
|  | rs4705316 | 5 | 148498799 | A | G | 0.759 | -0.151 | 0.028 | 7.758e-08 | 2.876e-05 | 28.953 |
|  | rs114464628 | 5 | 148533724 | A | G | 0.038 | 0.265 | 0.067 | 7.037e-05 | 1.583e-05 | 15.820 |
|  | rs4705067 | 5 | 148542406 | G | C | 0.788 | 0.124 | 0.030 | 3.639e-05 | 1.699e-05 | 17.084 |
|  | rs2895838 | 5 | 148628555 | T | C | 0.482 | 0.108 | 0.024 | 1.088e-05 | 1.919e-05 | 19.324 |
|  | rs12518020 | 5 | 148839790 | A | G | 0.320 | 0.118 | 0.027 | 8.685e-06 | 1.967e-05 | 19.779 |
|  | rs34953047 | 5 | 148957101 | C | T | 0.251 | 0.109 | 0.028 | 8.626e-05 | 1.533e-05 | 15.430 |
|  | rs1124995 | 5 | 149117004 | C | T | 0.777 | 0.114 | 0.029 | 9.556e-05 | 1.521e-05 | 15.189 |

Abbreviations: SNP, Single Nucleotide Polymorphisms; Chr, Chromosome; Pos, Position; EA, Effect Allele; OA, Other Allele; EAF, Effect Allele Frequency; Beta, Effect estimate; SE, Standard Error; R2, proportion of variance explained; F, F-statistic

Appendix Table 23. Associations of cis single-nucleotide polymorphisms (SNPs) used as instruments for calcium channel blockers from the systolic blood pressure GWAS by Keaton et al. ^2^

| Gene | SNP | Chr | Pos | EA | OA | EAF | Beta | SE | P-value | R2 | F |
| --- | --- | --- | --- | --- | --- | --- | --- | --- | --- | --- | --- |
| CACNA1C | rs143459027 | 12 | 2212867 | T | C | 0.030 | 0.365 | 0.078 | 2.847e-06 | 2.158e-05 | 21.942 |
|  | rs3819536 | 12 | 2436998 | A | G | 0.705 | 0.176 | 0.026 | 2.249e-11 | 4.446e-05 | 44.869 |
|  | rs714277 | 12 | 2514270 | T | C | 0.281 | 0.180 | 0.027 | 1.442e-11 | 4.491e-05 | 45.689 |
|  | rs4765961 | 12 | 2668472 | C | T | 0.170 | -0.176 | 0.033 | 7.131e-08 | 2.846e-05 | 28.956 |
|  | rs56196860 | 12 | 2908330 | A | C | 0.031 | -0.393 | 0.083 | 2.266e-06 | 3.058e-05 | 22.332 |
|  | rs11612237 | 12 | 3031161 | C | A | 0.021 | 0.380 | 0.090 | 2.404e-05 | 1.762e-05 | 17.860 |
|  | rs242037 | 12 | 3780337 | A | G | 0.252 | -0.113 | 0.028 | 5.307e-05 | 1.601e-05 | 16.286 |
| CACNA1S | rs3850625 | 1 | 201016296 | A | G | 0.118 | 0.149 | 0.037 | 6.311e-05 | 1.587e-05 | 16.021 |
|  | rs10920179 | 1 | 201326114 | T | C | 0.535 | -0.099 | 0.025 | 9.665e-05 | 1.504e-05 | 15.188 |
|  | rs2644127 | 1 | 201886884 | C | T | 0.028 | 0.324 | 0.078 | 3.605e-05 | 1.692e-05 | 17.088 |
| CACNA1D | rs10433615 | 3 | 52638482 | T | C | 0.396 | 0.134 | 0.025 | 5.430e-08 | 2.941e-05 | 29.671 |
|  | rs77347777 | 3 | 52848207 | T | C | 0.118 | -0.248 | 0.040 | 3.350e-10 | 3.920e-05 | 39.547 |
|  | rs62253653 | 3 | 53013267 | G | A | 0.291 | -0.108 | 0.027 | 7.200e-05 | 1.566e-05 | 15.795 |
|  | rs79270291 | 3 | 53173018 | G | A | 0.013 | 0.572 | 0.120 | 1.865e-06 | 2.257e-05 | 22.745 |
|  | rs62254274 | 3 | 53180805 | G | A | 0.133 | -0.148 | 0.036 | 3.520e-05 | 1.697e-05 | 17.137 |
|  | rs41332244 | 3 | 53282033 | T | C | 0.063 | -0.242 | 0.050 | 1.269e-06 | 2.327e-05 | 23.426 |
|  | rs114718455 | 3 | 53464055 | G | A | 0.032 | 0.425 | 0.077 | 3.234e-08 | 3.050e-05 | 30.573 |
|  | rs3821843 | 3 | 53558012 | A | G | 0.683 | 0.307 | 0.027 | 6.286e-30 | 1.284e-04 | 129.37 |
|  | rs114987861 | 3 | 53605712 | A | G | 0.028 | 0.431 | 0.077 | 1.877e-08 | 3.136e-05 | 31.635 |
|  | rs113210396 | 3 | 53612327 | T | G | 0.044 | -0.335 | 0.064 | 1.639e-07 | 2.719e-05 | 27.431 |
|  | rs3774458 | 3 | 53617868 | A | G | 0.288 | 0.126 | 0.027 | 3.726e-06 | 2.127e-05 | 21.459 |
|  | rs116535016 | 3 | 53645516 | A | G | 0.021 | 0.382 | 0.090 | 1.911e-05 | 1.810e-05 | 18.265 |
|  | rs34802409 | 3 | 53673715 | A | G | 0.200 | -0.173 | 0.030 | 1.165e-08 | 3.222e-05 | 32.535 |
|  | rs184974745 | 3 | 53711837 | A | C | 0.044 | 0.239 | 0.061 | 9.264e-05 | 1.517e-05 | 15.302 |
|  | rs9841978 | 3 | 53730735 | A | G | 0.326 | 0.207 | 0.026 | 1.946e-15 | 6.241e-05 | 63.023 |
|  | rs3821864 | 3 | 53797095 | T | C | 0.014 | -0.444 | 0.110 | 5.759e-05 | 1.608e-05 | 16.174 |
|  | rs2241808 | 3 | 53856653 | T | C | 0.537 | 0.105 | 0.024 | 1.557e-05 | 1.851e-05 | 18.671 |
|  | rs62250937 | 3 | 53870318 | C | T | 0.090 | -0.261 | 0.045 | 8.616e-09 | 3.294e-05 | 33.196 |
|  | rs60978131 | 3 | 53982904 | T | C | 0.015 | 0.520 | 0.117 | 8.364e-06 | 1.980e-05 | 19.866 |
|  | rs4687767 | 3 | 54027442 | C | T | 0.095 | -0.203 | 0.042 | 9.627e-07 | 2.372e-05 | 23.951 |
| CACNB2 | rs41289319 | 10 | 17201063 | C | T | 0.029 | -0.334 | 0.082 | 4.539e-05 | 1.680e-05 | 16.651 |
|  | rs10904910 | 10 | 17266389 | A | C | 0.310 | 0.155 | 0.026 | 2.559e-09 | 3.524e-05 | 35.586 |
|  | rs560358 | 10 | 18228818 | C | A | 0.190 | -0.133 | 0.032 | 2.584e-05 | 1.761e-05 | 17.747 |
|  | rs2437265 | 10 | 18273428 | G | A | 0.623 | -0.118 | 0.025 | 2.520e-06 | 2.191e-05 | 22.101 |
|  | rs7894090 | 10 | 18291288 | A | G | 0.853 | -0.230 | 0.034 | 2.143e-11 | 4.449e-05 | 44.925 |
|  | rs11492530 | 10 | 18308610 | T | A | 0.020 | 0.404 | 0.094 | 1.818e-05 | 1.834e-05 | 18.363 |
|  | rs2488136 | 10 | 18334521 | G | A | 0.710 | -0.199 | 0.026 | 6.027e-14 | 5.590e-05 | 56.448 |
|  | rs10828165 | 10 | 18349535 | G | A | 0.172 | -0.130 | 0.033 | 9.668e-05 | 1.508e-05 | 15.194 |
|  | rs2497798 | 10 | 18349822 | T | G | 0.124 | 0.200 | 0.039 | 2.234e-07 | 2.659e-05 | 26.788 |
|  | rs77465184 | 10 | 18352643 | C | T | 0.046 | 0.335 | 0.061 | 3.996e-08 | 3.013e-05 | 30.178 |
|  | rs2357786 | 10 | 18355749 | C | T | 0.551 | -0.187 | 0.024 | 1.344e-14 | 5.883e-05 | 59.410 |
|  | rs76304369 | 10 | 18359213 | T | C | 0.012 | 0.668 | 0.112 | 2.553e-09 | 3.528e-05 | 35.509 |
|  | rs75699707 | 10 | 18359294 | A | G | 0.025 | -0.460 | 0.085 | 6.233e-08 | 2.922e-05 | 29.269 |
|  | rs78351253 | 10 | 18361384 | G | A | 0.041 | -0.331 | 0.063 | 1.508e-07 | 2.729e-05 | 27.554 |
|  | rs78607408 | 10 | 18386117 | C | T | 0.044 | -0.254 | 0.062 | 3.652e-05 | 1.689e-05 | 17.056 |
|  | rs189315389 | 10 | 18387884 | T | A | 0.038 | 0.395 | 0.065 | 1.125e-09 | 3.670e-05 | 37.062 |
|  | rs117299889 | 10 | 18404550 | C | T | 0.050 | 0.263 | 0.059 | 9.041e-06 | 1.955e-05 | 19.715 |
|  | rs11012734 | 10 | 18405972 | G | A | 0.403 | 0.118 | 0.025 | 2.588e-06 | 2.185e-05 | 22.038 |
|  | rs7900124 | 10 | 18427907 | C | T | 0.304 | 0.270 | 0.026 | 1.524e-24 | 1.039e-04 | 104.83 |
|  | rs142857446 | 10 | 18429273 | T | C | 0.013 | -0.560 | 0.120 | 3.229e-06 | 2.167e-05 | 21.674 |
|  | rs150041732 | 10 | 18434889 | C | T | 0.027 | -0.399 | 0.086 | 3.037e-06 | 2.188e-05 | 21.810 |
|  | rs78122842 | 10 | 18443866 | A | G | 0.021 | 0.393 | 0.088 | 7.372e-06 | 1.994e-05 | 20.112 |
|  | rs1779240 | 10 | 18476313 | A | G | 0.766 | -0.386 | 0.028 | 7.241e-42 | 1.817e-04 | 183.53 |
|  | rs61278674 | 10 | 18481737 | G | A | 0.093 | 0.297 | 0.044 | 8.121e-12 | 4.627e-05 | 46.679 |
|  | rs11012983 | 10 | 18490601 | T | A | 0.109 | 0.231 | 0.039 | 4.345e-09 | 3.429e-05 | 34.493 |
|  | rs1779209 | 10 | 18514561 | C | T | 0.710 | -0.245 | 0.027 | 4.417e-20 | 8.431e-05 | 84.406 |
|  | rs1757234 | 10 | 18526451 | A | G | 0.123 | -0.194 | 0.037 | 1.727e-07 | 2.711e-05 | 27.378 |
|  | rs10828399 | 10 | 18553968 | A | G | 0.526 | -0.171 | 0.024 | 1.402e-12 | 4.974e-05 | 50.227 |
|  | rs9633646 | 10 | 18564922 | A | G | 0.594 | -0.119 | 0.025 | 1.365e-06 | 2.313e-05 | 23.361 |
|  | rs79666207 | 10 | 18583840 | C | T | 0.018 | -0.461 | 0.100 | 4.271e-06 | 2.095e-05 | 21.140 |
|  | rs113955098 | 10 | 18591226 | A | G | 0.024 | 0.386 | 0.093 | 3.329e-05 | 1.725e-05 | 17.219 |
|  | rs10828452 | 10 | 18592450 | T | A | 0.212 | -0.232 | 0.031 | 6.879e-14 | 5.562e-05 | 56.105 |
|  | rs74494383 | 10 | 18626340 | A | C | 0.021 | 0.410 | 0.092 | 9.122e-06 | 1.967e-05 | 19.679 |
|  | rs10828542 | 10 | 18627285 | G | A | 0.387 | -0.148 | 0.025 | 3.211e-09 | 3.480e-05 | 35.141 |
|  | rs12780039 | 10 | 18678987 | C | G | 0.118 | 0.249 | 0.038 | 6.753e-11 | 4.216e-05 | 42.575 |
|  | rs2148184 | 10 | 18680963 | C | T | 0.133 | 0.200 | 0.036 | 3.103e-08 | 3.035e-05 | 30.646 |
|  | rs61839258 | 10 | 18689638 | T | G | 0.169 | 0.150 | 0.032 | 3.667e-06 | 2.123e-05 | 21.415 |
|  | rs112133583 | 10 | 18695681 | T | C | 0.029 | -0.552 | 0.082 | 1.484e-11 | 4.512e-05 | 45.521 |
|  | rs79615672 | 10 | 18700784 | T | C | 0.024 | -0.443 | 0.087 | 3.710e-07 | 2.580e-05 | 25.821 |
|  | rs11014170 | 10 | 18710991 | A | G | 0.020 | -0.571 | 0.095 | 1.784e-09 | 3.607e-05 | 36.203 |
|  | rs117605949 | 10 | 18715104 | G | T | 0.019 | -0.404 | 0.096 | 2.707e-05 | 1.743e-05 | 17.598 |
|  | rs181333405 | 10 | 18716779 | G | A | 0.014 | -0.578 | 0.140 | 3.740e-05 | 2.329e-05 | 17.003 |
|  | rs7923191 | 10 | 18727901 | G | A | 0.209 | 0.323 | 0.030 | 1.464e-26 | 1.124e-04 | 113.43 |
|  | rs12258967 | 10 | 18727959 | G | C | 0.297 | -0.546 | 0.027 | 1.430e-90 | 4.046e-04 | 408.34 |
|  | rs72786098 | 10 | 18729855 | A | G | 0.031 | -0.462 | 0.074 | 3.330e-10 | 3.910e-05 | 39.442 |
|  | rs71497251 | 10 | 18781265 | A | C | 0.183 | 0.167 | 0.032 | 2.103e-07 | 2.664e-05 | 26.866 |
|  | rs7080574 | 10 | 18788342 | C | G | 0.416 | -0.143 | 0.027 | 1.738e-07 | 2.700e-05 | 27.238 |
|  | rs144963887 | 10 | 18788357 | T | C | 0.018 | -0.498 | 0.111 | 7.567e-06 | 2.003e-05 | 20.052 |
|  | rs4748474 | 10 | 18790727 | A | G | 0.524 | 0.156 | 0.024 | 1.504e-10 | 4.108e-05 | 41.138 |
|  | rs7082751 | 10 | 18991623 | A | T | 0.778 | 0.128 | 0.029 | 1.151e-05 | 1.922e-05 | 19.246 |
|  | rs180849797 | 10 | 19340761 | A | G | 0.015 | 0.546 | 0.116 | 2.627e-06 | 2.198e-05 | 22.081 |
|  | rs12219073 | 10 | 19830504 | G | C | 0.470 | -0.099 | 0.025 | 5.895e-05 | 1.594e-05 | 16.098 |
| CACNA1H | rs9923059 | 16 | 381590 | A | C | 0.358 | 0.118 | 0.030 | 7.324e-05 | 2.158e-05 | 15.758 |
|  | rs6600229 | 16 | 707295 | G | T | 0.455 | 0.137 | 0.026 | 1.799e-07 | 2.737e-05 | 27.175 |
|  | rs12448432 | 16 | 778820 | A | G | 0.216 | 0.115 | 0.030 | 9.997e-05 | 1.508e-05 | 15.173 |
|  | rs117177120 | 16 | 1238875 | G | T | 0.058 | 0.287 | 0.064 | 8.676e-06 | 2.708e-05 | 19.771 |
|  | rs151059668 | 16 | 1333219 | A | G | 0.016 | 0.546 | 0.119 | 4.696e-06 | 2.869e-05 | 20.951 |
|  | rs12920764 | 16 | 1362187 | T | C | 0.607 | -0.166 | 0.025 | 3.504e-11 | 4.332e-05 | 43.983 |
|  | rs4984836 | 16 | 1481797 | G | A | 0.883 | -0.229 | 0.038 | 1.739e-09 | 3.577e-05 | 36.316 |
|  | rs2092387 | 16 | 1534320 | A | G | 0.066 | 0.251 | 0.050 | 5.961e-07 | 2.451e-05 | 24.881 |
|  | rs2235641 | 16 | 1608177 | G | A | 0.260 | 0.110 | 0.027 | 5.278e-05 | 1.623e-05 | 16.383 |
|  | rs2575358 | 16 | 1978487 | C | T | 0.378 | 0.111 | 0.026 | 1.910e-05 | 1.801e-05 | 18.217 |
|  | rs111845910 | 16 | 1991014 | A | G | 0.070 | 0.242 | 0.056 | 1.409e-05 | 2.584e-05 | 18.871 |
|  | rs1860236 | 16 | 2023372 | C | A | 0.154 | 0.202 | 0.035 | 9.532e-09 | 3.317e-05 | 32.956 |
|  | rs113097793 | 16 | 2037831 | A | G | 0.188 | -0.211 | 0.031 | 9.598e-12 | 4.588e-05 | 46.540 |
|  | rs55662859 | 16 | 2052806 | A | G | 0.100 | 0.277 | 0.042 | 6.459e-11 | 4.260e-05 | 42.789 |
|  | rs28590346 | 16 | 2080653 | T | A | 0.340 | 0.260 | 0.026 | 1.586e-23 | 9.999e-05 | 99.617 |
|  | rs41286699 | 16 | 2086191 | C | T | 0.077 | 0.254 | 0.064 | 7.943e-05 | 2.130e-05 | 15.555 |
|  | rs185056867 | 16 | 2088519 | T | C | 0.024 | 0.652 | 0.090 | 4.321e-13 | 5.345e-05 | 52.482 |
|  | rs72766651 | 16 | 2100923 | A | G | 0.020 | 0.489 | 0.115 | 2.017e-05 | 2.489e-05 | 18.174 |
|  | rs139982960 | 16 | 2115033 | C | T | 0.065 | 0.343 | 0.055 | 4.923e-10 | 3.914e-05 | 38.706 |
|  | rs138036227 | 16 | 2148271 | A | G | 0.101 | -0.226 | 0.042 | 6.924e-08 | 2.932e-05 | 29.093 |
|  | rs140869992 | 16 | 2158570 | A | G | 0.014 | -1.107 | 0.142 | 6.025e-15 | 8.333e-05 | 60.849 |
|  | rs9935429 | 16 | 2210407 | A | G | 0.062 | 0.337 | 0.053 | 2.249e-10 | 4.027e-05 | 40.207 |

Abbreviations: SNP, Single Nucleotide Polymorphisms; Chr, Chromosome; Pos, Position; EA, Effect Allele; OA, Other Allele; EAF, Effect Allele Frequency; Beta, Effect estimate; SE, Standard Error; R2, proportion of variance explained; F, F-statistic

Appendix Table 24. Associations of cis single-nucleotide polymorphisms (SNPs) used as instruments for centrally acting antihypertensives from the systolic blood pressure GWAS by Keaton et al. ^2^

| Gene | SNP | Chr | Pos | EA | OA | EAF | Beta | SE | P-value | R2 | F |
| --- | --- | --- | --- | --- | --- | --- | --- | --- | --- | --- | --- |
| ADRA2A | rs61882777 | 10 | 111962102 | C | T | 0.096 | 0.219 | 0.042 | 2.052e-07 | 2.673e-05 | 26.961 |
|  | rs17127310 | 10 | 112137261 | A | G | 0.267 | -0.126 | 0.028 | 5.734e-06 | 2.033e-05 | 20.525 |
|  | rs11195157 | 10 | 112259493 | C | G | 0.328 | 0.112 | 0.026 | 1.394e-05 | 1.871e-05 | 18.890 |
|  | rs73356958 | 10 | 112485081 | T | C | 0.178 | 0.152 | 0.032 | 1.809e-06 | 2.260e-05 | 22.824 |
|  | rs2419580 | 10 | 112500340 | G | T | 0.814 | 0.152 | 0.031 | 1.083e-06 | 2.351e-05 | 23.738 |
|  | rs7894045 | 10 | 113511441 | G | A | 0.630 | -0.104 | 0.025 | 4.148e-05 | 1.683e-05 | 16.829 |
|  | rs6585121 | 10 | 113612431 | G | A | 0.438 | 0.106 | 0.024 | 1.268e-05 | 1.889e-05 | 19.077 |

Abbreviations: SNP, Single Nucleotide Polymorphisms; Chr, Chromosome; Pos, Position; EA, Effect Allele; OA, Other Allele; EAF, Effect Allele Frequency; Beta, Effect estimate; SE, Standard Error; R2, proportion of variance explained; F, F-statistic

Appendix Table 25. Associations of cis single-nucleotide polymorphisms (SNPs) used as instruments for loop diuretics from the systolic blood pressure GWAS by Keaton et al. ^2^

| Gene | SNP | Chr | Pos | EA | OA | EAF | Beta | SE | P-value | R2 | F |
| --- | --- | --- | --- | --- | --- | --- | --- | --- | --- | --- | --- |
| SLC12A2 | rs711365 | 5 | 126997355 | C | T | 0.176 | 0.131 | 0.032 | 4.926e-05 | 1.637e-05 | 16.526 |
|  | rs13356360 | 5 | 127247719 | C | T | 0.038 | 0.373 | 0.072 | 2.414e-07 | 2.667e-05 | 26.659 |
|  | rs2250127 | 5 | 127388844 | A | G | 0.240 | -0.184 | 0.029 | 1.373e-10 | 4.094e-05 | 41.346 |
|  | rs17676242 | 5 | 127589648 | A | G | 0.091 | 0.368 | 0.043 | 1.936e-17 | 7.164e-05 | 72.191 |
|  | rs78736765 | 5 | 127693997 | T | C | 0.149 | -0.187 | 0.034 | 5.103e-08 | 2.936e-05 | 29.614 |
|  | rs80097588 | 5 | 127729949 | G | A | 0.059 | 0.305 | 0.053 | 1.094e-08 | 3.242e-05 | 32.702 |
|  | rs331067 | 5 | 127792031 | A | G | 0.119 | -0.193 | 0.039 | 6.665e-07 | 2.445e-05 | 24.692 |
|  | rs77396757 | 5 | 127814611 | A | G | 0.063 | 0.295 | 0.050 | 4.077e-09 | 3.420e-05 | 34.533 |
|  | rs17677603 | 5 | 127857493 | G | A | 0.383 | 0.313 | 0.025 | 2.364e-36 | 1.577e-04 | 159.09 |
|  | rs115854947 | 5 | 127905720 | A | T | 0.037 | 0.309 | 0.068 | 5.388e-06 | 2.052e-05 | 20.723 |
|  | rs247095 | 5 | 128093756 | A | G | 0.743 | 0.166 | 0.028 | 2.950e-09 | 3.500e-05 | 35.230 |
|  | rs1012262 | 5 | 128111033 | G | T | 0.048 | 0.247 | 0.060 | 3.908e-05 | 1.677e-05 | 16.918 |
|  | rs3798119 | 5 | 128446836 | A | G | 0.199 | -0.125 | 0.030 | 4.059e-05 | 1.664e-05 | 16.799 |
|  | rs10063241 | 5 | 128509412 | G | A | 0.234 | 0.132 | 0.029 | 6.315e-06 | 2.015e-05 | 20.343 |
| SLC12A1 | rs4774498 | 15 | 47873605 | A | T | 0.664 | -0.102 | 0.025 | 5.665e-05 | 1.600e-05 | 16.221 |
|  | rs12915677 | 15 | 48864797 | T | C | 0.204 | 0.136 | 0.030 | 8.475e-06 | 1.945e-05 | 19.766 |
|  | rs689304 | 15 | 48922360 | T | C | 0.097 | -0.396 | 0.041 | 3.002e-22 | 9.263e-05 | 94.252 |
|  | rs7174374 | 15 | 48990866 | G | A | 0.631 | 0.106 | 0.027 | 9.063e-05 | 1.504e-05 | 15.273 |
|  | rs76629237 | 15 | 49180584 | T | C | 0.078 | -0.199 | 0.048 | 2.863e-05 | 1.718e-05 | 17.481 |

Abbreviations: SNP, Single Nucleotide Polymorphisms; Chr, Chromosome; Pos, Position; EA, Effect Allele; OA, Other Allele; EAF, Effect Allele Frequency; Beta, Effect estimate; SE, Standard Error; R2, proportion of variance explained; F, F-statistic

Appendix Table 26. Associations of cis single-nucleotide polymorphisms (SNPs) used as instruments for potassium-sparing diuretics and aldosterone antagonists from the systolic blood pressure GWAS by Keaton et al. ^2^

| Gene | SNP | Chr | Pos | EA | OA | EAF | Beta | SE | P-value | R2 | F |
| --- | --- | --- | --- | --- | --- | --- | --- | --- | --- | --- | --- |
| SCNN1A | rs10492093 | 12 | 5477030 | G | A | 0.119 | -0.148 | 0.037 | 6.659e-05 | 1.568e-05 | 15.935 |
|  | rs12303027 | 12 | 6795760 | A | T | 0.155 | -0.154 | 0.037 | 2.572e-05 | 1.775e-05 | 17.727 |
|  | rs2286729 | 12 | 6873818 | A | G | 0.083 | 0.192 | 0.044 | 1.276e-05 | 1.869e-05 | 19.014 |
| SCNN1BandG | rs7200484 | 16 | 23051365 | A | G | 0.672 | 0.112 | 0.026 | 1.768e-05 | 1.814e-05 | 18.437 |
|  | rs11641240 | 16 | 23232985 | C | T | 0.218 | 0.134 | 0.029 | 4.505e-06 | 2.067e-05 | 21.028 |
|  | rs75964872 | 16 | 23847575 | A | C | 0.197 | -0.135 | 0.030 | 9.237e-06 | 1.940e-05 | 19.733 |
|  | rs7405331 | 16 | 23881428 | A | G | 0.428 | -0.098 | 0.024 | 5.487e-05 | 1.600e-05 | 16.266 |
| SCNN1D | rs74460547 | 1 | 836684 | A | G | 0.020 | -0.524 | 0.112 | 3.023e-06 | 2.984e-05 | 21.789 |
|  | rs11516185 | 1 | 843405 | G | A | 0.358 | 0.171 | 0.042 | 4.036e-05 | 2.303e-05 | 16.816 |
|  | rs2710882 | 1 | 930923 | G | A | 0.806 | 0.157 | 0.036 | 1.686e-05 | 2.538e-05 | 18.533 |
|  | rs11260536 | 1 | 1108518 | C | T | 0.096 | -0.218 | 0.050 | 1.359e-05 | 2.590e-05 | 18.910 |
|  | rs2368566 | 1 | 1315044 | A | G | 0.920 | 0.328 | 0.058 | 1.199e-08 | 4.261e-05 | 32.506 |
|  | rs78919759 | 1 | 1446736 | C | T | 0.015 | -0.525 | 0.126 | 2.948e-05 | 2.390e-05 | 17.452 |
|  | rs28401288 | 1 | 1574709 | T | G | 0.294 | -0.136 | 0.032 | 2.058e-05 | 2.477e-05 | 18.089 |
|  | rs6604981 | 1 | 1599161 | G | A | 0.960 | 0.361 | 0.078 | 4.195e-06 | 2.903e-05 | 21.195 |
|  | rs7796 | 1 | 1684169 | G | C | 0.488 | -0.315 | 0.030 | 3.683e-26 | 1.442e-04 | 112.200 |
|  | rs61774994 | 1 | 1783438 | A | C | 0.069 | 0.286 | 0.073 | 8.465e-05 | 2.117e-05 | 15.454 |
|  | rs72634869 | 1 | 1849923 | A | C | 0.095 | -0.205 | 0.052 | 8.816e-05 | 2.110e-05 | 15.408 |
|  | rs28671104 | 1 | 1879563 | C | G | 0.118 | 0.197 | 0.049 | 5.614e-05 | 2.225e-05 | 16.246 |
|  | rs13303091 | 1 | 1916034 | T | C | 0.335 | 0.126 | 0.027 | 3.203e-06 | 2.335e-05 | 21.629 |
|  | rs3128290 | 1 | 2049508 | T | G | 0.603 | 0.192 | 0.025 | 1.712e-14 | 5.842e-05 | 58.798 |
|  | rs6656309 | 1 | 2149138 | G | C | 0.240 | 0.174 | 0.029 | 1.834e-09 | 3.598e-05 | 36.166 |
|  | rs6673129 | 1 | 2202774 | T | C | 0.153 | 0.176 | 0.039 | 6.144e-06 | 2.068e-05 | 20.470 |
| NR3C2 | rs13143677 | 4 | 148419040 | A | G | 0.296 | 0.167 | 0.026 | 3.117e-10 | 3.919e-05 | 39.571 |
|  | rs11945031 | 4 | 148515501 | A | G | 0.268 | -0.109 | 0.028 | 7.610e-05 | 1.556e-05 | 15.683 |

Abbreviations: SNP, Single Nucleotide Polymorphisms; Chr, Chromosome; Pos, Position; EA, Effect Allele; OA, Other Allele; EAF, Effect Allele Frequency; Beta, Effect estimate; SE, Standard Error; R2, proportion of variance explained; F, F-statistic

Appendix Table 27. Associations of cis single-nucleotide polymorphisms (SNPs) used as instruments for renin inhibitors from the systolic blood pressure GWAS by Keaton et al. ^2^

| Gene | SNP | Chr | Pos | EA | OA | EAF | Beta | SE | P-value | R2 | F |
| --- | --- | --- | --- | --- | --- | --- | --- | --- | --- | --- | --- |
| REN | rs33945931 | 1 | 203138304 | T | C | 0.061 | -0.256 | 0.051 | 5.676e-07 | 2.478e-05 | 25.02 |
|  | rs568 | 1 | 203713133 | A | G | 0.206 | 0.134 | 0.030 | 8.085e-06 | 1.988e-05 | 19.951 |
|  | rs74741500 | 1 | 204021799 | T | A | 0.193 | 0.157 | 0.031 | 4.235e-07 | 2.547e-05 | 25.517 |
|  | rs6700051 | 1 | 204071890 | G | C | 0.339 | 0.136 | 0.026 | 1.447e-07 | 2.748e-05 | 27.613 |
|  | rs61825748 | 1 | 204092502 | T | G | 0.051 | -0.283 | 0.057 | 7.390e-07 | 2.441e-05 | 24.529 |
|  | rs141660869 | 1 | 204113987 | C | A | 0.031 | -0.476 | 0.073 | 7.674e-11 | 4.193e-05 | 42.339 |
|  | rs3795573 | 1 | 204167672 | C | T | 0.146 | -0.177 | 0.035 | 3.602e-07 | 2.565e-05 | 25.899 |
|  | rs7550921 | 1 | 204862644 | C | A | 0.074 | -0.228 | 0.055 | 3.394e-05 | 2.200e-05 | 17.183 |
|  | rs2802827 | 1 | 204910356 | A | T | 0.548 | -0.102 | 0.024 | 2.779e-05 | 1.733e-05 | 17.481 |

Abbreviations: SNP, Single Nucleotide Polymorphisms; Chr, Chromosome; Pos, Position; EA, Effect Allele; OA, Other Allele; EAF, Effect Allele Frequency; Beta, Effect estimate; SE, Standard Error; R2, proportion of variance explained; F, F-statistic

Appendix Table 28. Associations of cis single-nucleotide polymorphisms (SNPs) used as instruments for thiazides and related diuretics from the systolic blood pressure GWAS by Keaton et al. ^2^

| Gene | SNP | Chr | Pos | EA | OA | EAF | Beta | SE | P-value | R2 | F |
| --- | --- | --- | --- | --- | --- | --- | --- | --- | --- | --- | --- |
| KCNMA1 | rs2894317 | 10 | 77660765 | A | G | 0.689 | -0.108 | 0.027 | 6.363e-05 | 1.583e-05 | 15.970 |
|  | rs2637245 | 10 | 78268859 | A | G | 0.806 | 0.146 | 0.031 | 2.226e-06 | 2.232e-05 | 22.325 |
|  | rs16933656 | 10 | 78307224 | G | A | 0.031 | -0.283 | 0.072 | 7.972e-05 | 1.545e-05 | 15.579 |
|  | rs1079037 | 10 | 79276849 | A | G | 0.082 | -0.183 | 0.044 | 3.558e-05 | 1.695e-05 | 17.121 |
|  | rs1781807 | 10 | 79708718 | G | A | 0.589 | 0.105 | 0.025 | 2.911e-05 | 1.737e-05 | 17.500 |
| SLC12A1 | rs4774498 | 15 | 47873605 | A | T | 0.664 | -0.102 | 0.025 | 5.665e-05 | 1.600e-05 | 16.221 |
|  | rs12915677 | 15 | 48864797 | T | C | 0.204 | 0.136 | 0.030 | 8.475e-06 | 1.945e-05 | 19.766 |
|  | rs689304 | 15 | 48922360 | T | C | 0.097 | -0.396 | 0.041 | 3.002e-22 | 9.263e-05 | 94.252 |
|  | rs7174374 | 15 | 48990866 | G | A | 0.631 | 0.106 | 0.027 | 9.063e-05 | 1.504e-05 | 15.273 |
|  | rs76629237 | 15 | 49180584 | T | C | 0.078 | -0.199 | 0.048 | 2.863e-05 | 1.718e-05 | 17.481 |
| SLC12A3 | rs13337678 | 16 | 56379937 | T | C | 0.568 | -0.105 | 0.025 | 2.091e-05 | 1.782e-05 | 18.114 |
|  | rs62036970 | 16 | 56771791 | C | T | 0.285 | -0.134 | 0.027 | 5.476e-07 | 2.461e-05 | 25.037 |
|  | rs76361196 | 16 | 56929557 | A | T | 0.122 | 0.156 | 0.038 | 3.131e-05 | 1.704e-05 | 17.324 |
|  | rs11643718 | 16 | 56933519 | A | G | 0.110 | -0.192 | 0.038 | 5.401e-07 | 2.470e-05 | 25.130 |
|  | rs11864751 | 16 | 56978148 | T | C | 0.275 | 0.137 | 0.027 | 5.232e-07 | 2.468e-05 | 25.110 |
| CA4 | rs146765323 | 17 | 57231195 | T | C | 0.014 | -0.54 | 0.129 | 2.825e-05 | 2.234e-05 | 17.537 |
|  | rs59549190 | 17 | 57384444 | A | G | 0.332 | -0.197 | 0.027 | 2.588e-13 | 5.282e-05 | 53.687 |
|  | rs1451506 | 17 | 57407019 | G | A | 0.885 | -0.159 | 0.040 | 6.537e-05 | 1.568e-05 | 15.920 |
|  | rs112460627 | 17 | 57940375 | T | C | 0.024 | 0.472 | 0.108 | 1.206e-05 | 2.622e-05 | 19.147 |
|  | rs1148559 | 17 | 57997527 | T | C | 0.193 | 0.230 | 0.031 | 5.534e-14 | 5.565e-05 | 56.495 |
|  | rs180531 | 17 | 58003358 | A | G | 0.721 | 0.188 | 0.031 | 1.179e-09 | 3.659e-05 | 37.017 |
|  | rs10853017 | 17 | 58076004 | T | C | 0.598 | -0.131 | 0.028 | 2.938e-06 | 2.175e-05 | 21.866 |
|  | rs1197070 | 17 | 58634810 | A | T | 0.182 | 0.183 | 0.035 | 1.775e-07 | 2.749e-05 | 27.301 |
|  | rs76907962 | 17 | 58681552 | A | C | 0.028 | 0.433 | 0.095 | 5.051e-06 | 2.853e-05 | 20.833 |
|  | rs79378385 | 17 | 58926996 | C | A | 0.044 | -0.324 | 0.072 | 5.728e-06 | 2.816e-05 | 20.560 |
|  | rs11868441 | 17 | 59239221 | G | A | 0.800 | -0.238 | 0.031 | 8.583e-15 | 5.936e-05 | 60.392 |

Abbreviations: SNP, Single Nucleotide Polymorphisms; Chr, Chromosome; Pos, Position; EA, Effect Allele; OA, Other Allele; EAF, Effect Allele Frequency; Beta, Effect estimate; SE, Standard Error; R2, proportion of variance explained; F, F-statistic

Appendix Table 29. Associations of cis single-nucleotide polymorphisms (SNPs) used as instruments for diazoxide (a vasodilator antihypertensive) from the systolic blood pressure GWAS by Keaton et al. ^2^

| Gene | SNP | Chr | Pos | EA | OA | EAF | Beta | SE | P-value | R2 | F |
| --- | --- | --- | --- | --- | --- | --- | --- | --- | --- | --- | --- |
| KCNJ11 | rs66906527 | 11 | 16400484 | T | A | 0.396 | -0.19 | 0.025 | 1.242e-14 | 5.883e-05 | 59.403 |
|  | rs4412728 | 11 | 16534954 | A | C | 0.324 | -0.116 | 0.026 | 1.209e-05 | 1.904e-05 | 19.207 |
|  | rs217754 | 11 | 16844274 | T | A | 0.210 | 0.189 | 0.031 | 6.988e-10 | 3.775e-05 | 37.987 |
|  | rs17770757 | 11 | 16855360 | A | T | 0.028 | -0.404 | 0.077 | 1.482e-07 | 2.733e-05 | 27.597 |
|  | rs416258 | 11 | 16911636 | C | G | 0.300 | 0.247 | 0.026 | 3.100e-21 | 8.848e-05 | 89.165 |
|  | rs61882040 | 11 | 16925553 | C | A | 0.022 | 0.450 | 0.093 | 1.429e-06 | 2.310e-05 | 23.223 |
|  | rs4756874 | 11 | 16947185 | T | C | 0.021 | 0.366 | 0.092 | 7.273e-05 | 1.568e-05 | 15.733 |
|  | rs113549924 | 11 | 16966711 | T | G | 0.066 | -0.211 | 0.051 | 3.034e-05 | 1.724e-05 | 17.405 |
|  | rs4757479 | 11 | 17021663 | A | C | 0.618 | -0.12 | 0.025 | 1.510e-06 | 2.289e-05 | 23.117 |
|  | rs141038443 | 11 | 17071001 | C | T | 0.021 | -0.47 | 0.111 | 2.226e-05 | 2.465e-05 | 18.001 |
|  | rs78770151 | 11 | 17151764 | C | A | 0.045 | 0.268 | 0.067 | 5.889e-05 | 1.606e-05 | 16.144 |
|  | rs12421951 | 11 | 17194104 | C | T | 0.046 | 0.407 | 0.062 | 6.166e-11 | 4.250e-05 | 42.763 |
|  | rs113343528 | 11 | 17211475 | C | T | 0.066 | 0.283 | 0.054 | 1.547e-07 | 2.738e-05 | 27.489 |
|  | rs10832778 | 11 | 17394073 | G | C | 0.621 | -0.318 | 0.025 | 2.790e-37 | 1.633e-04 | 163.200 |
|  | rs77889556 | 11 | 17498057 | A | G | 0.170 | -0.159 | 0.036 | 8.002e-06 | 1.977e-05 | 19.948 |
|  | rs4757587 | 11 | 17760904 | T | C | 0.844 | 0.166 | 0.04 | 3.919e-05 | 2.315e-05 | 16.901 |

Abbreviations: SNP, Single Nucleotide Polymorphisms; Chr, Chromosome; Pos, Position; EA, Effect Allele; OA, Other Allele; EAF, Effect Allele Frequency; Beta, Effect estimate; SE, Standard Error; R2, proportion of variance explained; F, F-statistic

Appendix Table 30. Associations of cis single-nucleotide polymorphisms (SNPs) used as instruments for fenoldopam (a vasodilator antihypertensive) from the systolic blood pressure GWAS by Keaton et al. ^2^

| Gene | SNP | Chr | Pos | EA | OA | EAF | Beta | SE | P-value | R2 | F |
| --- | --- | --- | --- | --- | --- | --- | --- | --- | --- | --- | --- |
| DRD5 | rs112430423 | 4 | 10026036 | C | T | 0.021 | -0.463 | 0.093 | 6.903e-07 | 2.449e-05 | 24.636 |
|  | rs4697704 | 4 | 10107789 | T | G | 0.697 | 0.123 | 0.026 | 2.681e-06 | 2.177e-05 | 21.979 |
|  | rs56383050 | 4 | 10379579 | T | C | 0.262 | 0.115 | 0.028 | 3.489e-05 | 1.695e-05 | 17.112 |
|  | rs73809635 | 4 | 10535382 | T | C | 0.021 | -0.416 | 0.104 | 5.808e-05 | 1.617e-05 | 16.163 |
| DRD1 | rs1965709 | 5 | 175248575 | C | T | 0.129 | -0.163 | 0.036 | 6.913e-06 | 1.999e-05 | 20.188 |
|  | rs7378584 | 5 | 175592404 | C | G | 0.523 | -0.116 | 0.024 | 1.992e-06 | 2.259e-05 | 22.679 |
|  | rs11956701 | 5 | 175825311 | G | A | 0.453 | 0.119 | 0.025 | 1.470e-06 | 2.296e-05 | 23.133 |

Abbreviations: SNP, Single Nucleotide Polymorphisms; Chr, Chromosome; Pos, Position; EA, Effect Allele; OA, Other Allele; EAF, Effect Allele Frequency; Beta, Effect estimate; SE, Standard Error; R2, proportion of variance explained; F, F-statistic

Appendix Table 31. Associations of cis single-nucleotide polymorphisms (SNPs) used as instruments for hydralazine (a vasodilator antihypertensive) from the systolic blood pressure GWAS by Keaton et al. ^2^

| Gene | SNP | Chr | Pos | EA | OA | EAF | Beta | SE | P-value | R2 | F |
| --- | --- | --- | --- | --- | --- | --- | --- | --- | --- | --- | --- |
| AOC3 | rs73311606 | 17 | 40226750 | A | G | 0.269 | -0.147 | 0.028 | 1.686e-07 | 2.747e-05 | 27.367 |
|  | rs5016637 | 17 | 40363296 | T | C | 0.828 | 0.142 | 0.036 | 8.048e-05 | 1.542e-05 | 15.581 |
|  | rs12150495 | 17 | 40388430 | A | G | 0.076 | 0.261 | 0.047 | 3.448e-08 | 3.067e-05 | 30.471 |
|  | rs4793069 | 17 | 40801187 | A | G | 0.940 | 0.295 | 0.053 | 3.161e-08 | 3.011e-05 | 30.601 |
|  | rs62079656 | 17 | 41426980 | T | G | 0.018 | -0.591 | 0.128 | 4.143e-06 | 2.905e-05 | 21.212 |
|  | rs12950756 | 17 | 41904372 | C | T | 0.072 | 0.239 | 0.050 | 1.728e-06 | 2.252e-05 | 22.863 |

Abbreviations: SNP, Single Nucleotide Polymorphism; Chr, Chromosome; Pos, Position; EA, Effect Allele; OA, Other Allele; EAF, Effect Allele Frequency; Beta, Effect estimate; SE, Standard Error; R2, proportion of variance explained; F, F-statistic

Appendix Table 32. Associations of cis single-nucleotide polymorphisms (SNPs) used as instruments for minoxidil (a vasodilator antihypertensive) from the systolic blood pressure GWAS by Keaton et al. ^2^

| Gene | SNP | Chr | Pos | EA | OA | EAF | Beta | SE | P-value | R2 | F |
| --- | --- | --- | --- | --- | --- | --- | --- | --- | --- | --- | --- |
| KCNJ1 | rs7117932 | 11 | 128436953 | T | C | 0.439 | -0.107 | 0.024 | 1.190e-05 | 1.894e-05 | 19.123 |
|  | rs11221386 | 11 | 128474091 | A | G | 0.183 | -0.149 | 0.032 | 2.143e-06 | 2.221e-05 | 22.404 |
|  | rs12420823 | 11 | 128563392 | T | C | 0.447 | -0.109 | 0.025 | 9.264e-06 | 1.941e-05 | 19.581 |
|  | rs635887 | 11 | 128578047 | G | T | 0.699 | -0.116 | 0.027 | 1.733e-05 | 1.827e-05 | 18.426 |
|  | rs7924416 | 11 | 128764180 | T | C | 0.262 | 0.134 | 0.029 | 4.033e-06 | 2.105e-05 | 21.204 |
|  | rs78907918 | 11 | 128767957 | C | T | 0.074 | -0.203 | 0.049 | 3.215e-05 | 1.710e-05 | 17.267 |
|  | rs7105028 | 11 | 128879432 | G | T | 0.369 | 0.107 | 0.025 | 2.219e-05 | 1.781e-05 | 17.987 |
|  | rs117146208 | 11 | 128894624 | A | T | 0.070 | 0.203 | 0.049 | 3.171e-05 | 1.713e-05 | 17.301 |
| PTGS1 | rs80257718 | 9 | 124934932 | T | C | 0.021 | -0.411 | 0.087 | 2.390e-06 | 2.187e-05 | 22.237 |
|  | rs7035162 | 9 | 125019507 | A | G | 0.496 | -0.108 | 0.024 | 1.030e-05 | 1.908e-05 | 19.410 |
|  | rs2416887 | 9 | 125335616 | C | G | 0.157 | 0.136 | 0.033 | 3.887e-05 | 1.667e-05 | 16.959 |
|  | rs4838021 | 9 | 125657099 | T | C | 0.130 | -0.242 | 0.036 | 3.156e-11 | 4.361e-05 | 43.995 |
|  | rs7032325 | 9 | 125917138 | A | G | 0.024 | -0.385 | 0.098 | 9.008e-05 | 1.939e-05 | 15.340 |
|  | rs77190872 | 9 | 126094061 | T | C | 0.117 | -0.175 | 0.041 | 1.660e-05 | 1.827e-05 | 18.515 |

Abbreviations: SNP, Single Nucleotide Polymorphism; Chr, Chromosome; Pos, Position; EA, Effect Allele; OA, Other Allele; EAF, Effect Allele Frequency; Beta, Effect estimate; SE, Standard Error; R2, proportion of variance explained; F, F-statistic

Appendix Table 33. Associations of cis single-nucleotide polymorphisms (SNPs) used as instruments for nitroprusside (a vasodilator antihypertensive) from the systolic blood pressure GWAS by Keaton et al. ^2^

| Gene | SNP | Chr | Pos | EA | OA | EAF | Beta | SE | P-value | R2 | F |
| --- | --- | --- | --- | --- | --- | --- | --- | --- | --- | --- | --- |
| NPR1 | rs35479618 | 1 | 153662423 | A | G | 0.017 | 0.924 | 0.115 | 1.076e-15 | 9.465e-05 | 64.278 |
|  | rs113191295 | 1 | 153912623 | A | G | 0.057 | -0.34 | 0.066 | 2.437e-07 | 3.650e-05 | 26.653 |
|  | rs6692026 | 1 | 154095372 | C | A | 0.337 | -0.101 | 0.025 | 6.995e-05 | 1.566e-05 | 15.812 |
|  | rs6427724 | 1 | 154502499 | A | T | 0.833 | 0.161 | 0.032 | 7.369e-07 | 2.436e-05 | 24.570 |

Abbreviations: SNP, Single Nucleotide Polymorphism; Chr, Chromosome; Pos, Position; EA, Effect Allele; OA, Other Allele; EAF, Effect Allele Frequency; Beta, Effect estimate; SE, Standard Error; R2, proportion of variance explained; F, F-statistic

Appendix Table 34. KCNJ11 variants associated with type 2 diabetes excluded from the sensitivity analysis of diazoxide and chronic periodontitis

Thirteen SNPs proxying diazoxide were queried against the GWAS Atlas (<https://atlas.ctglab.nl/>; accessed 16 January 2026), and variants associated with type 2 diabetes mellitus at p < 1×10⁻⁵ were excluded. Two SNPs met this criterion and were removed; Mendelian randomization analysis was repeated using the remaining instruments.

| SNP | atlas ID | PMID | Year | Trait | P-value | N | EA | OA |
| --- | --- | --- | --- | --- | --- | --- | --- | --- |
| rs113343528 | 4085 | 30297969 | 2018 | Type 2 Diabetes | 7.800e-06 | 898130 | C | T |
| rs12146652 | 4085 | 30297969 | 2018 | Type 2 Diabetes | 3.300e-22 | 898130 | C | A |
|  | 4086 | 30297969 | 2018 | Type 2 Diabetes (adjusted for BMI) | 2.100e-21 | 898130 | C | A |
|  | 4045 | 30054458 | 2018 | Type 2 Diabetes | 8.851e-15 | 659256 | C | A |
|  | 3328 | 31427789 | 2019 | Diabetes (diagnosed by doctor) | 9.756e-11 | 385420 | C | A |
|  | 3601 | 31427789 | 2019 | Non-cancer illness code, self-reported: diabetes | 2.752e-9 | 289307 | C | A |
|  | 3686 | 31427789 | 2019 | Diagnoses - secondary ICD10: E11 Type 2 diabetes mellitus | 1.284e-8 | 244890 | C | A |
|  | 4084 | 28566273 | 2017 | Type 2 Diabetes (adjusted for BMI) | 8.100e-7 | 159208 | C | A |
|  | 4083 | 28566273 | 2017 | Type 2 Diabetes | 1.300e-06 | 159208 | C | A |
|  | 3983 | 29358691 | 2018 | Type 2 Diabetes | 4.020e-06 | 70127 | C | A |

Abbreviations: SNP, Single Nucleotide Polymorphism; PMID, PubMed Identifier; EA, Effect Allele; OA, Other Allele; N, Sample size; BMI, Body Mass Index

Appendix Table 35. Heterogeneity statistics for inverse-variance weighted Mendelian randomization analyses of antihypertensive drug classes and chronic periodontitis

| Antihypertensive drug classes | Cochran’s Q | Degrees of freedom | P-value |
| --- | --- | --- | --- |
| Adrenergic neuron blockers | 2.903 | 4 | 0.574 |
| Alpha-adrenoceptor antagonists | 46.566 | 43 | 0.329 |
| Angiotensin-converting enzyme inhibitors | 11.096 | 13 | 0.603 |
| Angiotensin-II receptor blockers | 1.547 | 3 | 0.672 |
| Beta adrenoceptor antagonists | 25.628 | 34 | 0.849 |
| Calcium channel blockers | 67.351 | 80 | 0.842 |
| Centrally acting antihypertensives | 2.285 | 3 | 0.515 |
| Loop diuretics | 17.251 | 16 | 0.370 |
| Potassium-sparing diuretics and Aldosterone antagonists | 26.769 | 12 | 0.008 |
| Renin inhibitors | 5.338 | 5 | 0.376 |
| Thiazide and related diuretics | 21.120 | 12 | 0.049 |
| Vasodilator antihypertensives |  |  |  |
| Diazoxide | 9.087 | 12 | 0.696 |
| Fenoldopam | NA | NA | NA |
| Hydralazine | 8.641 | 10 | 0.567 |
| Minoxidil | 3.886 | 6 | 0.692 |
| Nitroprusside | 7.868 | 2 | 0.020 |

Appendix Table 36. Heterogeneity statistics for inverse-variance weighted Mendelian randomization analyses of antihypertensive drug classes and dental caries

| Antihypertensive drug | Cochran’s Q | Degrees of freedom | P-value |
| --- | --- | --- | --- |
| Adrenergic neuron blockers | 6.145 | 6 | 0.407 |
| Alpha-adrenoceptor antagonists | 49.601 | 52 | 0.569 |
| Angiotensin-converting enzyme inhibitors | 16.990 | 13 | 0.200 |
| Angiotensin-II receptor blockers | 3.078 | 3 | 0.38 |
| Beta-adrenoceptor antagonists | 61.664 | 39 | 0.012 |
| Calcium channel blockers | 99.368 | 102 | 0.555 |
| Centrally acting antihypertensives | 3.688 | 6 | 0.719 |
| Loop diuretics | 13.301 | 18 | 0.773 |
| Potassium-sparing diuretics & Aldosterone antagonists | 34.465 | 23 | 0.059 |
| Renin inhibitors | 7.469 | 7 | 0.382 |
| Thiazide and related diuretics | 17.498 | 25 | 0.863 |
| Vasodilator antihypertensives |  |  |  |
| Diazoxide | 9.915 | 15 | 0.825 |
| Fenoldopam | 6.253 | 5 | 0.282 |
| Hydralazine | 5.339 | 5 | 0.376 |
| Minoxidil | 23.07 | 13 | 0.041 |
| Nitroprusside | 2.034 | 3 | 0.565 |

Appendix Table 37. Bayesian colocalization results for 12 antihypertensive drug classes and chronic periodontitis

| Antihypertensive drug classes | Genes | PP.H0 | PP.H1 | PP.H2 | PP.H3 | PP.H4 |
| --- | --- | --- | --- | --- | --- | --- |
| Adrenergic neuron blockers | SLC18A2 | 3.06% | 80.60% | 0.56% | 14.8% | 0.91% |
|  | SLC6A2 | 71.30% | 11.40% | 14.70% | 2.35% | 0.29% |
| Alpha-adrenoceptor antagonists | ADRA1A | <0.01 % | 80.00% | <0.01 % | 19.30% | 0.76% |
|  | ADRA1D | <0.01 % | 68.50% | <0.01 % | 30.40% | 1.04% |
|  | ADRA1B | <0.01 % | 86.60% | <0.01 % | 12.90% | 0.49% |
|  | ADRA2A | 31.30% | 53.30% | 5.06% | 8.62% | 1.69% |
| Angiotensin-converting enzyme inhibitors | ACE | <0.01 % | 89.80% | <0.01 % | 9.56% | 0.64% |
| Angiotensin-II receptor blockers | AGTR1 | 3.40% | 77.30% | 0.79% | 17.91% | 0.61% |
| Beta-adrenoceptor antagonists | ADRB1 | <0.01 % | 87.30% | <0.01 % | 12.20% | 0.54% |
|  | ADRB2 | <0.01 % | 89.40% | <0.01 % | 10.00% | 0.63% |
| Calcium channel blockers | CACNA1C | 0.01 % | 82.80% | <0.01 % | 16.70% | 0.50% |
|  | CACNA2D1 | 73.60% | 3.43% | 21.80% | 1.01% | 0.14% |
|  | CACNA1S | 76.30% | 7.58% | 14.50% | 1.44% | 0.15% |
|  | CACNB2 | <0.01 % | 86.60% | <0.01 % | 12.80% | 0.58% |
|  | CACNA1D | <0.01 % | 83.30% | <0.01 % | 16.20% | 0.50% |
|  | CACNA1H | <0.01 % | 79.60% | <0.01 % | 19.80% | 0.55% |
| Centrally acting antihypertensives | ADARA2A | 31.30% | 53.30% | 5.06% | 8.62% | 1.69% |
| Loop diuretics | SLC12A1 | <0.01 % | 45.30% | <0.01 % | 54.30% | 0.40% |
|  | SLC12A2 | <0.01 % | 85.30% | <0.01 % | 10.80% | 3.94% |
| Potassium-sparing diuretics and  Aldosterone antagonists | SCNN1A | 67.70% | 23.60% | 6.10% | 2.12% | 0.51% |
|  | SCNN1B | 72.60% | 17.70% | 7.69% | 1.87% | 0.17% |
|  | SCNN1G | 73.40% | 18.00% | 6.83% | 1.67% | 0.16% |
|  | SCNN1D | <0.01 % | 93.90% | <0.01 % | 4.18% | 1.90% |
|  | NR3C2 | <0.01 % | 88.80% | <0.01 % | 10.20% | 0.97% |
| Renin inhibitors | REN | 1.53% | 82.40% | 0.25% | 13.60% | 2.21% |
| Thiazide and related diuretics | SLC12A3 | 4.39% | 79.40% | 0.77% | 13.90% | 1.56% |
|  | SLC12A1 | <0.01 % | 45.30% | <0.01 % | 54.30% | 0.40% |
|  | KCNMA1 | 70.10% | 15.20% | 11.70% | 2.53% | 0.46% |
|  | CA4 | <0.01 % | 85.80% | <0.01 % | 12.60% | 1.56% |
| Vasodilator antihypertensives | | | | | | |
| Diazoxide | KCNJ11 | <0.01 % | 86.70% | <0.01 % | 10.10% | 3.18% |
| Fenoldopam | DRD5 | 36.50% | 19.70% | 28.30% | 15.30% | 0.23% |
|  | DRD1 | 62.30% | 25.90% | 8.15% | 3.39% | 0.23% |
| Hydralazine | AOC3 | 0.96% | 92.80% | 0.05% | 4.8% | 1.35% |
| Minoxidil | KCNJ1 | 43.70% | 34.60% | 11.70% | 9.26% | 0.80% |
|  | PTGS1 | <0.01 % | 86.00% | <0.01 % | 12.80% | 1.24% |
| Nitroprusside | NPR1 | 17.80% | 73.20% | 1.48% | 6.11% | 1.40% |

PP, posterior probability; PP.H0, posterior probability of Hypothesis 0 (neither systolic blood pressure nor chronic periodontitis has a causal variant in the region) in the colocalization analysis; PP.H1, posterior probability of Hypothesis 1 (systolic blood pressure has a causal variant in the region) in the colocalization analysis; PP.H2, posterior probability of Hypothesis 2 (chronic periodontitis has a causal variant in the region) in the colocalization analysis; PP.H3, posterior probability of Hypothesis 3 (systolic blood pressure and chronic periodontitis have a distinct causal variant in the region) in the colocalization analysis; PP.H4, posterior probability of Hypothesis 4 (both systolic blood pressure and chronic periodontitis share a single causal variant in the region) in the colocalization analysis

Appendix Table 38. Bayesian colocalization results for 12 antihypertensive drug classes and dental caries

| Antihypertensive drug | Genes | PP.H0 | PP.H1 | PP.H2 | PP.H3 | PP.H4 |
| --- | --- | --- | --- | --- | --- | --- |
| Adrenergic neuron blockers | SLC18A2 | <0.01% | 86.00% | <0.01% | 12.20% | 1.77% |
|  | SLC6A2 | 61.60% | 9.38% | 24.90% | 3.79% | 0.35% |
| Alpha-adrenoceptor antagonists | ADRA1A | <0.01% | 70.20 | <0.01% | 29.40% | 0.46% |
|  | ADRA1D | <0.01% | 84.90% | <0.01% | 14.30% | 0.80% |
|  | ADRA1B | <0.01% | 85.50% | <0.01% | 13.90% | 0.56% |
|  | ADRA2A | 13.80% | 73.80% | 1.75% | 9.39% | 1.29% |
| Angiotensin-converting enzyme inhibitors | ACE | <0.01% | 90.40% | <0.01% | 8.98% | 0.60% |
| Angiotensin-II receptor blockers | AGTR1 | 1.35% | 73.80% | 0.44% | 23.90% | 0.49% |
| Beta-adrenoceptor antagonists | ADRB1 | <0.01% | 85.90% | <0.01% | 12.90% | 1.23% |
|  | ADRB2 | <0.01% | 80.40% | <0.01% | 18.80% | 0.77% |
| Calcium channel blockers | CACNA1C | <0.01% | 70.10% | <0.01% | 27.10% | 2.88% |
|  | CACNA2D1 | 74.00% | 5.69% | 18.00% | 1.39% | 0.87% |
|  | CACNA1S | 74.80% | 10.50% | 12.50% | 1.76% | 0.38% |
|  | CACNB2 | <0.01% | 76.20% | <0.01% | 23.20% | 0.61% |
|  | CACNA1D | <0.01% | 83.80% | <0.01% | 14.40% | 1.83% |
|  | CACNA1H | <0.01% | 84.70% | <0.01% | 14.70% | 0.54% |
| Centrally acting antihypertensives | ADARA2A | 13.80% | 73.80% | 1.75% | 9.39% | 1.29% |
| Loop diuretics | SLC12A1 | <0.01% | 89.70% | <0.01% | 9.04% | 1.31% |
|  | SLC12A2 | <0.01% | 70.40% | <0.01% | 29.00% | 0.62% |
| Potassium-sparing diuretics and  Aldosterone antagonists | SCNN1A | 62.60% | 22.80% | 10.30% | 3.75% | 0.49% |
|  | SCNN1B | 14.10% | 40.50% | 11.30% | 32.30% | 1.82% |
|  | SCNN1G | 14.20% | 40.60% | 11.20% | 32.20% | 1.82% |
|  | SCNN1D | <0.01% | 89.60% | <0.01% | 9.89% | 0.50% |
|  | NR3C2 | 0.02% | 86.90% | <0.01% | 12.10% | 0.94% |
| Renin inhibitors | REN | <0.01% | 83.70% | <0.01% | 14.70% | 1.58% |
| Thiazide and related diuretics | SLC12A3 | 8.50% | 62.50% | 3.38% | 24.80% | 0.83% |
|  | SLC12A1 | <0.01% | 89.70% | <0.01% | 9.04% | 1.31% |
|  | KCNMA1 | 24.20% | 54.10% | 6.42% | 14.30% | 0.95% |
|  | CA4 | <0.01% | 93.50% | <0.01% | 5.80% | 0.75% |
| Vasodilator antihypertensives | | | | | | |
| Diazoxide | KCNJ11 | <0.01% | 87.50% | <0.01% | 11.40% | 1.07% |
| Fenoldopam | DRD5 | 14.00% | 70.10% | 2.23% | 11.10% | 2.55% |
|  | DRD1 | 41.30% | 41.50% | 8.35% | 8.38% | 0.47% |
| Hydralazine | AOC3 | 1.16% | 91.10% | <0.01% | 6.27% | 1.35% |
|  |  |  |  |  |  |  |
| Minoxidil | KCNJ1 | 32.90% | 41.90% | 10.40% | 13.30% | 1.52% |
|  | PTGS1 | <0.01% | 86.90% | <0.01% | 12.00% | 1.12% |
| Nitroprusside | NPR1 | <0.01% | 83.80% | <0.01% | 11.90% | 4.30% |

PP, posterior probability; PP.H0, posterior probability of Hypothesis 0 (neither systolic blood pressure nor dental caries has a causal variant in the region) in the colocalization analysis; PP.H1, posterior probability of Hypothesis 1 (only systolic blood pressure has a causal variant in the region) in the colocalization analysis; PP.H2, posterior probability of Hypothesis 2 (only dental caries has a causal variant in the region) in the colocalization analysis; PP.H3, posterior probability of Hypothesis 3 (systolic blood pressure and dental caries have a distinct causal variant in the region) in the colocalization analysis; PP.H4, posterior probability of Hypothesis 4 (both systolic blood pressure and dental caries share a single causal variant in the region) in the colocalization analysis

Appendix Figure 1. Leave-one-out inverse-variance weighted analyses plot for adrenergic neuron blockers and chronic periodontitis.

Appendix Figure 2. Leave-one-out inverse-variance weighted analyses plot for angiotensin-converting enzyme inhibitors and chronic periodontitis.

Appendix Figure 3. Leave-one-out inverse-variance weighted analyses plot for alpha-adrenoceptor antagonists and chronic periodontitis.

Appendix Figure 4. Leave-one-out inverse-variance weighted analyses plot for angiotensin-II receptor blockers and chronic periodontitis.

Appendix Figure 5. Leave-one-out inverse-variance weighted analyses plot for beta-adrenoceptor antagonists and chronic periodontitis

Appendix Figure 6. Leave-one-out inverse-variance weighted analyses plot for calcium channel blockers and chronic periodontitis.

Appendix Figure 7. Leave-one-out inverse-variance weighted analyses plot for centrally acting antihypertensives and chronic periodontitis.

Appendix Figure 8. Leave-one-out inverse-variance weighted analyses plot for loop diuretics and chronic periodontitis.

Appendix Figure 9. Leave-one-out inverse-variance weighted analyses plot for potassium-sparing diuretics and aldosterone antagonists and chronic periodontitis.

Appendix Figure 10. Leave-one-out inverse-variance weighted analyses plot for renin inhibitors and chronic periodontitis.

Appendix Figure 11. Leave-one-out inverse-variance weighted analyses plot for thiazides and related diuretics and chronic periodontitis.

Appendix Figure 12. Leave-one-out inverse-variance weighted analyses plot for diazoxide and chronic periodontitis.

Appendix Figure 13. Leave-one-out inverse-variance weighted analyses plot for hydralazine and chronic periodontitis

Appendix Figure 14. Leave-one-out inverse-variance weighted analyses plot for minoxidil and chronic periodontitis

Appendix Figure 15. Leave-one-out inverse-variance weighted analyses plot for nitroprusside and chronic periodontitis

Appendix Figure 16. Leave-one-out inverse-variance weighted analyses plot for adrenergic neuron blockers and dental caries

Appendix Figure 17. Leave-one-out inverse-variance weighted analyses plot for angiotensin-converting enzyme inhibitors and dental caries

Appendix Figure 18. Leave-one-out inverse-variance weighted analyses plot for alpha-adrenoceptor antagonists and dental caries

Appendix Figure 19. Leave-one-out inverse-variance weighted analyses plot for angiotensin-II receptor blockers and dental caries

Appendix Figure 20. Leave-one-out inverse-variance weighted analyses plot for beta-adrenoceptor antagonists and dental caries

Appendix Figure 21. Leave-one-out inverse-variance weighted analyses plot for calcium channel blockers and dental caries

Appendix Figure 22. Leave-one-out inverse-variance weighted analyses plot for centrally acting antihypertensives and dental caries

Appendix Figure 23. Leave-one-out inverse-variance weighted analyses plot for loop diuretics and dental caries

Appendix Figure 24. Leave-one-out inverse-variance weighted analyses plot for potassium-sparing diuretics and aldosterone antagonists and dental caries

Appendix Figure 25. Leave-one-out inverse-variance weighted analyses plot for renin inhibitors and dental caries

Appendix Figure 26. Leave-one-out inverse-variance weighted analyses plot for thiazides and related diuretics and dental caries

Appendix Figure 27. Leave-one-out inverse-variance weighted analyses plot for diazoxide and dental caries

Appendix Figure 28. Leave-one-out inverse-variance weighted analyses plot for fenoldopam and dental caries

Appendix Figure 29. Leave-one-out inverse-variance weighted analyses plot for hydralazine and dental caries

Appendix Figure 30. Leave-one-out inverse-variance weighted analyses plot for minoxidil and dental caries

Appendix Figure 31. Leave-one-out inverse-variance weighted analyses plot for nitroprusside and dental caries

Appendix Figure 32. Positive control analyses for the antihypertensive drug classes and chronic periodontitis: Associations between genetically proxied antihypertensive drug classes and major coronary heart disease events and stroke

Odds ratios represent the effect of a genetically proxied 1 mmHg reduction in systolic blood pressure (SBP) through different antihypertensive drug classes on the risk of coronary heart disease (CHD) and stroke. SNPs (n) indicate the number of single-nucleotide polymorphisms used as instruments for each antihypertensive class. OR, odds ratio; CI, confidence interval

Appendix Figure 33. Positive control analyses for the antihypertensive drug classes and dental caries: Associations between genetically proxied antihypertensive drug classes and major coronary heart disease events and stroke Odds ratios represent the effect of a genetically proxied 1 mmHg reduction in systolic blood pressure (SBP) through different antihypertensive drug classes on the risk of coronary heart disease (CHD) and stroke. SNPs (n) indicate the number of single-nucleotide polymorphisms used as instruments for each antihypertensive class. OR, odds ratio; CI, confidence interval

STROBE-MR checklist of recommended items to address in reports of Mendelian randomization studies^1^ ^2^

| Item No. | Section | Checklist item | Page No. | Relevant text from manuscript |
| --- | --- | --- | --- | --- |
| 1 | TITLE and ABSTRACT | Indicate Mendelian randomization (MR) as the study’s design in the title and/or the abstract if that is a main purpose of the study |  | Abstract |
|  | INTRODUCTION |  |  |  |
| 2 | Background | Explain the scientific background and rationale for the reported study. What is the exposure? Is a potential causal relationship between exposure and outcome plausible? Justify why MR is a helpful method to address the study question |  | Introduction (paragraphs 3-6) |
| 3 | Objectives | State specific objectives clearly, including pre-specified causal hypotheses (if any). State that MR is a method that, under specific assumptions, intends to estimate causal effects |  | Introduction (paragraphs 5-7) |
|  | METHODS |  |  |  |
| 4 | Study design and data sources | Present key elements of the study design early in the article. Consider including a table listing sources of data for all phases of the study. For each data source contributing to the analysis, describe the following: |  |  |
|  | a) | Setting: Describe the study design and the underlying population, if possible. Describe the setting, locations, and relevant dates, including periods of recruitment, exposure, follow-up, and data collection, when available. |  | Materials and methods (2.1 Study Design), Figure 1 |
|  | b) | Participants: Give the eligibility criteria, and the sources and methods of selection of participants. Report the sample size, and whether any power or sample size calculations were carried out prior to the main analysis |  | Materials and methods (2.2. GWAS data sources) |
|  | c) | Describe measurement, quality control and selection of genetic variants |  | Materials and methods (2.2 GWAS data sources; 2.3 Instrument selection; 2.6.1 Mendelian randomization analyses) |
|  | d) | For each exposure, outcome, and other relevant variables, describe methods of assessment and diagnostic criteria for diseases |  | Materials and methods (2.2. GWAS data sources) |
|  | e) | Provide details of ethics committee approval and participant informed consent, if relevant |  |  |
| 5 | Assumptions | Explicitly state the three core IV assumptions for the main analysis (relevance, independence and exclusion restriction) as well assumptions for any additional or sensitivity analysis |  | Materials and methods (2.4. Instrument strength, paragraph 1) |
| 6 | Statistical methods: main analysis | Describe statistical methods and statistics used |  |  |
|  | a) | Describe how quantitative variables were handled in the analyses (i.e., scale, units, model) |  | Materials and methods (2.6.1 Mendelian randomization analyses, paragraph 3) |
|  | b) | Describe how genetic variants were handled in the analyses and, if applicable, how their weights were selected |  | Materials and methods (2.3. Instrument selection, paragraph 3; 2.6.1 Mendelian randomization analyses, paragraph 1) |
|  | c) | Describe the MR estimator (e.g. two-stage least squares, Wald ratio) and related statistics. Detail the included covariates and, in case of two-sample MR, whether the same covariate set was used for adjustment in the two samples |  | Materials and methods (2.6.1 Mendelian randomization analyses, paragraph 2) |
|  | d) | Explain how missing data were addressed |  | Materials and methods (2.6.1 Mendelian randomization analyses, paragraph 1) |
|  | e) | If applicable, indicate how multiple testing was addressed |  | Materials and methods (2.6.1 Mendelian randomization analyses, paragraph 3) |
| 7 | Assessment of assumptions | Describe any methods or prior knowledge used to assess the assumptions or justify their validity |  | Materials and methods (2.3. Instrument selection, paragraph 3; 2.4. Instrument strength; 2.5. Instrument validation; 2.6.2 Colocalization analysis) |
| 8 | Sensitivity analyses and additional analyses | Describe any sensitivity analyses or additional analyses performed (e.g. comparison of effect estimates from different approaches, independent replication, bias analytic techniques, validation of instruments, simulations) |  | Materials and methods (2.5. Instrument validation; 2.6.1 Mendelian randomization analyses, paragraph 3) |
| 9 | Software and pre-registration |  |  |  |
|  | a) | Name statistical software and package(s), including version and settings used |  | Materials and methods (2.6.2 Colocalization analysis, paragraph 3) |
|  | b) | State whether the study protocol and details were pre-registered (as well as when and where) |  | NA |
|  | RESULTS |  |  |  |
| 10 | Descriptive data |  |  |  |
|  | a) | Report the numbers of individuals at each stage of included studies and reasons for exclusion. Consider use of a flow diagram |  | Materials and methods (2.2. GWAS data sources) |
|  | b) | Report summary statistics for phenotypic exposure(s), outcome(s), and other relevant variables (e.g. means, SDs, proportions) |  | Materials and methods (2.2. GWAS data sources) |
|  | c) | If the data sources include meta-analyses of previous studies, provide the assessments of heterogeneity across these studies |  |  |
|  | d) | For two-sample MR:  i.  Provide justification of the similarity of the genetic variant-exposure associations between the exposure and outcome samples  ii.  Provide information on the number of individuals who overlap between the exposure and outcome studies |  | Materials and methods (2.1 Study design; 2.3 Instrument selection, paragraph 2) |
| 11 | Main results |  |  |  |
|  | a) | Report the associations between genetic variant and exposure, and between genetic variant and outcome, preferably on an interpretable scale |  | Appendix Tables 2-17 and 21-36 |
|  | b) | Report MR estimates of the relationship between exposure and outcome, and the measures of uncertainty from the MR analysis, on an interpretable scale, such as odds ratio or relative risk per SD difference |  | Figures 2 and 3, Results (3.1.1 Chronic periodontitis, paragraph 2) |
|  | c) | If relevant, consider translating estimates of relative risk into absolute risk for a meaningful time period |  |  |
|  | d) | Consider plots to visualize results (e.g. forest plot, scatterplot of associations between genetic variants and outcome versus between genetic variants and exposure) |  | Figures 2 and 3 |
| 12 | Assessment of assumptions |  |  |  |
|  | a) | Report the assessment of the validity of the assumptions |  | 2.4 Instrument strength, 3.3 Positive control analyses |
|  | b) | Report any additional statistics (e.g., assessments of heterogeneity across genetic variants, such as *I^2^*, Q statistic or E-value) |  | Appendix Tables 18 and 37 |
| 13 | Sensitivity analyses and additional analyses |  |  |  |
|  | a) | Report any sensitivity analyses to assess the robustness of the main results to violations of the assumptions |  | Results (3.1.1 Chronic periodontitis, paragraph 1) |
|  | b) | Report results from other sensitivity analyses or additional analyses |  | Results (3.1.1 Chronic periodontitis, paragraph 1; Appendix Figures 3-33) |
|  | c) | Report any assessment of direction of causal relationship (e.g., bidirectional MR) |  | NA |
|  | d) | When relevant, report and compare with estimates from non-MR analyses |  | Discussion (paragraph 3) |
|  | e) | Consider additional plots to visualize results (e.g., leave-one-out analyses) |  | Appendix Figures 3-33 |
|  | DISCUSSION |  |  |  |
| 14 | Key results | Summarize key results with reference to study objectives |  | Discussion (paragraph 1) |
| 15 | Limitations | Discuss limitations of the study, taking into account the validity of the IV assumptions, other sources of potential bias, and imprecision. Discuss both direction and magnitude of any potential bias and any efforts to address them |  | Discussion (paragraph 5) |
| 16 | Interpretation |  |  |  |
|  | a) | Meaning: Give a cautious overall interpretation of results in the context of their limitations and in comparison with other studies |  | Discussion (paragraphs 1-5) |
|  | b) | Mechanism: Discuss underlying biological mechanisms that could drive a potential causal relationship between the investigated exposure and the outcome, and whether the gene-environment equivalence assumption is reasonable. Use causal language carefully, clarifying that IV estimates may provide causal effects only under certain assumptions |  | Discussion (paragraph 5) |
|  | c) | Clinical relevance: Discuss whether the results have clinical or public policy relevance, and to what extent they inform effect sizes of possible interventions |  | Discussion (paragraph 5) |
| 17 | Generalizability | Discuss the generalizability of the study results (a) to other populations, (b) across other exposure periods/timings, and (c) across other levels of exposure |  | Discussion (paragraph 6) |
|  | OTHER INFORMATION |  |  |  |
| 18 | Funding | Describe sources of funding and the role of funders in the present study and, if applicable, sources of funding for the databases and original study or studies on which the present study is based |  | Funding |
| 19 | Data and data sharing | Provide the data used to perform all analyses or report where and how the data can be accessed, and reference these sources in the article. Provide the statistical code needed to reproduce the results in the article, or report whether the code is publicly accessible and if so, where |  | Data availability statements |
| 20 | Conflicts of Interest | All authors should declare all potential conflicts of interest |  | Declaration of conflicting interests |

This checklist is copyrighted by the Equator Network under the Creative Commons Attribution 3.0 Unported (CC BY 3.0) license.

1. Skrivankova VW, Richmond RC, Woolf BAR, Yarmolinsky J, Davies NM, Swanson SA, et al. Strengthening the Reporting of Observational Studies in Epidemiology using Mendelian Randomization (STROBE-MR) Statement. JAMA. 2021;under review.

2. Skrivankova VW, Richmond RC, Woolf BAR, Davies NM, Swanson SA, VanderWeele TJ, et al. Strengthening the Reporting of Observational Studies in Epidemiology using Mendelian Randomisation (STROBE-MR): Explanation and Elaboration. BMJ. 2021;375:n2233.

**References**

1.	Evangelou E, Warren HR, Mosen-Ansorena D, et al. Genetic analysis of over 1 million people identifies 535 new loci associated with blood pressure traits. *Nat Genet*. 2018;50(10):1412-1425. doi:10.1038/s41588-018-0205-x

2.	Keaton JM, Kamali Z, Xie T, et al. Genome-wide analysis in over 1 million individuals of European ancestry yields improved polygenic risk scores for blood pressure traits. *Nat Genet*. 2024;56(5):778-791. doi:10.1038/s41588-024-01714-w
